# Genotype B of deformed wing virus and related recombinant viruses become dominant in European honey bee colonies

**DOI:** 10.1038/s41598-025-86937-5

**Published:** 2025-02-08

**Authors:** Fabrice Sircoulomb, Eric Dubois, Frank Schurr, Pierrick Lucas, Marina Meixner, Alicia Bertolotti, Yannick Blanchard, Richard Thiéry

**Affiliations:** 1 ANSES Sophia Antipolis Laboratory, Unit of Honey Bee Pathology, Sophia Antipolis, 06902 France; 2 ANSES Ploufragan – Plouzané – Niort Laboratory, Unit of Viral Genetics and Biosecurity, Ploufragan, 22440 France; 3 LLH Bee Institute Kirchhain, 35274 Kirchhain, Germany

**Keywords:** Viral epidemiology, Molecular biology

## Abstract

The *Varroa destructor *mite’s transmission of deformed wing virus (DWV) to honey bees is responsible for most winter mortalities of colonies worldwide. Four DWV genotypes (A, B, C and D) and numerous recombinants have been described. The most recent studies have reported the greater prevalence of DWV-B over DWV-A in several countries, including European ones, while C and D genotypes appear rare or extinct. However, no global evaluation of DWV-A and DWV-B distribution was available at the European level to date. In this study, we quantified both DWV genotypes by real-time PCR from pools or individual honey bees and from *V. destructor *mites sampled in 15 European countries between 2010 and 2017. These data and the sequencing of the viral RNA provide a first insight into DWV diversity, with a clear dominance of DWV-B and recombinants (A/B) in Europe. Chimeric sequencing reads were used to locate the recombinant junctions along the DWV genome. These were not randomly distributed, but mainly clustered in three genomic areas: the 5’UTR, leader peptide and helicase coding sequences. In our study, the DWV recombinant genomes shared at least the VP1-VP3 coding sequences with the DWV-B. Further studies are needed to explore the apicultural context explaining these differences in DWV genotype dominance.

**Supplementary Information:**

The online version contains supplementary material available at 10.1038/s41598-025-86937-5.

## Introduction

The health of honey bee colonies is threatened by numerous parasites and pathogens, including viruses^1^. Deformed wing virus (DWV, species: *Iflavirus aladeformis*), originally regarded as a low-impact stressor to honey bees, is now considered as one of the main viral threats responsible for winter losses in honey bee colonies around the world^2,3^. The clinical impact of DWV on honey bee health began following the introduction of the *Varroa destructor* mite in the second part of the 20th century into Europe and America^3^. It is unclear whether DWV infected the western honey bee or not before the *V. destructor* mite switched hosts from *Apis cerana* (eastern cavity-nesting honey bee) to *Apis mellifera* (western honey bee)^4^. It is known that DWV can be transmitted vertically or horizontally by oral acquisition and persist as a covert infection even in the mite’s absence^5–9^. By directly inoculating virus particles into the haemolymph while feeding on larvae or adult honey bees, the *V. destructor* mite acts as a biological vector and significantly enhances the pathogenicity of DWV^10–12^. The horizontal transmission of DWV by the *V. destructor* mite has been shown to be associated with morphological symptoms such as deformed wings, a shortened abdomen, behavioural disorders such as foraging at an earlier age^13^, reduced distance and duration of flights^14^, a decrease in learning performance^15,16^ and a reduced lifespan^17^. It has also been suggested that the synergistic suppression of honey bee immunity by both the mite and the virus could be an additional factor leading to this change in DWV pathogenicity^18,19^. Moreover, the mite’s transmission of DWV has been associated with a reduction in DWV genetic diversity^10,12^ and a higher viral load observed in colonies and single honey bee heads^20^, suggesting the selection of better-adapted viral variants to this new context of host–pathogen–vector interactions^21–24^. The *V. destructor* mite has notably been associated with the spread of an emerging DWV-related virus, Varroa destructor virus-1 (VDV1)^9^. This emerging virus was genotyped as DWV-B, distinguishing it from the original DWV variants (genotyped as DWV-A)^25^. However, in the case of co-infection by DWV-A and DWV-B, DWV recombinants are produced^23^, establishing a phylogenetic bridge between the two main genotypes^24^. Genotypes DWV-A and DWV-B are most commonly detected^24–27^ whereas the most recently identified DWV genotypes, C and D, are considered rare or extinct, respectively^28^. Although DWV-C (or related recombinants), has been found in some countries, there is no clear data about its prevalence or impact on honey bee health^27–30^. Virulence analyses of the DWV-A and -B genotypes have suggested that DWV-B might be more virulent in adult bees^22,23,31^, even though no significant differences in the prevalence of clinical symptoms or death were observed after inoculating DWV-A or DWV-B into honey bee pupae^32,33^. These results support previous studies suggesting that the overall prevalence of virulent viruses in honey bee brood could be reduced over the period of *V. destructor* mite infestation^12,34,35^. These field and experimental studies have shown that the introduction of the *V. destructor* mite changes the viral landscape, resulting in reduced prevalence of the most virulent viruses such as the sacbrood virus (SBV) and acute bee paralysis virus (ABPV), with its related viruses, the Kashmir bee virus (KBV) and Israeli acute paralysis virus (IAPV). The authors also suggested that a negative selection on *V. destructor* promoting the most virulent forms of DWV could impair pupae survival and, therefore, mite reproduction. Nevertheless, the main difference between DWV genotypes A and B is their ability to multiply in the *V. destructor* mite. Posada-Florez et al. (2019) observed that DWV-A did not replicate in the mite and that DWV-A spread by the mite was correlated with highly-efficient viral transmission by inoculation during mite feeding^36^. In contrast, two studies have confirmed that DWV-B does replicate in the mite^37,38^. Gusachenko et al. (2020) investigated a recombinant genome (5’ non-coding sequences and structural protein-coding sequences from DWV-B and non-structural protein-coding sequences from DWV-A) and suggested that the genomic organisation of several DWV recombinants could be a key factor for replication in both the mite and the honey bee^38^. A recent study of the antiviral RNA interference pathway of *V. destructor* suggested that both DWV-A and DWV-B replicate in the mite but DWV-A infections may be more efficiently suppressed by mite antiviral defences^39^. Moreover, this finding supports the enhanced capability of DWV-B and several other recombinant DWVs to be vectorised by the mite, amplifying the virus in salivary glands for inoculation above all into bee larvae^37^. Since its first detection in Europe or the USA, the prevalence of DWV-B has increased over time^40^. Ryabov et al. (2017) estimated that DWV-B spread within the United States between 2010 and 2016 ^41^. Nevertheless, DWV-A was still the most prevalent genotype in samples collected in 2016. In Europe, where DWV-B spread earlier, the dominance of DWV-B over DWV-A was reported in Germany between 2016 and 2017 ^42^, in the United Kingdom in 2017, and in Italy in 2019 ^40^, and is considered to have taken place in 2014 in France^43^. Therefore, the prevalence of DWV genotypes may differ among countries, resulting in an initial bottleneck by selection of the *Varroa*-adapted DWV variants followed by viral diversification, thus evading antiviral RNA interference^44^. Natsopoulou et al. (2017) regretted the lack of knowledge regarding the European distribution of DWV genotypes^45^.

**Fig. 1:**
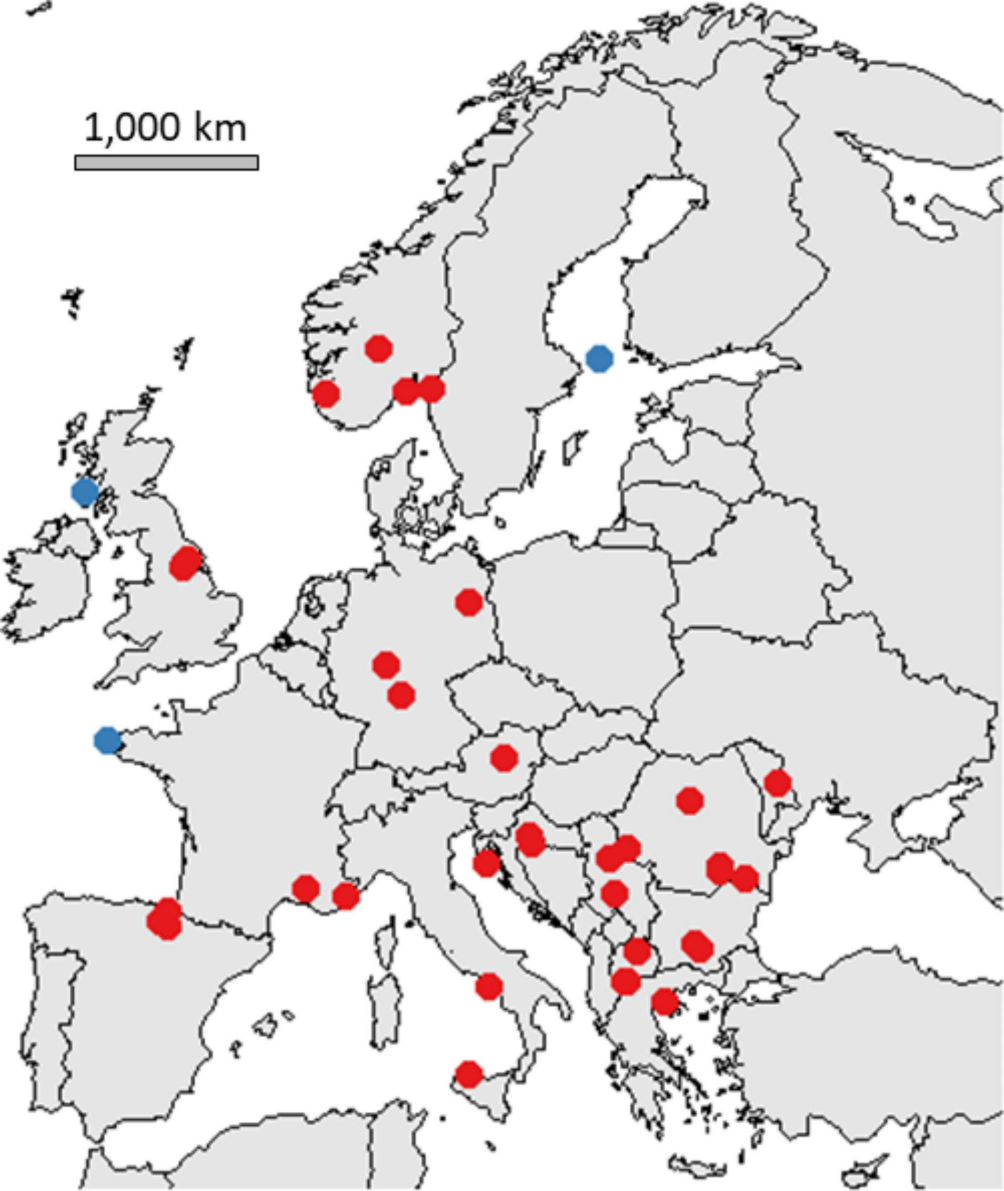
Geographical location of the 116 colonies included in this study. Each dot represents a colony positioned according to the city closest to the apiary. Colonies from *V. destructor-*free and -infested areas are represented in blue and red dots, respectively.

In this study, we first evaluated the variations in viral load of DWV genotypes A and B at the colony level using pooled bee samples, and at the individual level using single bee heads. All the samples were collected between 2010 and 2017 from 15 different European countries (Fig. 1). We then compared the viral loads observed in single bee heads from honey bees either with or without deformed wings, and in *V. destructor* mites. We selected a subset of samples and performed RNA sequencing to study the genomic structure of the DWV genotypes. We characterised recombinant and parental complete consensus genomic sequences. We mapped the recombinant junctions and performed a phylogenetic study to evaluate the genetic relationship between the DWV genotypes obtained from these different European locations. Our study highlights the increasing importance of DWV-B and related recombinant viruses in the honey bee viral landscape.

## Results

### Viral prevalence and loads of DWV-A and DWV-B in the RNA collection (honey bee samples collected between 2010 and 2011)

Deformed wing virus (DWV) detection and quantification at the honey bee colony level were estimated by two RT-qPCRs, specific for either the A- or B-genotype VP3 coding sequences, performed by analysing the RNA collection prepared from pooled bee heads (*n* = 44; Supplementary Figure S1A, Supplementary Table S1). No DWV-B was detected in samples from Austria. DWV-A was detected more frequently or at higher mean loads than DWV-B in samples from North Macedonia and Bulgaria. On the contrary, DWV-B was detected more frequently and at higher loads than DWV-A in samples from Germany and Croatia. Among the samples from Germany, 14 were from colonies treated against *V. destructor* mites (oxalic acid). These 14 samples had significantly lower mite infestation levels (Mann-Whitney U test, *p* = 3.9 × 10^− 7^) and either no detection or lower loads of DWV-A or DWV-B (max 4.8 log_10_ copies/bee) than untreated colonies (max 8.7 log_10_ copies/bee). Finally, we found a positive correlation (Spearman’s test, *p* = 9.4 × 10^− 7^) between the mite infestation level and the maximum viral load per bee head for DWV-A or DWV-B (Supplementary Figure S1B).

### Viral prevalence and loads of DWV-A and DWV-B in pooled samples of whole honey bees collected in Europe

**Fig. 2:**
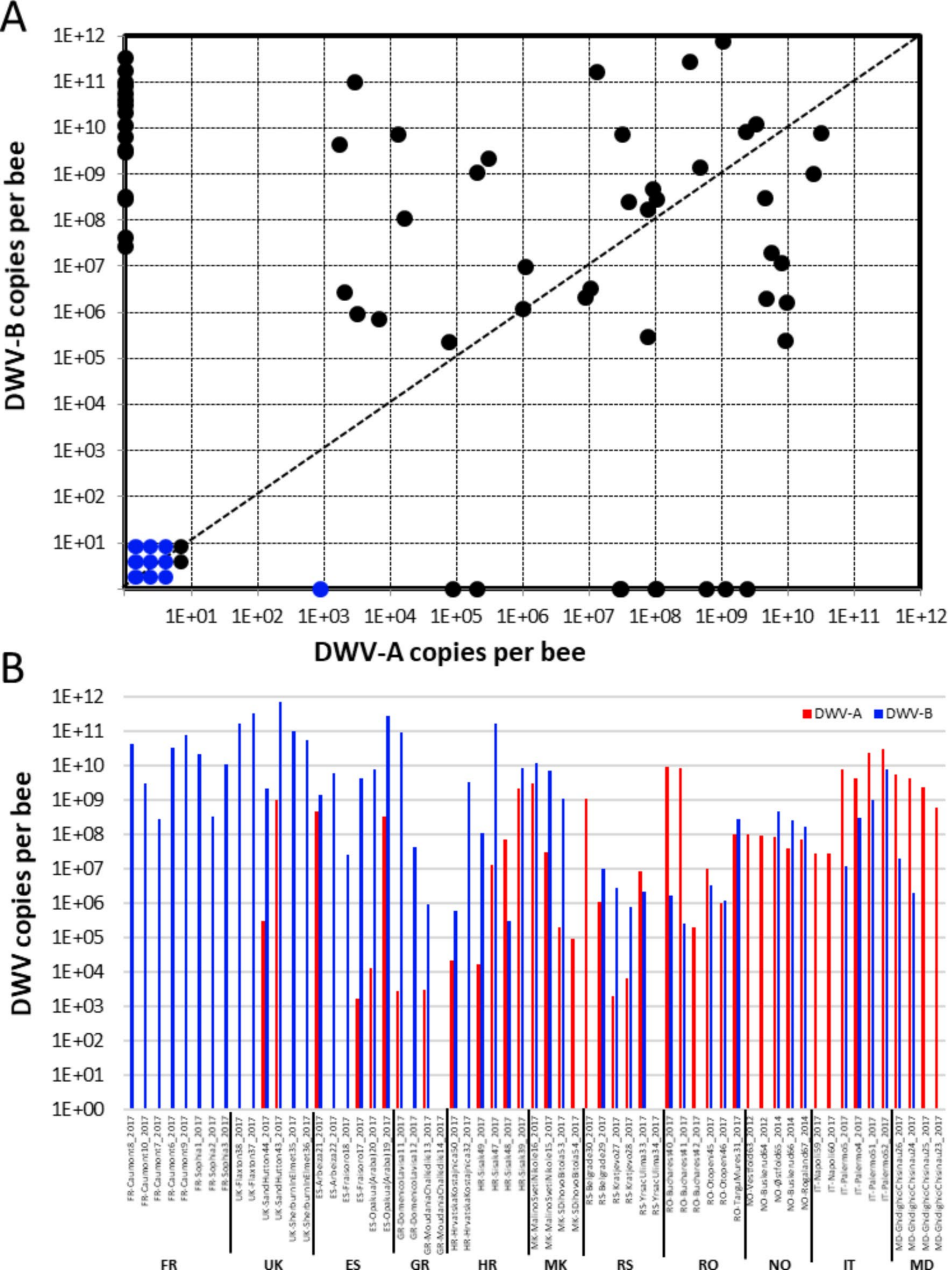
Colony-level viral loads of DWV-A and DWV-B estimated from honey bee pools. **A**: Variability of the observed viral loads. Each dot represents a pool of foragers from a colony according to DWV-A (x-axis) and DWV-B (y-axis) copies per bee. Colonies from mite-free or mite-infested areas are in blue and black dots, respectively. RT-qPCR quantifications were expressed in equivalents of viral genome copies per bee (copies per bee). **B**: Viral loads of DWV-A and DWV-B according to the country of origin. The red and blue bars represent the DWV-A and DWV-B loads, respectively. Viral loads were expressed in equivalents of viral genome copies per bee (copies per bee). Country codes are on the x-axis and initials are as follows: FR = France, UK = United Kingdom, ES = Spain, GR = Greece, HR = Croatia, MK = North Macedonia, RS = Republic of Serbia, RO = Romania, NO = Norway, IT = Italy, MD = Moldova.

The viral loads quantified in the pools of whole honey bee were analysed separately, as they were processed using different protocols (Fig. 2A and Supplementary Table S1).

DWV-B was not detected in any of the ten samples collected from the *V. destructor*-free European islands: Ushant (France; six colonies), Colonsay (Scotland; one colony) and Åland (Finland; three colonies). Only one sample from Ushant Island tested positive for DWV-A at a low viral load of 2.9 log_10_ copies/bee.

The observed prevalence of DWV-A was 70% (*n* = 43) and that of DWV-B was 82% (*n* = 50) after testing 61 pooled samples collected in *V. destructor*-infested areas. Four distinct patterns were observed: samples containing both DWV-A and DWV-B (56%, *n* = 34); samples with only DWV-A (15%, *n* = 9); samples with only DWV-B (26%, *n* = 16); and samples (3%, *n* = 2) with no DWV genotypes detected. The positive samples (*n* = 59) were quantified at viral load levels ranging from 2.9 to 10.5 log_10_ copies/bee for DWV-A, and from 5.4 to 11.9 log_10_ copies/bee for DWV-B. The mean viral loads in the infected pools were 7.2 and 8.7 log_10_ copies/bee for DWV-A and DWV-B, respectively. The DWV-B load was significantly (Mann-Whitney U test, *p* = 3.9 × 10^− 3^) higher than the DWV-A load. Additionally, we observed that the infected whole honey bees could be subdivided into low and high viral loads with thresholds set at 6.0 and 7.3 log_10_ copies/bee for DWV-A and DWV-B, respectively (Supplementary Figure S2). Using these thresholds, we observed exclusively high DWV-A load levels in 16 samples (27%) and high DWV-B load levels in 22 samples (37%). Both genotypes were observed at high viral load levels in 14 samples (24%). Finally, seven samples (12%) showed low levels of both DWV genotypes.

We investigated the relationship between DWV-A and DWV-B loads and the location of apiaries (Fig. 2B). DWV-B was detected in all the European countries included in this study. However, this genotype was detected at higher loads than DWV-A in samples from Greece, Spain, the United Kingdom and finally France, where no DWV-A was detected. In contrast, DWV-A was detected at higher loads than DWV-B in samples from Moldova, Italy, Norway and Romania. Samples collected in 2017 from Croatia, Macedonia or Serbia had similar DWV loads for both A and B genotypes.

### Viral load quantification in single honey bee heads and*V. destructor* mites

**Fig. 3:**
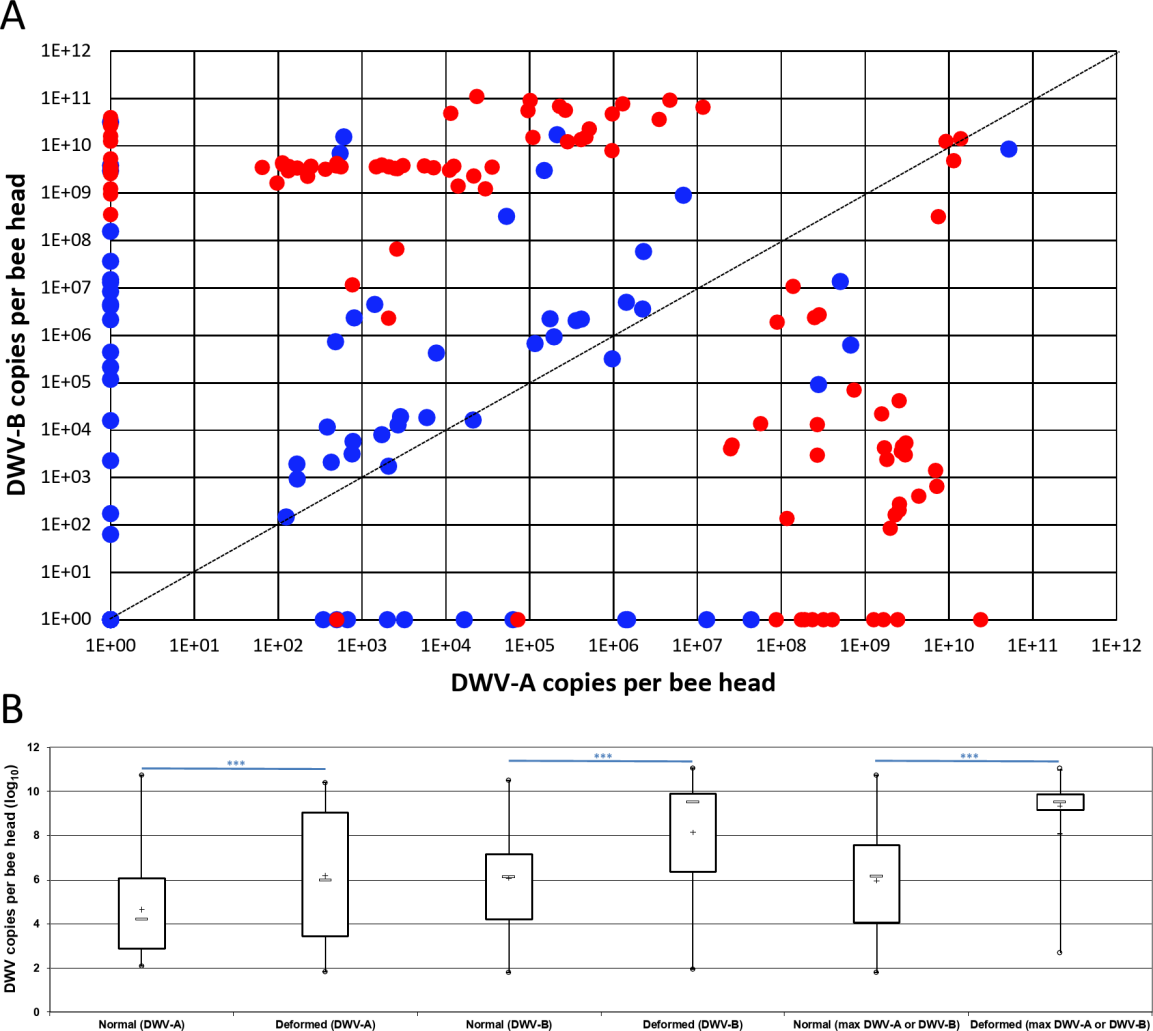
DWV-A and DWV-B loads quantified in single honey bee heads and *V. destructor*. **A**: Global distribution of viral loads measured in single heads from honey bees with deformed (red dots) and normal wings (blue dots). Each dot represents one head of a single honey bee according to DWV-A (x-axis) and DWV-B (y-axis) load level. RT-qPCR was performed on RNA extracted from single honey bee heads. RT-qPCR quantification levels were expressed in equivalents of viral genome copies per bee head (copies per bee head). **B**: DWV-A or DWV-B load (in log_10_) and maximum load from DWV-A and DWV-B quantified in single heads of honey bees with either normal or deformed wings. The box plots show the distribution of populations, with the first quartile (25%) and third quartile (75%) (box), the median (50%, white dash), the mean (cross), the minimal and maximal values (whiskers), and outliers (circle). ***: Mann-Whitney U test, *p* < 0.001.

Both DWV-A and DWV-B viral loads were quantified by RT-qPCR on RNA extracted from the single heads of honey bees with either deformed or normal wings (Fig. 3A, Supplementary Figure S3, and Supplementary Table S1). The relative abundances of both viruses were consistent between honey bee heads and the viral load quantified in the pooled sample from the same colony. The viral loads found in single heads were distributed over a wider range than those in the pooled honey bees, specifically for the lower viral loads. Viral loads ranged from 1.8 to 10.7 and from 1.8 to 11.0 log_10_ copies/head for DWV-A and DWV-B, respectively. The DWV-B load (mean = 7.4 log_10_ copies/head) was significantly higher (Mann-Whitney U test, *p* = 1.5 × 10^− 8^) than the DWV-A load (mean = 5.7 log_10_ copies/head). We examined the relationship between the country of origin and the distribution of DWV genotype viral loads in honey bee heads (Supplementary Table S1). DWV-B viral loads were higher than DWV-A loads in the heads of honey bees originating from France (four colonies), the United Kingdom (two colonies), Spain, Germany and Macedonia. In contrast, DWV-A was detected at higher load levels than DWV-B in samples from Moldova, Italy and Romania.

Taking into account the observed wing morphology of the tested honey bees, DWV-A or DWV-B loads were significantly higher (Mann-Whitney U test, *p* = 4.9 × 10^− 5^ or *p* = 2.9 10^− 7^, respectively) in the head of honey bees with deformed wings (*n* = 109; DWV-A mean load = 5.1 log_10_ copies/head; DWV-B mean load = 7.2 log_10_ copies/head) than in the head of honey bees with normal wings (*n* = 74; DWV-A mean load = 3.0 log_10_ copies/head; DWV-B mean load = 4.4 log_10_ copies/head) (Fig. 3B and Supplementary Table S1). The greater virus load in honey bees with deformed wings (mean = 9.3 log_10_ copies/head) than in honey bees with normal wings (mean = 5.2 log_10_ copies/head) was more striking when using the maximum virus load from DWV-A or DWV-B (Mann-Whitney U test, *p* = 0). Finally, the results showed that DWV-B viral loads were significantly higher than DWV-A viral loads in the infected heads of honey bees with either normal wings (Mann-Whitney U test, *p* = 3.5 × 10^− 3^) or deformed wings (Mann-Whitney U test, *p* = 1.9 × 10^− 7^).

Viral loads in bee heads and *V. destructor* mites were quantified for 11 colonies (Supplementary Figure S3 and supplementary Table S1). We observed that the relative proportion of DWV-A and DWV-B, as well as the viral loads, were concordant between *V. destructor* mites and single bee heads for five colonies: ES-Arbeiza21, FR-Caumont8, MK-MalinoSvetiNikole15, RO-TarguMures31 and UK-SherburnInElmet35.

### Honey bee virus sequencing

**Fig. 4:**
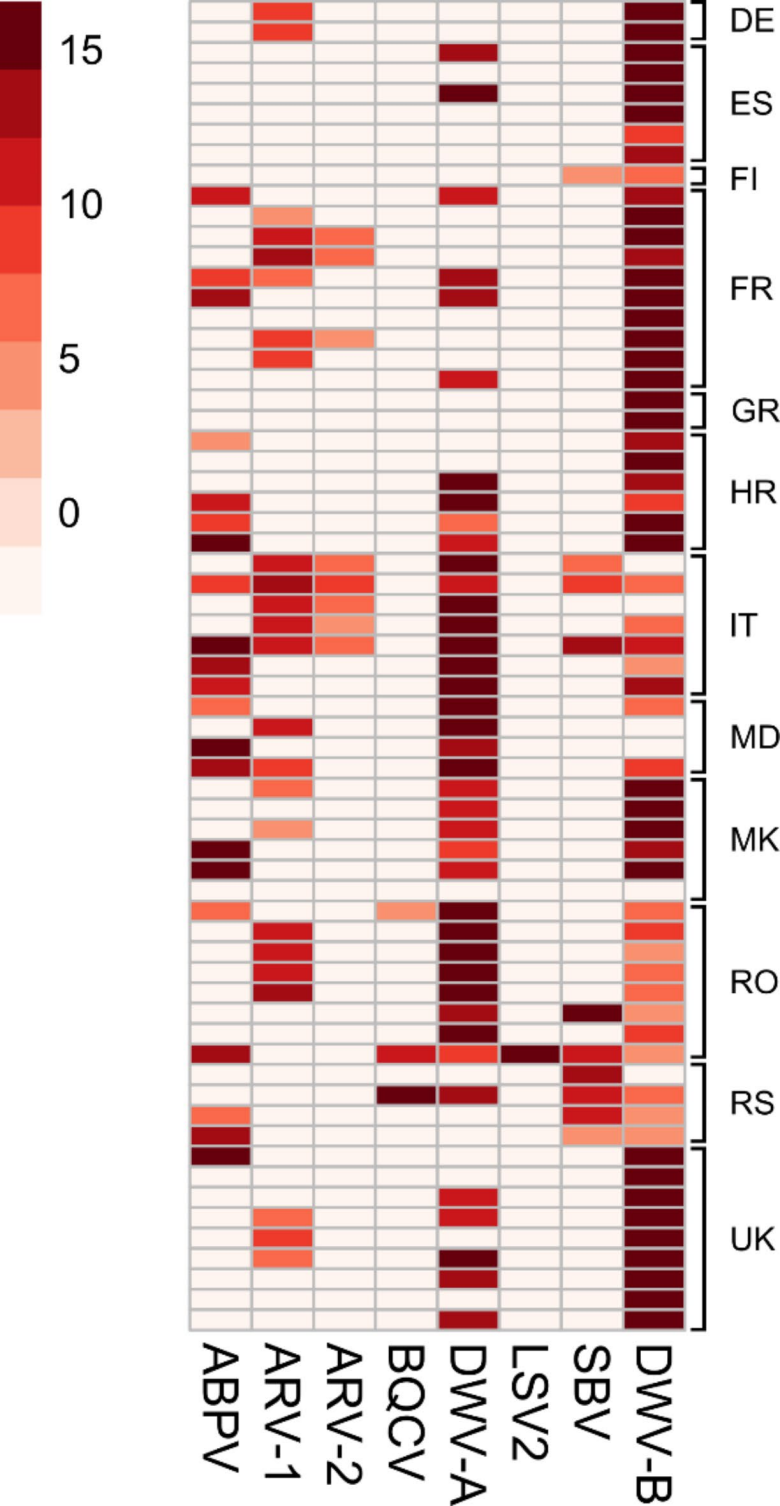
Detection of honey bee virus reads by next-generation sequencing. The reads obtained from bee pools or single bee heads were aligned against the genomes of known honey bee viruses. The sequences of eight viral species were covered to above 80%. The darkness of the red indicates the coverage depth (scale from 0 to 15). The results are given by country: UK = United Kingdom, RS = Republic of Serbia, RO = Romania, MK = North Macedonia, MD = Moldova, IT = Italy, HR = Croatia, GR = Greece, FR = France, FI = Finland, ES = Spain, DE = Germany.

In order to obtain deeper insights into the variability of DWV genomes, we selected and sequenced 93 isolates. The reads were aligned against already known honey bee viruses (Fig. 4). DWV-B was the most prevalent virus (94%, *n* = 87), while DWV-A was identified in 74% (*n* = 69) of the samples. Moreover, by analysing the sequencing data, we were able to identify other honey bee viruses within our samples. We found ABPV (23%, *n* = 21), apis rhabdovirus type 1 (ARV-1; 26%, *n* = 24) and type 2 (ARV-2; 9%, *n* = 8), black queen cell virus (BQCV; 3%, *n* = 3), lake Sinai virus type 2 (LSV-2; 1%, *n* = 1) and SBV (11%, *n* = 10).

### Analysis of DWV genotypes

**Fig. 5:**
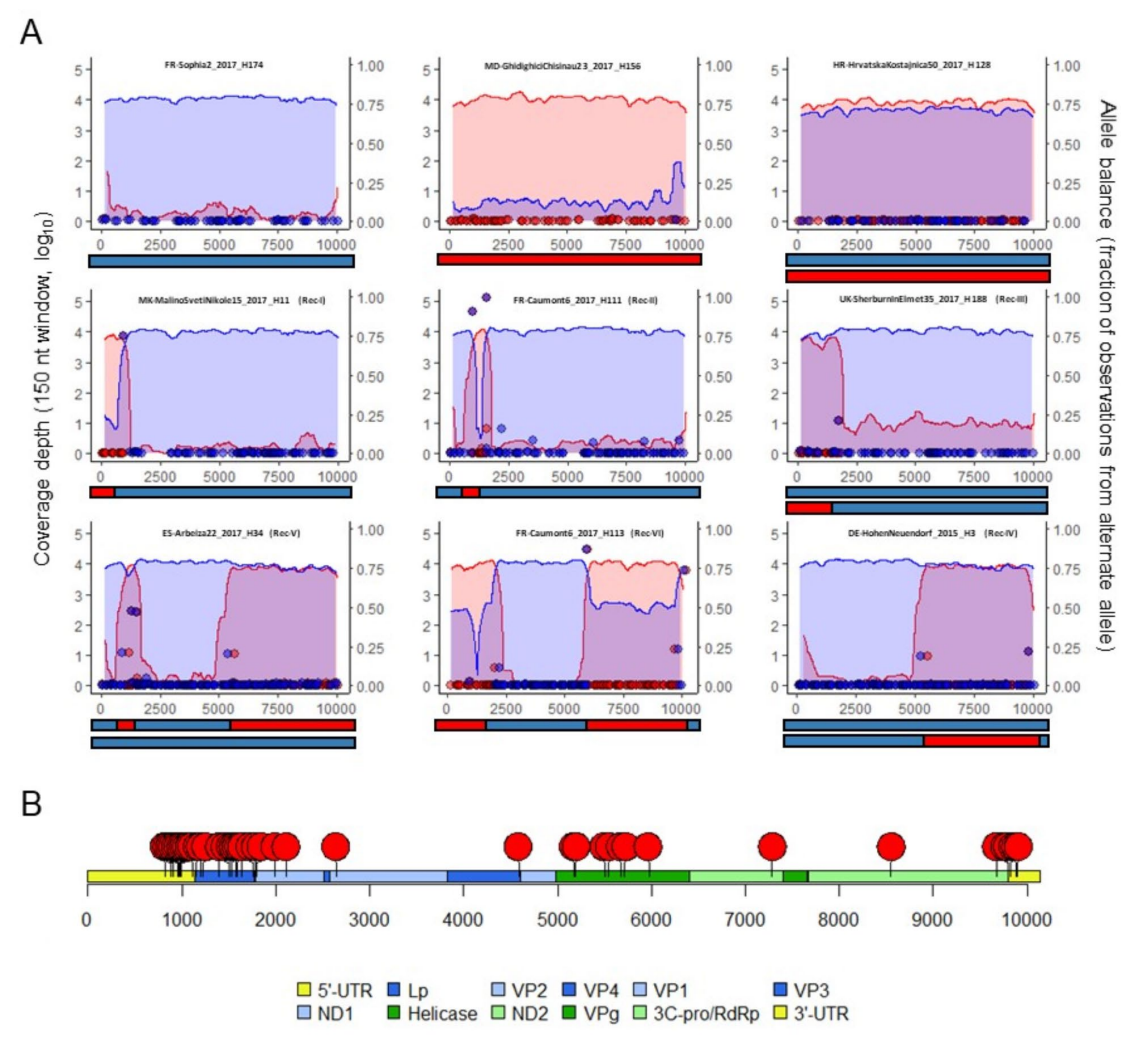
Genome profiles and recombinant junctions. **A**: Example of coverage depth and LUMPY profiles observed in six single bee heads. DWV-A and DWV-B coverage depth in log_10_ scale calculated in 150 nucleotide windows along the genome are represented by red and blue graphs, respectively. Dots represent the allele balance of the break end identified by LUMPY and SVtyper for DWV-A (red) and DWV-B (blue). The main genomes deduced are symbolised below each graph. **B**: Positions of the recombination junctions along the DWV-A genome. Each red dot (located on the viral genome by the black line) represents a recombination junction identified in DWV recombinant. The genomic domains^46^ of DWV-A are colour coded and ordered in the legend below the graph according to their position from the 5’-end to the 3’-end.

Next-generation sequencing (NGS) provided clear evidence of recombinant genomes containing fragments from both DWV-A and DWV-B, with a coverage depth around 4 log_10_ (Fig. 5A and Supplementary Figure S4). DWV recombinants junctions were defined using LUMPY, further confirmed by ViReMa analysis, and the variation in both DWV-A and DWV-B reference genome coverage depth profiles (Fig. 5B, Supplementary Figure S4 and Supplementary Table S2). LUMPY identified 85 recombinant junctions from 26 samples. Sixty-seven of these were also observed in ViReMa results. The average difference between the position junctions identified by LUMPY and by ViReMa was 79 and 60 nucleotides on DWV-A and DWV-B genomes, respectively. Among the identified junctions, 49 were used to guide the reconstruction of the recombinant genomes. Thirty one were located within a single contig aligning partially to DWV-A and DWV-B (Supplementary Table S2). Six main types of DWV recombinant profiles were identified (Rec-I to Rec-VI, Fig. 5A) based on the DWV-A sequence observed in the recombinant genomes. The DWV-A genomic fragments involved in the recombinant genomes often overlapped the 5’UTR, L-protein domain, non-structural domain and more rarely the 3’UTR domain. In contrast, DWV-B genomic fragments from the L-protein domain to the helicase were more often observed in recombinant genomes. Consequently, recombinant junctions were not randomly distributed and clustered in three genomic areas along DWV-A and DWV-B genomes (Fig. 5B). The first region is located between nucleotides 820–2201 of the DWV-A genome sequence. It overlaps with the 5’UTR, L-protein and VP2 domains. The second region was located at positions 4590–6003, mainly overlapping with the helicase domain. The third region was located at positions 9628–9891, overlapping with the end of the 3 C-pro/RdRp and 3’UTR domain. A few additional breakpoints were also found outside these regions (positions 2645, 7282 and 8547).

After sequencing RNA from honey bee pools from varroa-free areas, it was not possible to identify the recombinant junctions because the average DWV-A or B coverage was too low. Nevertheless, there was an increase in the coverage depth of the non-structural portion of the DWV-A genome in the pooled sample of honey bees from the Åland Islands, suggesting the presence of a DWV recombinant genome (Supplementary Figure S4).

We then investigated the relationship between the different DWV genotypes assembled from our samples (Supplementary Tables S3 and S4). We found a significant association between the absence of DWV-A and the detection of DWV recombinants (chi-square test, *p* = 7.9 × 10^− 6^) or DWV-B (chi-square test, *p*-value = 1.6 × 10^− 3^). An analysis of the pooled samples revealed that DWV-A was the main genotype in eight colonies from Italy, Moldova, the Republic of Serbia and Romania. DWV-B was the main genotype in nine colonies from Croatia, France, Greece, Macedonia, Spain and the United Kingdom. Both genotypes were also observed with equal coverage depth in two colonies, one from Croatia and the other from Italy. One DWV recombinant was observed to be dominant in three colonies from the same apiary in France. DWV recombinants and DWV-B genotypes were found to have the same coverage depth in colonies from France, Greece, Macedonia, Spain and the United Kingdom. Lastly, we also observed one colony from Italy where DWV-A, DWV-B and DWV recombinants were detected with equal coverage depth.

Results from recombinant junctions and variation in coverage depth profiles from single bee head sequencing data were similar to those found at the colony level (by testing pooled honey bee samples) (Fig. 5, Supplementary Figure S4). Several coverage depth profiles (e.g. ES-Arbeiza22_2017_H62) suggested the presence of multiple DWV recombinants co-existing in the same honey bee head. Genomes isolated from heads of honey bees with either deformed or normal wings were found in the entire spectrum of DWV genotypes (DWV-A, DWV-B and DWV recombinants) without any specific association.

### Phylogenetic analysis of DWV genomes

**Fig. 6:**
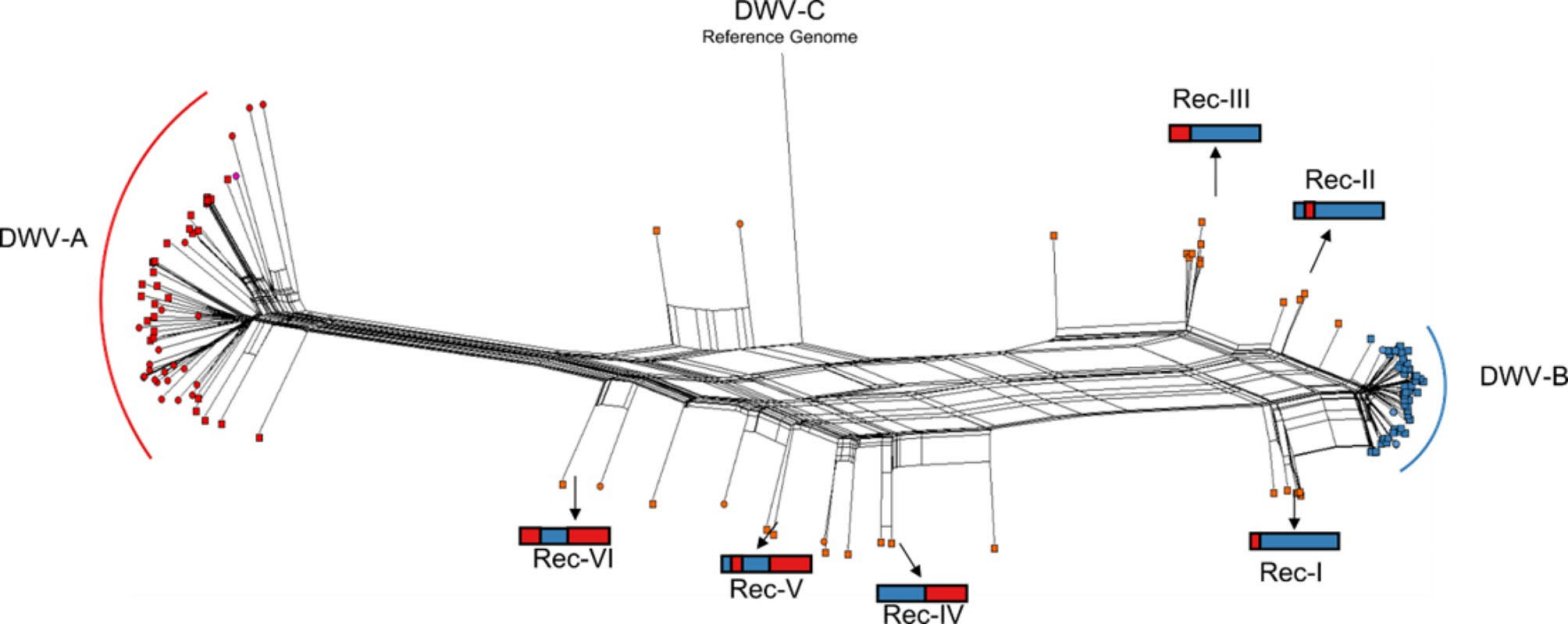
Neighbour-net splits graph of DWV genotype genomes. The dashed line represents the main split. The scale bar represents the unit for the expected number of substitutions per site. Red, blue, orange and pink correspond to DWV-A, DWV-B, DWV recombinant and Kakugo virus genomes, respectively. Oval and rectangular shapes represent the reference and reconstructed genomes, respectively. The long, vertical branch connects to the DWV-C reference genome and was split for clarification. The different types of recombinant genomes are symbolised together with their position in the graph.

To investigate the phylogeographic relationships of the DWV genotypes, a neighbour-net splits graph (Fig. 6) and a maximum likelihood tree were constructed (Supplementary Figure S5). The neighbour-net splits graph showed a main split separating the DWV-A genomes from the reticulated topologies containing the recombinants and the DWV-B genomes. The branches within the DWV-A cluster were longer than in the DWV-B cluster, suggesting more diversity in the former. The DWV recombinant cloud was subdivided into two clusters: one clustering Rec-I, II and III genomes close to DWV-B, and the second clustering Rec-IV, V and VI in a median position between DWV-A and DWV-B. DWV recombinants clustering in similar groups were observed in samples from different countries. For example, recombinant genomes of type Rec-I were found in samples collected in North Macedonia and Spain, and recombinant genomes of type Rec-III were found in samples collected in France, Spain and the United Kingdom.

In the maximum likelihood tree, we frequently found clusters of DWV genotypes from the same colony or the same apiary. Less frequently, we observed the clustering of genomes isolated from samples collected in neighbouring countries in the DWV-A group, such as Romania and Moldova (Supplementary Figure S5C). These genomes cluster with genomes isolated in Asian countries (China, South Korea and Japan). We also observed DWV genotype genomes from distant countries within the same cluster, such as DWV-B genomes isolated from samples collected in Germany and Croatia. As observed in the splits graph (Fig. 6), the branches from the common ancestor were shorter for DWV-B than DWV-A. The clustering of DWV variants from the same colony or the same apiary was strengthened by the phylogenetic analysis of partial sequences (Supplementary Figure S6). The analysis of VP1-VP3 coding sequences separated with more accuracy the DWV-A from the DWV-B or DWV recombinants. By analysing the 3 C-protease and RNA dependant RNA polymerase (3Cpro-RdRp) coding sequences, the DWV recombinants were located in both phylogenetic groups (DWV-A and DWV-B). In addition, these partial genome sequence analyses established the phylogenetic relationship between the parental and the recombinant DWV variants detected in the same colony or in the same honey bee head.

## Discussion

In this study, we compared the distribution of the deformed wing virus (DWV) genotypes A and B, and the viral load levels quantified by specific real-time PCR by analysing samples (*n* = 308) collected between 2010 and 2017 from 116 colonies in 15 European countries. The latest genotype to emerge, DWV-B, was reported for the first time in *V. destructor* mites collected in 2001 from the Netherlands^9^, but has already become widespread in Europe some 20 years later (74% of the tested honey bee pools). Except in the samples from Austria (collected from five colonies of the same apiary in 2010), DWV-B was detected in all the sampled countries, suggesting its rapid spread in Europe. Our results are supported by previous studies that have reported a higher prevalence of DWV-B in the United Kingdom^31,47^, Germany^27,31,40^, France^24,48^ and Lithuania^49^. Nevertheless, differences between countries were observed when comparing the viral loads of both DWV genotypes. DWV-B was quantified at higher loads than DWV-A in samples from France, the United Kingdom, Spain, Germany and Croatia. DWV-A was detected in 64% of the pooled samples. Based on viral quantification, we could not exclude that a better-adapted DWV-A still infects honey bees from Italy, Romania, Moldova, Bulgaria, North Macedonia and Austria. This could also suggest that DWV-A was dominant in these countries, because DWV-B might have been introduced only shortly before our study was conducted^50^. For example, DWV-A was dominant in 2010–2011 in North Macedonia whereas DWV-B became dominant in 2017, although the apiaries used in this comparison were different (Fig. 2B, Supplementary Figure S1 and Supplementary Table S1). Recently, Paxton et al. (2022) suggested a switch in the prevalence of DWV-B over DWV-A in Italian honey bees between 2011 and 2019 ^40^. Considering the DWV recombinants previously described in Europe and in the US, which contain in their genome the DWV-B VP3 coding sequence^22,24,44,50–52^, we expected that most of the DWV recombinants would be detected by our DWV-B specific qPCR. This inclusivity of qPCR for DWV-B or DWV recombinants was supported by our sequence analysis of the DWV genotypes. Even when new DWV recombinants were characterised, all of them contained the DWV-B VP1-VP3 coding sequence (Fig. 5 and Supplementary Figure S4). Therefore, the spatial distribution of the DWV genotypes observed in our study was the result of progressive replacement of DWV-A by DWV-B and/or DWV recombinants. This improved efficiency of DWV-B replication in bees was recently demonstrated in a study using infectious plasmids of both DWV genotypes^44^, which also revealed that co-infection of honey bees by both DWV genotypes leads to the production of DWV recombinants.

Ten pools of honey bees from colonies established in three different *V. destructor*-free areas were sequenced. DWV-A was detected at an extremely low level (2.94 log_10_ copies per bee) in one pool from a colony on Ushant Island. Previous studies report the finding of DWV genotypes prior to the arrival of *V. destructor* at low virus loads and with low colony-level prevalence in Hawaii, New Zealand and Newfoundland^10,12,53^. However, a recent study on Australian honey bees reported the absence of DWV in the absence of the mite^54^. One explanation for these differences might be the importation of DWV-infected queens and the horizontal or vertical transmission of the virus without any involvement of *V. destructor* mites^6,55^. Unfortunately, the recent introduction of the *V. destructor* mite on Ushant Island^56^ and in Australia may have changed the DWV loads and diversity.

Analysis of DWV genotype distribution in 105 pools (of whole bees or bee heads) from varroa-infested areas revealed that only DWV-A was detected in 13% of samples (*n* = 14) and only DWV-B was detected in 24% of samples (*n* = 25). However, we did not find any statistically significant differences in either proportion (chi-square test, *p* = 0.05). Ryabov et al. (2017) previously found that colonies from the US with detectable DWV-B and undetectable DWV-A were significantly under-represented^41^. This discrepancy could be due to the target sequence of the primers used by Ryabov, et al.^41^ (non-structural protein coding sequence: 8,645 to 8,780 base on DWV genome, AY292384) being different from that of the primers in our study (VP3 coding sequence: 4,245 to 4,296 base on DWV genome, AY292384). Since the genomic structure of the previously described viral recombinants contained DWV-A non-structural protein domains and DWV-B structural protein domains^22,24^, we considered that recombinants were detected by the DWV-A qPCR in the Ryabov, et al.^41^ study and by the DWV-B qPCR in our study. Therefore, DWV recombinants may modify the distribution of DWV-A and DWV-B viral loads according to the targeted genome sequence. Considering that the 5’NC and structural protein-coding sequences from DWV-B could be associated with the ability to multiply in the *V. destructor* mite^38^, it would make sense to assume that these recombinants originate from DWV-B rather than DWV-A. The assembly of sequencing reads obtained from a single bee head is much simpler than that of reads from pools of whole bees for reconstructing DWV genomes. The pools contained DWV sequence mixtures that were difficult to identify using our pipeline (Supplementary Figure S8). Nevertheless, we detected co-infections by different DWV genotypes and recombinant DWV even in bee heads. However, we detected the presence of a recombinant virus without association with a DWV-A or a DWV-B by qPCR and sequencing from two bee heads (FR-Caumont6_2017_H78 and H111; Supplementary Table S1 and Supplementary Figure S4). In addition to previous observations made under experimental conditions showing that recombinant DWVs could overcome DWV-A^57^, our results from natural infections suggested the ability of some DWV recombinants to spread from bee to bee independently of DWV-A or DWV-B.

Our RT-qPCR data showed that even if one DWV genotype was not detected in the pool, it was detected at a low level in the head of honey bees from the same colony and even in *V. destructor* mites (Fig. 3 and Supplementary Table S1). This suggests that the sensitivity of quantification for individual samples (honey bee head or *V. destructor* mite) was higher than for the pool made from whole honey bees. This conclusion is also supported by the viral loads quantified in several heads, that were below the limit of detection we had previously reported for DWV-A and DWV-B in pools (3.4 and 5.0 log_10_ copies/honey bee, respectively^43^). Indeed, the matrix complexity and the abundance of bee contaminants are higher in the pool of whole honey bees than in single honey bee heads and may reduce the yield of viral RNA extracted or reduce the sensitivity of RNA detection. Nevertheless, the representativeness of the bee sample must also be considered, and at least 60 bees should be collected to detect an intra-colony infection with a prevalence limit of 5% (with 95% of confidence). Thus, testing a pool of honey bee heads may be more useful than testing a pool of whole honey bees for DWV genotype detection in colonies. Therefore, both DWV-A and DWV-B were detected in the head of symptomatic and asymptomatic honey bees as reported previously^20^. The obvious difference between symptomatic and asymptomatic honey bees was the viral load level. In symptomatic honey bees, the viral load was calculated to be above 6.5 log_10_ and 7.7 log_10_ virus/head for DWV-A and DWV-B, respectively. This is consistent with previously reported thresholds separating covert and overt infections, and suggests that a high quantity of viral genome copies is associated with wing deformities, regardless of the DWV genotype^33,43,58^. Therefore, an RT-qPCR method quantifying both DWV genotypes, such as the one recently developed^59^, would not be sufficient to establish which DWV genotype is associated with overt infection. We observed that the relative proportions of DWV-A and DWV-B as well as the viral loads detected in *V. destructor* mites and in honey bee heads from the same colony were correlated (Supplementary Figure S3). Nevertheless, the virus detected in individual mites could have been viruses either accumulated by feeding^36^ or by replicating in mite salivary glands^37^. A deeper analysis of the DWV genotypes present and replicating in the mite would clarify the dynamics of DWV genotype transmission by *V. destructor*.

In conclusion, it was suggested that the *V. destructor* mite was modifying the prevalence of DWV genotypes by vectorising better-adapted viruses (genotype B and DWV recombinants). Our study provides new sequences of DWV-A, DWV-B and DWV recombinants detected in honey bees from 15 European countries exposed to the mite. New hot points for genomic recombination were identified and support the plasticity of the viral genome. All DWV genotypes could be detected in the heads of honey bees, whether they had deformed or normal wings. The main characteristic distinguishing DWV-A from DWV-B and DWV recombinants was the viral loads. The highest levels of DWV-B or DWV recombinants detected in honey bees and *V. destructor* are a robust characteristic reported by several studies. Our study did not allow us to conclude on the date of emergence of DWV-B and associated DWV recombinants. However, it suggests that the emerging DWV-B genotype and related DWV recombinants are continuing to spread in Europe and might replace the historical DWV-A genotype.

## Methods

### Sample collection

In 2017, we collected samples from 116 colonies across 15 European countries (Fig. 1). Complementary samples from viral collections were provided as purified RNA from pools of 10 honey bee heads (44 colonies) or as whole adult honey bees (72 colonies). The detection of DWV in total head RNA was suggested to be a molecular marker for overt DWV infection^20,60,61^. Samples were received frozen (in dry ice) in our laboratory and were kept at -80 °C before analysis. Details of the samples used in this study are listed in Supplementary Table S6.

### Viral genomic RNA preparation

To analyse pooled samples, seven to 20 whole honey bees were crushed in 0.01 mM phosphate buffer (PB, 1 mL per bee) with 5- to 8-mm tungsten beads using a Mixer Mill MM 400 (RETSCH) for 3 min at 30 Hz. Bee homogenates were then cleared by two successive centrifugations at 8,000 x *g* for 10 min at 4 °C. Two millilitres of supernatant were used to perform polyethylene glycol precipitation of macromolecules (10% PEG 8,000 and 300 mM NaCl, final concentrations) after incubation for 2 h at 4 °C. Viruses were recovered by centrifugation at 10,000 x *g* for 30 min at 4 °C. Each pellet was suspended in 400 µL of phosphate buffer (PB). To analyse single bee heads, each head was crushed in PB (500 µL per bee head) with three 3-mm tungsten beads using a Mixer Mill MM 400 (RETSCH) for 1 min at 30 Hz. The homogenate was cleared by two successive centrifugations at 8,000 x *g* for 10 min at 4 °C.

Unprotected nucleic acids co-purified with viruses from the pools of whole bees or bee heads were cleaved in 1X DNase reaction buffer (Invitrogen, AM2238) by the Turbo nuclease (25 U/ml, Sigma-Aldrich T4330) at 37 °C for 2 h.

Individual *V. destructors* were crushed in PB (500 µL per mite) with 1 mL Ten Broeck homogeniser. The homogenates were incubated on ice for 10 min before viral RNA extraction.

RNA was extracted from 140 µL of viral suspension with the QIAamp Viral RNA Mini Kit (Qiagen), without RNA carrier, according to the manufacturer’s instructions. Purified RNA from bee pool or bee head was recovered in 60 µL of elution buffer (80 µl for purified RNA from *V. destructor* mite).

### RT-qPCR analysis

Retro-transcription into cDNA was performed using random primers and the SuperScript II reverse transcriptase (SSRT) kit (Invitrogen) from 12.5 µL of purified RNA according to a previous report (final volume 20 µl)^62^. DWV-A and DWV-B were quantified (using 5 µL of complementary DNA) by two genotype-specific real-time PCRs (qPCR) amplifying the VP3-coding sequences (final volume 25 µl; Supplementary Table S6)^43^. Before log_10_-transformation, the RT-qPCR results were converted into copies per bee, bee head or *V. destructor* mite taking into account the volume of homogenate used for RNA purification, the volume of the elution buffer used for RNA recovery from the spin column, the volume of purified RNA used for cDNA synthesis and the volume of cDNA tested by qPCR. The final quantities were calculated by multiplying the instrument’s measurement with the dilution factor: 27.4 for pool load, 68.5 for bee head load or 91.4 for mite load. The quantification method’s accuracy was also analysed (Supplementary Figure S7^63^). Both RT-qPCR methods quantified the recombinant plasmids within the acceptability limits, i.e. ±1.0 log_10_ copies per whole bee or per bee head.

### Statistical analysis

The number of DWV-A or DWV-B log_10_ copies estimated from the heads of honey bees either with or without deformed wings was compared using the s Mann-Withney U test (used for comparing not normally distributed viral loads of equal or different sample sizes). The relationship between the viral loads in bees and of *V. destructor* infestation level, were tested with Spearman’s test for small numbers of data points and non-normal distributions. The DWV-A and DWV-B prevalences were compared by a chi-square test. The *p*-values were calculated using R package lawstat (v3.2). Differences were considered significant at *p* < 0.05.

### RNA sequencing and data pre-processing

The NEBNext rRNA depletion kit (New England Biolabs, E6310) and NEBNext Ultra II directional RNA kit (New England Biolabs, E7760) were used to prepare the sequencing libraries using 12 µL of RNA eluate. The libraries were subject to quality control using a Tapestation HS D5000 (Agilent). Qubit (Invitrogen) was used for quantification. Libraries were pooled and sequenced with a Nextseq 500 (Illumina) using the 150-cycle high output kit at the sequencing core facility of the Brain & Spine Institute, Paris, France.

Sequencing reads were cleaned with Trimmomatic (Bolger et al., 2014, v0.36) applying parameter settings (ILLUMINACLIP: oligos.fasta: 2:30:5:1: true; LEADING: 3; TRAILING: 3; MAXINFO: 40:0.2; MINLEN: 36).

### Detection of honey bee viruses

Cleaned reads were aligned with 13 viral honey bee reference genomes (Supplementary Table S7) using Megablast^64^. Reads aligning more than once were removed from the output files. When more than 90% of a given viral genome was covered in a given sample, the reads per kilobase million (RPKM) was calculated using viral genome length and total number of cleaned reads produced as normalisation values. The results were visualised using pheatmap in R (v1.0.12).

### DWV genotype genomic structure

For recombinant junction identification, cleaned reads were aligned simultaneously with the most relevant DWV-A and DWV-B parental genomes using Speedseq (v0.1.2)^65^ (Supplementary Table S8). Then LUMPY (v0.2.13)-smoove (v0.2.5) was used to identify the recombination junctions from discordant and split reads^66^. The number of sampled reads used to estimate mean and standard deviation of insert size was adjusted to optimise the analysis run time. Outputs from LUMPY were processed with SVtyper (v0.7.0) to obtain additional structural genotype statistics^65^. Potential recombinant junctions were defined from break end events with over ten supporting reads (SU), over 1,000 X coverage depth (DP) and over 0.05 allele balance (AB; fraction of observations from alternate allele). A graphical representation of genome coverage depth and the position of complete LUMPY-defined break end events for each sample was generated with the ggplot2 (v3.1) package in R. Graphical representations of all LUMPY-defined junctions were generated using the trackViewer (v1.18) package in R.

### *De novo* assembly and genome reconstruction of DWV genotypes

Honey bee associated reads were removed after mapping to the Amel 4.5 reference genome (GCA_000002195.1). Reads were mapped to the most homologous DWV-A or DWV-B genotype reference genome (Supplementary Table S8) to estimate coverage depth. Average coverage depth was used to calculate the number of reads to sample in order to get an average coverage depth of 80X. Subsample reads were *de novo* assembled using SPAdes (v3.10.0) into contigs and aligned to the reference genome to generate a consensus sequence^67^. This subsampling strategy was used to optimise the run time of *de novo* assembly and facilitate assembly of the main genotype. The alignment of consensus and contigs against the reference genome were visualised with Integrative Genomics Viewer (IGV, v2.3.91)^68^. The assembly of a full-length sequence was achieved by using overlapping junctions. The graphical representations containing the coverage depth and LUMPY- and ViReMa^69^-defined junctions were used to guide genome reconstruction specifically for recombinant genomes (Supplementary Figure S8). In the case of a mix of DWV-A or DWV-B and DWV recombinant, assembly of the recombinant genome was achieved by joining the contigs of the main background genome (DWV-A or DWV-B) with remaining contigs. The reconstructed genome were validated by alignment of the reads with BWA-MEME (v0.7.17) on the proposed genome^70^. The putative junctions were particularly scrutinized and must display a perfect alignment of the reads on the reconstructed genome to be ascertained. Finally, the open reading frames of each reconstructed genome were validated by comparing them with the known start and end positions of the reference genomes.

### Phylogenetic analysis

Whole-genome nucleic acid sequences of reconstructed and reference genomes (Supplementary Table S9) were aligned using MAFFT (v7.221.3) in automatic mode with Galaxy. The alignment block was edited with MEGA (v10.0.5) by removing nucleotides at the start (110 nucleotides) and end (65 nucleotides). The alignment length was 10,066 nucleotides. The splits graph network was generated with Splitstree 4 using multiple sequence alignments. The network was constructed using uncorrected *p* distances transformed using the neighbour net method. For the phylogenetic tree, best-fit models of nucleotide substitutions were statistically selected using JmodelTest (v2.1.10) with default settings (ML optimised base tree and Best base tree search). GTR + G + I was selected as the best-fit substitution model according to Akaike criteria. Maximum likelihood trees were constructed using raxmlGUI v1.5 (RAxML v8.1.2) without outgroups using the ML + rapid bootstrap analysis type, with the number of bootstrap replicates set to autoMRE. The bootstrap convergence test defines 600 replicates as sufficient to obtain stable support values. The RaxML partition tree was edited with Dendroscope (v3.5.10).

Partial coding sequences (VP1-VP3 and 3Cpro-RdRp coding sequences, nucleotide position 2594 to 4616 and 7679 to 9821 on the DWV genome Y292384, respectively) were extracted from each of the 107 assembled consensus and 26 reference DWV genome sequences using BLAST. The Multiple Sequence Alignment Software MAFF was used in auto mode with default parameters to align the VP1-VP3 or 3 C-RdRp sequences^71^. Each alignment was trimmed with TrimAl using nogap mode^72^. The trimmed alignment Fasta file was loaded in the PhyML 3.1/3.0 aLRT from the Phylogeny.fr platform using aLRT statistical test and GTR substitution model^73^. The phylogenetic tree was then edited with TreeDyn 198.3 ^73^.

## Electronic supplementary material

Below is the link to the electronic supplementary material.

Supplementary Material 1

## Data Availability

All the NGS data files used in this paper are publicly available in the NCBI’s short read archive (SRA), which is accessible under BioProject ID: PRJNA1055031.
