# Supplementary material for: Genotype B of deformed wing virus and related recombinant viruses become dominant in European honey bee colonies
Source: Sci Rep. 2025 Feb 8;15:4804. doi: 10.1038/s41598-025-86937-5 (PMC11807101; doi:10.1038/s41598-025-86937-5)
Supplement: Supplementary file 1 — Supplementary Material 1 [file 41598_2025_86937_MOESM1_ESM.docx]

**Supplementary Information**

**Genotype B of deformed wing virus and related recombinant viruses become dominant in European honey bee colonies**

**Authors:**

Fabrice Sircoulomb^1^, Eric Dubois^1^, Frank Schurr^1^, Pierrick Lucas^2^, Marina Meixner^3^, Alicia Bertolotti^1^, Yannick Blanchard^2^ and Richard Thiéry^1^

**Affiliations:**

^1^ANSES Sophia Antipolis laboratory, Unit of Honey Bee Pathology, Sophia Antipolis, France

^2^ANSES Ploufragan – Plouzané – Niort laboratory, Unit of Viral Genetic and Biosecurity, Ploufragan, France

^3^LLH Bee Institute Kirchhain, 35274 Kirchhain, Germany

**This PDF file includes:**

- Supplementary Figure S1,
- Supplementary Table S1,
- Supplementary Figure S2,
- Supplementary Figure S3,
- Supplementary Figure S4,
- Supplementary Table S2,
- Supplementary Table S3,
- Supplementary Table S4,
- Supplementary Figure S5,
- Supplementary Figure S6,
- Supplementary Table S5,
- Supplementary Figure S7,
- Supplementary Table S6,
- Supplementary Table S7,
- Supplementary Table S8,
- Supplementary Figure S8,
- Supplementary Table S9.

**
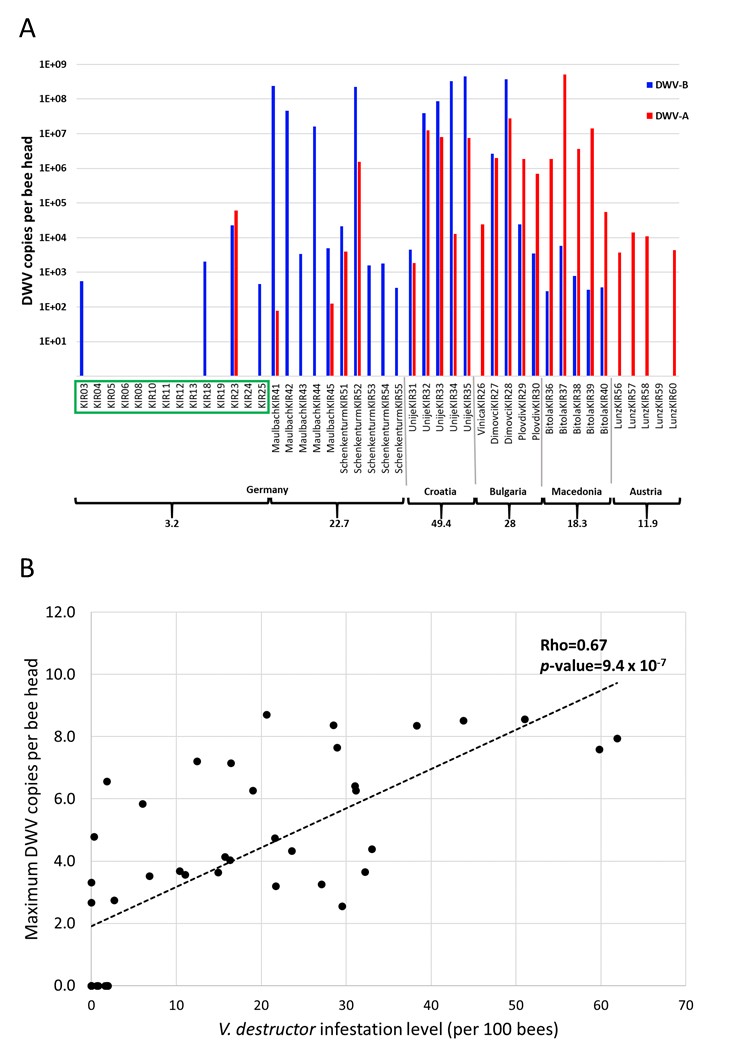
**

**Supplementary Figure S1.** DWV-A and DWV-B loads quantified in RNA samples (44 colonies). **A**: Viral loads of DWV-A and DWV-B according to the country of origin. The red and blue bars represent the DWV-A and DWV-B loads, respectively. Colonies treated against *V. destructor* (oxalic acid) are contained within the green box. The numbers at the bottom correspond to the mean mite infestation level (number of *V. destructor* per 100 bees) for a given group of samples. Viral loads are expressed in equivalent of viral genome copies per bee head (copies per bee head). **B**: Relationship between mite infestation level and the maximum observed viral load (log_10_ copies per bee head) from DWV-A or DWV-B. The dashed line corresponds to the trend line. The Spearman Rho and p-value are shown in the figure.

| **Supplementary Table S1.** DWV-A and DWV-B loads quantified by RT-qPCR in pools of honey bee heads (retrieved as RNA samples), pools of whole honey bees, single head of honey bees or in *Varroa destructor* samples collected in European honey bee colonies.   \| **Sample identification ^1^** \| **DWV-A copies  per bee, bee head  or mite (log_10_) ^2^** \| **DWV-B copies  per bee, bee head  or mite (log_10_) ^3^** \| **Matrice** \| **Wing  morphology ^4^** \| \| --- \| --- \| --- \| --- \| --- \| \| AT-LunzKIR56_2010_P \| 3.6 \| 0.0 \| pool (bee heads) \| NA \| \| AT-LunzKIR57_2010_P \| 4.1 \| 0.0 \| pool (bee heads) \| NA \| \| AT-LunzKIR58_2010_P \| 4.0 \| 0.0 \| pool (bee heads) \| NA \| \| AT-LunzKIR59_2010_P \| 0.0 \| 0.0 \| pool (bee heads) \| NA \| \| AT-LunzKIR60_2010_P \| 3.6 \| 0.0 \| pool (bee heads) \| NA \| \| BG-DimovciKIR27_2011_P \| 6.3 \| 6.4 \| pool (bee heads) \| NA \| \| BG-DimovciKIR28_2011_P \| 7.4 \| 8.6 \| pool (bee heads) \| NA \| \| BG-PlovdivKIR29_2011_P \| 6.3 \| 4.4 \| pool (bee heads) \| NA \| \| BG-PlovdivKIR30_2011_P \| 5.8 \| 3.5 \| pool (bee heads) \| NA \| \| BG-VinicaKIR26_2011_P \| 4.4 \| 0.0 \| pool (bee heads) \| NA \| \| DE-KIR03_2010_P \| 0.0 \| 2.7 \| pool (bee heads) \| NA \| \| DE-KIR04_2010_P \| 0.0 \| 0.0 \| pool (bee heads) \| NA \| \| DE-KIR05_2010_P \| 0.0 \| 0.0 \| pool (bee heads) \| NA \| \| DE-KIR06_2010_P \| 0.0 \| 0.0 \| pool (bee heads) \| NA \| \| DE-KIR08_2010_P \| 0.0 \| 0.0 \| pool (bee heads) \| NA \| \| DE-KIR10_2010_P \| 0.0 \| 0.0 \| pool (bee heads) \| NA \| \| DE-KIR11_2010_P \| 0.0 \| 0.0 \| pool (bee heads) \| NA \| \| DE-KIR12_2010_P \| 0.0 \| 0.0 \| pool (bee heads) \| NA \| \| DE-KIR13_2010_P \| 0.0 \| 0.0 \| pool (bee heads) \| NA \| \| DE-KIR18_2010_P \| 0.0 \| 3.3 \| pool (bee heads) \| NA \| \| DE-KIR19_2010_P \| 0.0 \| 0.0 \| pool (bee heads) \| NA \| \| DE-KIR23_2010_P \| 4.8 \| 4.4 \| pool (bee heads) \| NA \| \| DE-KIR24_2010_P \| 0.0 \| 0.0 \| pool (bee heads) \| NA \| \| DE-KIR25_2010_P \| 0.0 \| 2.7 \| pool (bee heads) \| NA \| \| DE-MaulbachKIR41_2010_P \| 1.9 \| 8.4 \| pool (bee heads) \| NA \| \| DE-MaulbachKIR42_2010_P \| 0.0 \| 7.7 \| pool (bee heads) \| NA \| \| DE-MaulbachKIR43_2010_P \| 0.0 \| 3.5 \| pool (bee heads) \| NA \| \| DE-MaulbachKIR44_2010_P \| 0.0 \| 7.2 \| pool (bee heads) \| NA \| \| DE-MaulbachKIR45_2010_P \| 2.1 \| 3.7 \| pool (bee heads) \| NA \| \| DE-SchenkenturmKIR51_2010_P \| 3.6 \| 4.3 \| pool (bee heads) \| NA \| \| DE-SchenkenturmKIR52_2010_P \| 6.2 \| 8.4 \| pool (bee heads) \| NA \| \| DE-SchenkenturmKIR53_2010_P \| 0.0 \| 3.2 \| pool (bee heads) \| NA \| \| DE-SchenkenturmKIR54_2010_P \| 0.0 \| 3.3 \| pool (bee heads) \| NA \| \| DE-SchenkenturmKIR55_2010_P \| 0.0 \| 2.6 \| pool (bee heads) \| NA \| \| HR-UnijeKIR31_2011_P \| 3.3 \| 3.6 \| pool (bee heads) \| NA \| \| HR-UnijeKIR32_2011_P \| 7.1 \| 7.6 \| pool (bee heads) \| NA \| \| HR-UnijeKIR33_2011_P \| 6.9 \| 7.9 \| pool (bee heads) \| NA \| \| HR-UnijeKIR34_2011_P \| 4.1 \| 8.5 \| pool (bee heads) \| NA \| \| HR-UnijeKIR35_2011_P \| 6.9 \| 8.7 \| pool (bee heads) \| NA \| \| MK-BitolaKIR36_2011_P \| 6.3 \| 2.5 \| pool (bee heads) \| NA \| \| MK-BitolaKIR37_2010_P \| 8.7 \| 3.8 \| pool (bee heads) \| NA \| \| MK-BitolaKIR38_2011_P \| 6.6 \| 2.9 \| pool (bee heads) \| NA \| \| MK-BitolaKIR39_2011_P \| 7.2 \| 2.5 \| pool (bee heads) \| NA \| \| MK-BitolaKIR40_2011_P \| 4.7 \| 2.6 \| pool (bee heads) \| NA \| \| ES-Arbeiza21_2017_P \| 8.7 \| 9.1 \| pool (whole honeybees) \| NA \| \| ES-Arbeiza22_2017_P \| 0.0 \| 9.8 \| pool (whole honeybees) \| NA \| \| ES-Fraisoro17_2017_P \| 3.2 \| 9.7 \| pool (whole honeybees) \| NA \| \| ES-Fraisoro18_2017_P \| 0.0 \| 7.4 \| pool (whole honeybees) \| NA \| \| ES-Opakua(Araba)19_2017_P \| 8.5 \| 11.5 \| pool (whole honeybees) \| NA \| \| ES-Opakua(Araba)20_2017_P \| 4.1 \| 9.9 \| pool (whole honeybees) \| NA \| \| FI-Åland69_2016_P \| 0.0 \| 0.0 \| pool (whole honeybees) \| NA \| \| FI-Åland70_2016_P \| 0.0 \| 0.0 \| pool (whole honeybees) \| NA \| \| FI-Åland71_2016_P \| 0.0 \| 0.0 \| pool (whole honeybees) \| NA \| \| FR-Caumont10_2017_P \| 0.0 \| 9.5 \| pool (whole honeybees) \| NA \| \| FR-Caumont6_2017_P \| 0.0 \| 10.5 \| pool (whole honeybees) \| NA \| \| FR-Caumont7_2017_P \| 0.0 \| 8.5 \| pool (whole honeybees) \| NA \| \| FR-Caumont8_2017_P \| 0.0 \| 10.6 \| pool (whole honeybees) \| NA \| \| FR-Caumont9_2017_P \| 0.0 \| 10.9 \| pool (whole honeybees) \| NA \| \| FR-Ouessant55_2011_P \| 0.0 \| 0.0 \| pool (whole honeybees) \| NA \| \| FR-Ouessant56_2011_P \| 0.0 \| 0.0 \| pool (whole honeybees) \| NA \| \| FR-Ouessant57_2011_P \| 2.9 \| 0.0 \| pool (whole honeybees) \| NA \| \| FR-Ouessant58_2011_P \| 0.0 \| 0.0 \| pool (whole honeybees) \| NA \| \| FR-Ouessant61_2011_P \| 0.0 \| 0.0 \| pool (whole honeybees) \| NA \| \| FR-Ouessant62_2011_P \| 0.0 \| 0.0 \| pool (whole honeybees) \| NA \| \| FR-Sophia1_2017_P \| 0.0 \| 10.3 \| pool (whole honeybees) \| NA \| \| FR-Sophia2_2017_P \| 0.0 \| 8.5 \| pool (whole honeybees) \| NA \| \| FR-Sophia3_2017_P \| 0.0 \| 10.1 \| pool (whole honeybees) \| NA \| \| GR-DomenicoLavisa11_2017_P \| 3.5 \| 11.0 \| pool (whole honeybees) \| NA \| \| GR-DomenicoLavisa12_2017_P \| 0.0 \| 7.6 \| pool (whole honeybees) \| NA \| \| GR-MoudaniaChalkidiki13_2017_P \| 3.5 \| 6.0 \| pool (whole honeybees) \| NA \| \| GR-MoudaniaChalkidiki14_2017_P \| 0.0 \| 0.0 \| pool (whole honeybees) \| NA \| \| HR-HrvatskaKostajnica32_2017_P \| 0.0 \| 9.5 \| pool (whole honeybees) \| NA \| \| HR-HrvatskaKostajnica50_2017_P \| 4.3 \| 5.8 \| pool (whole honeybees) \| NA \| \| HR-Sisak39_2017_P \| 9.4 \| 9.9 \| pool (whole honeybees) \| NA \| \| HR-Sisak47_2017_P \| 7.1 \| 11.2 \| pool (whole honeybees) \| NA \| \| HR-Sisak48_2017_P \| 7.9 \| 5.5 \| pool (whole honeybees) \| NA \| \| HR-Sisak49_2017_P \| 4.2 \| 8.0 \| pool (whole honeybees) \| NA \| \| IT-Napoli59_2017_P \| 7.4 \| 0.0 \| pool (whole honeybees) \| NA \| \| IT-Napoli60_2017_P \| 7.5 \| 0.0 \| pool (whole honeybees) \| NA \| \| IT-Palermo4_2017_P \| 9.6 \| 8.5 \| pool (whole honeybees) \| NA \| \| IT-Palermo5_2017_P \| 9.9 \| 7.1 \| pool (whole honeybees) \| NA \| \| IT-Palermo51_2017_P \| 10.4 \| 9.0 \| pool (whole honeybees) \| NA \| \| IT-Palermo52_2017_P \| 10.5 \| 9.9 \| pool (whole honeybees) \| NA \| \| MD-GhidighiciChisinau23_2017_P \| 8.8 \| 0.0 \| pool (whole honeybees) \| NA \| \| MD-GhidighiciChisinau24_2017_P \| 9.7 \| 6.3 \| pool (whole honeybees) \| NA \| \| MD-GhidighiciChisinau25_2017_P \| 9.4 \| 0.0 \| pool (whole honeybees) \| NA \| \| MD-GhidighiciChisinau26_2017_P \| 9.7 \| 7.3 \| pool (whole honeybees) \| NA \| \| MK-MalinoSvetiNikole15_2017_P \| 7.5 \| 9.9 \| pool (whole honeybees) \| NA \| \| MK-MalinoSvetiNikole16_2017_P \| 9.5 \| 10.1 \| pool (whole honeybees) \| NA \| \| MK-SDihovoBitola53_2017_P \| 5.3 \| 9.0 \| pool (whole honeybees) \| NA \| \| MK-SDihovoBitola54_2017_P \| 4.9 \| 0.0 \| pool (whole honeybees) \| NA \| \| NO-Buskerud64_2012_P \| 8.0 \| 0.0 \| pool (whole honeybees) \| NA \| \| NO-Buskerud66_2014_P \| 7.6 \| 8.4 \| pool (whole honeybees) \| NA \| \| NO-Østfold65_2014_P \| 8.0 \| 8.7 \| pool (whole honeybees) \| NA \| \| NO-Rogaland67_2014_P \| 7.9 \| 8.2 \| pool (whole honeybees) \| NA \| \| NO-Vestfold63_2012_P \| 8.0 \| 0.0 \| pool (whole honeybees) \| NA \| \| RO-Bucharest40_2017_P \| 10.0 \| 6.2 \| pool (whole honeybees) \| NA \| \| RO-Bucharest41_2017_P \| 10.0 \| 5.4 \| pool (whole honeybees) \| NA \| \| RO-Bucharest42_2017_P \| 5.3 \| 0.0 \| pool (whole honeybees) \| NA \| \| RO-Otopeni45_2017_P \| 7.0 \| 6.5 \| pool (whole honeybees) \| NA \| \| RO-Otopeni46_2017_P \| 6.0 \| 6.1 \| pool (whole honeybees) \| NA \| \| RO-TarguMures31_2017_P \| 8.0 \| 8.5 \| pool (whole honeybees) \| NA \| \| RS-Belgrade29_2017_P \| 6.0 \| 7.0 \| pool (whole honeybees) \| NA \| \| RS-Belgrade30_2017_P \| 9.0 \| 0.0 \| pool (whole honeybees) \| NA \| \| RS-Kratjevo27_2017_P \| 3.3 \| 6.5 \| pool (whole honeybees) \| NA \| \| RS-Kratjevo28_2017_P \| 3.8 \| 5.9 \| pool (whole honeybees) \| NA \| \| RS-YrsacUlima33_2017_P \| 6.9 \| 6.3 \| pool (whole honeybees) \| NA \| \| RS-YrsacUlima34_2017_P \| 0.0 \| 0.0 \| pool (whole honeybees) \| NA \| \| UK-Colonsay72_2017_P \| 0.0 \| 0.0 \| pool (whole honeybees) \| NA \| \| UK-Flaxton37_2017_P \| 0.0 \| 11.5 \| pool (whole honeybees) \| NA \| \| UK-Flaxton38_2017_P \| 0.0 \| 11.3 \| pool (whole honeybees) \| NA \| \| UK-SandHutton43_2017_P \| 9.0 \| 11.9 \| pool (whole honeybees) \| NA \| \| UK-SandHutton44_2017_P \| 5.5 \| 9.4 \| pool (whole honeybees) \| NA \| \| UK-SherburnInElmet35_2017_P \| 0.0 \| 11.0 \| pool (whole honeybees) \| NA \| \| UK-SherburnInElmet36_2017_P \| 0.0 \| 10.8 \| pool (whole honeybees) \| NA \| \| DE-HohenNeuendorf_2015_H1 \| 0.0 \| 9.4 \| single head \| deformed \| \| DE-HohenNeuendorf_2015_H10 \| 0.0 \| 9.7 \| single head \| deformed \| \| DE-HohenNeuendorf_2015_H2 \| 0.0 \| 10.2 \| single head \| deformed \| \| DE-HohenNeuendorf_2015_H3 \| 0.0 \| 9.5 \| single head \| deformed \| \| DE-HohenNeuendorf_2015_H4 \| 0.0 \| 10.1 \| single head \| deformed \| \| DE-HohenNeuendorf_2015_H5 \| 0.0 \| 9.0 \| single head \| deformed \| \| DE-HohenNeuendorf_2015_H6 \| 0.0 \| 10.6 \| single head \| deformed \| \| DE-HohenNeuendorf_2015_H7 \| 0.0 \| 10.4 \| single head \| deformed \| \| DE-HohenNeuendorf_2015_H8 \| 0.0 \| 10.4 \| single head \| deformed \| \| DE-HohenNeuendorf_2015_H9 \| 0.0 \| 10.5 \| single head \| deformed \| \| ES-Arbeiza21_2017_H177 \| 2.8 \| 9.6 \| single head \| deformed \| \| ES-Arbeiza21_2017_H178 \| 3.3 \| 6.4 \| single head \| deformed \| \| ES-Arbeiza21_2017_H179 \| 2.7 \| 9.6 \| single head \| deformed \| \| ES-Arbeiza21_2017_H180 \| 4.9 \| 0.0 \| single head \| deformed \| \| ES-Arbeiza21_2017_H181 \| 2.7 \| 0.0 \| single head \| deformed \| \| ES-Arbeiza21_2017_H182 \| 2.7 \| 9.6 \| single head \| deformed \| \| ES-Arbeiza21_2017_H183 \| 3.9 \| 9.5 \| single head \| deformed \| \| ES-Arbeiza21_2017_H184 \| 2.1 \| 9.6 \| single head \| deformed \| \| ES-Arbeiza21_2017_H185 \| 2.2 \| 9.5 \| single head \| deformed \| \| ES-Arbeiza22_2017_H31 \| 5.0 \| 10.2 \| single head \| deformed \| \| ES-Arbeiza22_2017_H32 \| 4.4 \| 11.0 \| single head \| deformed \| \| ES-Arbeiza22_2017_H33 \| 4.1 \| 10.7 \| single head \| deformed \| \| ES-Arbeiza22_2017_H34 \| 5.4 \| 10.7 \| single head \| deformed \| \| ES-Arbeiza22_2017_H35 \| 10.1 \| 10.2 \| single head \| deformed \| \| ES-Arbeiza22_2017_H36 \| 5.6 \| 10.1 \| single head \| deformed \| \| ES-Arbeiza22_2017_H37 \| 10.0 \| 10.1 \| single head \| deformed \| \| ES-Arbeiza22_2017_H38 \| 7.1 \| 10.8 \| single head \| deformed \| \| ES-Arbeiza22_2017_H39 \| 5.5 \| 10.1 \| single head \| deformed \| \| ES-Arbeiza22_2017_H40 \| 5.7 \| 10.4 \| single head \| deformed \| \| ES-Arbeiza22_2017_H61 \| 7.1 \| 0.0 \| single head \| normal \| \| ES-Arbeiza22_2017_H62 \| 0.0 \| 9.6 \| single head \| normal \| \| ES-Arbeiza22_2017_H63 \| 0.0 \| 6.6 \| single head \| normal \| \| ES-Arbeiza22_2017_H64 \| 0.0 \| 6.3 \| single head \| normal \| \| ES-Arbeiza22_2017_H65 \| 0.0 \| 0.0 \| single head \| normal \| \| ES-Arbeiza22_2017_H66 \| 2.5 \| 0.0 \| single head \| normal \| \| ES-Arbeiza22_2017_H67 \| 0.0 \| 7.6 \| single head \| normal \| \| ES-Arbeiza22_2017_H68 \| 0.0 \| 7.2 \| single head \| normal \| \| ES-Arbeiza22_2017_H69 \| 0.0 \| 7.1 \| single head \| normal \| \| ES-Arbeiza22_2017_H70 \| 0.0 \| 6.9 \| single head \| normal \| \| ES-Opakua(Araba)20_2017_H101 \| 3.3 \| 3.2 \| single head \| normal \| \| ES-Opakua(Araba)20_2017_H102 \| 2.6 \| 3.3 \| single head \| normal \| \| ES-Opakua(Araba)20_2017_H103 \| 2.6 \| 4.1 \| single head \| normal \| \| ES-Opakua(Araba)20_2017_H104 \| 2.1 \| 2.2 \| single head \| normal \| \| ES-Opakua(Araba)20_2017_H105 \| 3.8 \| 4.3 \| single head \| normal \| \| ES-Opakua(Araba)20_2017_H106 \| 2.2 \| 3.0 \| single head \| normal \| \| ES-Opakua(Araba)20_2017_H107 \| 0.0 \| 2.2 \| single head \| normal \| \| ES-Opakua(Araba)20_2017_H108 \| 0.0 \| 4.2 \| single head \| normal \| \| ES-Opakua(Araba)20_2017_H109 \| 0.0 \| 1.8 \| single head \| normal \| \| ES-Opakua(Araba)20_2017_H110 \| 0.0 \| 3.4 \| single head \| normal \| \| FR-Caumont6_2017_H111 \| 0.0 \| 9.1 \| single head \| deformed \| \| FR-Caumont6_2017_H112 \| 0.0 \| 9.5 \| single head \| deformed \| \| FR-Caumont6_2017_H113 \| 2.4 \| 9.4 \| single head \| deformed \| \| FR-Caumont6_2017_H114 \| 3.4 \| 7.8 \| single head \| deformed \| \| FR-Caumont6_2017_H115 \| 2.0 \| 9.2 \| single head \| deformed \| \| FR-Caumont6_2017_H116 \| 0.0 \| 8.5 \| single head \| deformed \| \| FR-Caumont6_2017_H71 \| 0.0 \| 0.0 \| single head \| normal \| \| FR-Caumont6_2017_H72 \| 6.2 \| 0.0 \| single head \| normal \| \| FR-Caumont6_2017_H73 \| 3.2 \| 6.7 \| single head \| normal \| \| FR-Caumont6_2017_H74 \| 0.0 \| 0.0 \| single head \| normal \| \| FR-Caumont6_2017_H75 \| 0.0 \| 0.0 \| single head \| normal \| \| FR-Caumont6_2017_H76 \| 0.0 \| 6.7 \| single head \| normal \| \| FR-Caumont6_2017_H77 \| 0.0 \| 0.0 \| single head \| normal \| \| FR-Caumont6_2017_H78 \| 0.0 \| 8.2 \| single head \| normal \| \| FR-Caumont6_2017_H79 \| 0.0 \| 0.0 \| single head \| normal \| \| FR-Caumont6_2017_H80 \| 0.0 \| 0.0 \| single head \| normal \| \| FR-Caumont8_2017_H196 \| 1.8 \| 9.5 \| single head \| deformed \| \| FR-Caumont8_2017_H197 \| 4.1 \| 9.6 \| single head \| deformed \| \| FR-Caumont8_2017_H198 \| 4.0 \| 9.5 \| single head \| deformed \| \| FR-Caumont8_2017_H199 \| 4.3 \| 9.4 \| single head \| deformed \| \| FR-Caumont8_2017_H200 \| 3.4 \| 9.5 \| single head \| deformed \| \| FR-Caumont8_2017_H201 \| 0.0 \| 9.5 \| single head \| deformed \| \| FR-Caumont8_2017_H202 \| 2.1 \| 9.6 \| single head \| deformed \| \| FR-Caumont8_2017_H203 \| 0.0 \| 9.5 \| single head \| deformed \| \| FR-Caumont8_2017_H204 \| 0.0 \| 9.5 \| single head \| deformed \| \| FR-Caumont8_2017_H205 \| 0.0 \| 9.6 \| single head \| deformed \| \| FR-Caumont8_2017_H206 \| 10.7 \| 9.9 \| single head \| normal \| \| FR-Caumont8_2017_H207 \| 0.0 \| 5.6 \| single head \| normal \| \| FR-Caumont8_2017_H208 \| 0.0 \| 5.1 \| single head \| normal \| \| FR-Caumont8_2017_H209 \| 0.0 \| 10.5 \| single head \| normal \| \| FR-Caumont8_2017_H210 \| 0.0 \| 9.5 \| single head \| normal \| \| FR-Caumont8_2017_H211 \| 6.4 \| 7.8 \| single head \| normal \| \| FR-Caumont8_2017_H212 \| 0.0 \| 5.3 \| single head \| normal \| \| FR-Caumont8_2017_H213 \| 8.4 \| 5.0 \| single head \| normal \| \| FR-Caumont8_2017_H214 \| 2.7 \| 5.9 \| single head \| normal \| \| FR-Caumont8_2017_H215 \| 3.9 \| 5.6 \| single head \| normal \| \| FR-Sophia1_2017_H171 \| 0.0 \| 9.5 \| single head \| deformed \| \| FR-Sophia1_2017_H172 \| 2.4 \| 9.6 \| single head \| deformed \| \| FR-Sophia2_2017_H173 \| 3.3 \| 9.6 \| single head \| deformed \| \| FR-Sophia2_2017_H174 \| 4.6 \| 9.6 \| single head \| deformed \| \| FR-Sophia2_2017_H175 \| 3.2 \| 9.6 \| single head \| deformed \| \| FR-Sophia2_2017_H176 \| 2.4 \| 9.6 \| single head \| deformed \| \| HR-HrvatskaKostajnica50_2017_H121 \| 8.9 \| 4.8 \| single head \| deformed \| \| HR-HrvatskaKostajnica50_2017_H122 \| 4.1 \| 9.2 \| single head \| deformed \| \| HR-HrvatskaKostajnica50_2017_H124 \| 4.5 \| 9.1 \| single head \| deformed \| \| HR-HrvatskaKostajnica50_2017_H128 \| 10.1 \| 9.7 \| single head \| deformed \| \| HR-HrvatskaKostajnica50_2017_H81 \| 5.2 \| 6.4 \| single head \| normal \| \| HR-HrvatskaKostajnica50_2017_H82 \| 5.6 \| 6.3 \| single head \| normal \| \| HR-HrvatskaKostajnica50_2017_H83 \| 8.7 \| 7.1 \| single head \| normal \| \| HR-HrvatskaKostajnica50_2017_H84 \| 5.6 \| 6.3 \| single head \| normal \| \| HR-HrvatskaKostajnica50_2017_H85 \| 5.1 \| 5.8 \| single head \| normal \| \| HR-HrvatskaKostajnica50_2017_H86 \| 8.8 \| 5.8 \| single head \| normal \| \| HR-HrvatskaKostajnica50_2017_H87 \| 6.0 \| 5.5 \| single head \| normal \| \| HR-HrvatskaKostajnica50_2017_H88 \| 5.2 \| 9.5 \| single head \| normal \| \| HR-HrvatskaKostajnica50_2017_H89 \| 6.3 \| 6.6 \| single head \| normal \| \| HR-HrvatskaKostajnica50_2017_H90 \| 5.3 \| 6.0 \| single head \| normal \| \| IT-Napoli59_2017_H161 \| 9.3 \| 3.4 \| single head \| deformed \| \| IT-Napoli59_2017_H162 \| 9.4 \| 4.6 \| single head \| deformed \| \| IT-Napoli59_2017_H163 \| 7.4 \| 3.6 \| single head \| deformed \| \| IT-Napoli59_2017_H164 \| 9.4 \| 3.5 \| single head \| deformed \| \| IT-Napoli59_2017_H165 \| 7.4 \| 3.7 \| single head \| deformed \| \| IT-Napoli59_2017_H166 \| 7.8 \| 4.1 \| single head \| deformed \| \| IT-Napoli59_2017_H167 \| 9.5 \| 3.7 \| single head \| deformed \| \| IT-Napoli59_2017_H168 \| 8.4 \| 4.1 \| single head \| deformed \| \| IT-Napoli59_2017_H169 \| 8.4 \| 3.5 \| single head \| deformed \| \| IT-Napoli59_2017_H170 \| 9.5 \| 3.5 \| single head \| deformed \| \| IT-Palermo4_2017_H141 \| 9.6 \| 2.6 \| single head \| deformed \| \| IT-Palermo4_2017_H142 \| 9.9 \| 2.8 \| single head \| deformed \| \| IT-Palermo4_2017_H143 \| 9.8 \| 3.1 \| single head \| deformed \| \| IT-Palermo4_2017_H144 \| 9.1 \| 0.0 \| single head \| deformed \| \| IT-Palermo4_2017_H145 \| 10.4 \| 0.0 \| single head \| deformed \| \| MD-GhidighiciChisinau23_2017_H151 \| 8.1 \| 2.1 \| single head \| deformed \| \| MD-GhidighiciChisinau23_2017_H152 \| 9.2 \| 3.6 \| single head \| deformed \| \| MD-GhidighiciChisinau23_2017_H153 \| 9.2 \| 4.3 \| single head \| deformed \| \| MD-GhidighiciChisinau23_2017_H154 \| 9.4 \| 3.7 \| single head \| deformed \| \| MD-GhidighiciChisinau23_2017_H155 \| 9.4 \| 2.3 \| single head \| deformed \| \| MD-GhidighiciChisinau23_2017_H156 \| 9.4 \| 0.0 \| single head \| deformed \| \| MD-GhidighiciChisinau23_2017_H157 \| 9.4 \| 2.4 \| single head \| deformed \| \| MD-GhidighiciChisinau23_2017_H158 \| 9.4 \| 2.2 \| single head \| deformed \| \| MD-GhidighiciChisinau23_2017_H159 \| 9.2 \| 0.0 \| single head \| deformed \| \| MD-GhidighiciChisinau23_2017_H160 \| 9.3 \| 1.9 \| single head \| deformed \| \| MK-MalinoSvetiNikole15_2017_H11 \| 6.0 \| 10.7 \| single head \| deformed \| \| MK-MalinoSvetiNikole15_2017_H12 \| 5.4 \| 10.8 \| single head \| deformed \| \| MK-MalinoSvetiNikole15_2017_H13 \| 6.5 \| 10.6 \| single head \| deformed \| \| MK-MalinoSvetiNikole15_2017_H14 \| 9.9 \| 8.5 \| single head \| deformed \| \| MK-MalinoSvetiNikole15_2017_H15 \| 6.7 \| 11.0 \| single head \| deformed \| \| MK-MalinoSvetiNikole15_2017_H16 \| 5.0 \| 11.0 \| single head \| deformed \| \| MK-MalinoSvetiNikole15_2017_H17 \| 5.7 \| 10.2 \| single head \| deformed \| \| MK-MalinoSvetiNikole15_2017_H18 \| 5.0 \| 10.7 \| single head \| deformed \| \| MK-MalinoSvetiNikole15_2017_H19 \| 6.0 \| 9.9 \| single head \| deformed \| \| MK-MalinoSvetiNikole15_2017_H20 \| 6.1 \| 10.9 \| single head \| deformed \| \| MK-MalinoSvetiNikole15_2017_H41 \| 4.7 \| 8.5 \| single head \| normal \| \| MK-MalinoSvetiNikole15_2017_H42 \| 2.8 \| 10.2 \| single head \| normal \| \| MK-MalinoSvetiNikole15_2017_H43 \| 2.7 \| 9.8 \| single head \| normal \| \| MK-MalinoSvetiNikole15_2017_H44 \| 0.0 \| 0.0 \| single head \| normal \| \| MK-MalinoSvetiNikole15_2017_H45 \| 2.9 \| 6.4 \| single head \| normal \| \| MK-MalinoSvetiNikole15_2017_H46 \| 5.3 \| 10.2 \| single head \| normal \| \| MK-MalinoSvetiNikole15_2017_H47 \| 6.8 \| 9.0 \| single head \| normal \| \| RO-TarguMures31_2017_H21 \| 8.0 \| 6.3 \| single head \| deformed \| \| RO-TarguMures31_2017_H22 \| 7.9 \| 0.0 \| single head \| deformed \| \| RO-TarguMures31_2017_H23 \| 8.4 \| 0.0 \| single head \| deformed \| \| RO-TarguMures31_2017_H24 \| 8.5 \| 0.0 \| single head \| deformed \| \| RO-TarguMures31_2017_H25 \| 8.6 \| 0.0 \| single head \| deformed \| \| RO-TarguMures31_2017_H26 \| 8.4 \| 6.4 \| single head \| deformed \| \| RO-TarguMures31_2017_H27 \| 8.2 \| 0.0 \| single head \| deformed \| \| RO-TarguMures31_2017_H28 \| 8.1 \| 7.0 \| single head \| deformed \| \| RO-TarguMures31_2017_H29 \| 8.5 \| 6.4 \| single head \| deformed \| \| RO-TarguMures31_2017_H30 \| 8.3 \| 0.0 \| single head \| deformed \| \| RO-TarguMures31_2017_H51 \| 4.8 \| 0.0 \| single head \| normal \| \| RO-TarguMures31_2017_H52 \| 4.2 \| 0.0 \| single head \| normal \| \| RO-TarguMures31_2017_H53 \| 7.6 \| 0.0 \| single head \| normal \| \| RO-TarguMures31_2017_H54 \| 3.3 \| 0.0 \| single head \| normal \| \| RO-TarguMures31_2017_H55 \| 2.7 \| 0.0 \| single head \| normal \| \| RO-TarguMures31_2017_H56 \| 2.8 \| 0.0 \| single head \| normal \| \| RO-TarguMures31_2017_H57 \| 3.5 \| 0.0 \| single head \| normal \| \| RO-TarguMures31_2017_H58 \| 0.0 \| 0.0 \| single head \| normal \| \| RO-TarguMures31_2017_H59 \| 6.1 \| 0.0 \| single head \| normal \| \| RO-TarguMures31_2017_H60 \| 6.2 \| 6.7 \| single head \| normal \| \| RS-Belgrade29_2017_H91 \| 2.2 \| 3.3 \| single head \| normal \| \| RS-Belgrade29_2017_H92 \| 2.9 \| 3.8 \| single head \| normal \| \| RS-Belgrade29_2017_H93 \| 3.5 \| 4.3 \| single head \| normal \| \| RS-Belgrade29_2017_H94 \| 2.9 \| 3.5 \| single head \| normal \| \| RS-Belgrade29_2017_H95 \| 4.3 \| 4.2 \| single head \| normal \| \| RS-Belgrade29_2017_H96 \| 3.4 \| 4.1 \| single head \| normal \| \| RS-Belgrade29_2017_H97 \| 3.2 \| 3.9 \| single head \| normal \| \| UK-SherburnInElmet35_2017_H186 \| 2.6 \| 9.5 \| single head \| deformed \| \| UK-SherburnInElmet35_2017_H187 \| 3.5 \| 9.6 \| single head \| deformed \| \| UK-SherburnInElmet35_2017_H188 \| 0.0 \| 9.5 \| single head \| deformed \| \| UK-SherburnInElmet35_2017_H189 \| 3.7 \| 9.6 \| single head \| deformed \| \| UK-SherburnInElmet35_2017_H190 \| 2.1 \| 9.6 \| single head \| deformed \| \| UK-SherburnInElmet35_2017_H191 \| 3.4 \| 9.5 \| single head \| deformed \| \| UK-SherburnInElmet36_2017_H192 \| 2.9 \| 7.1 \| single head \| deformed \| \| UK-SherburnInElmet36_2017_H193 \| 2.1 \| 9.5 \| single head \| deformed \| \| UK-SherburnInElmet36_2017_H194 \| 3.2 \| 9.6 \| single head \| deformed \| \| DE-HohenNeuendorf_2015_V55 \| 6.4 \| 10.8 \| *V. destructor* mite \| NA \| \| DE-HohenNeuendorf_2015_V56 \| 5.6 \| 8.8 \| *V. destructor* mite \| NA \| \| DE-HohenNeuendorf_2015_V57 \| 4.3 \| 10.2 \| *V. destructor* mite \| NA \| \| DE-HohenNeuendorf_2015_V58 \| 6.2 \| 10.1 \| *V. destructor* mite \| NA \| \| DE-HohenNeuendorf_2015_V59 \| 6.0 \| 10.9 \| *V. destructor* mite \| NA \| \| ES-Arbeiza21_2017_V31 \| 4.0 \| 8.6 \| *V. destructor* mite \| NA \| \| ES-Arbeiza21_2017_V32 \| 5.6 \| 5.8 \| *V. destructor* mite \| NA \| \| ES-Arbeiza21_2017_V33 \| 6.4 \| 10.3 \| *V. destructor* mite \| NA \| \| ES-Arbeiza21_2017_V34 \| 3.7 \| 6.3 \| *V. destructor* mite \| NA \| \| ES-Arbeiza21_2017_V35 \| 5.5 \| 0.0 \| *V. destructor* mite \| NA \| \| FR-Caumont8_2017_V16 \| 4.2 \| 8.7 \| *V. destructor* mite \| NA \| \| FR-Caumont8_2017_V17 \| 3.2 \| 6.2 \| *V. destructor* mite \| NA \| \| FR-Caumont8_2017_V18 \| 0.0 \| 10.0 \| *V. destructor* mite \| NA \| \| FR-Caumont8_2017_V19 \| 5.0 \| 10.1 \| *V. destructor* mite \| NA \| \| FR-Caumont8_2017_V20 \| 6.1 \| 9.3 \| *V. destructor* mite \| NA \| \| FR-Caumont6_2017_V11 \| 7.2 \| 8.0 \| *V. destructor* mite \| NA \| \| FR-Caumont6_2017_V12 \| 3.8 \| 8.8 \| *V. destructor* mite \| NA \| \| FR-Caumont6_2017_V13 \| 7.0 \| 0.0 \| *V. destructor* mite \| NA \| \| FR-Caumont6_2017_V14 \| 7.1 \| 9.3 \| *V. destructor* mite \| NA \| \| FR-Caumont6_2017_V15 \| 5.2 \| 10.4 \| *V. destructor* mite \| NA \| \| FR-Sophia2_2017_V1 \| 6.8 \| 0.0 \| *V. destructor* mite \| NA \| \| FR-Sophia2_2017_V2 \| 3.4 \| 11.1 \| *V. destructor* mite \| NA \| \| FR-Sophia2_2017_V3 \| 7.4 \| 7.3 \| *V. destructor* mite \| NA \| \| FR-Sophia2_2017_V4 \| 6.9 \| 6.8 \| *V. destructor* mite \| NA \| \| FR-Sophia2_2017_V5 \| 6.1 \| 6.1 \| *V. destructor* mite \| NA \| \| HR-HrvatskaKostajnica50_2017_V51 \| 3.3 \| 0.0 \| *V. destructor* mite \| NA \| \| HR-HrvatskaKostajnica50_2017_V52 \| 6.1 \| 0.0 \| *V. destructor* mite \| NA \| \| HR-HrvatskaKostajnica50_2017_V53 \| 8.0 \| 6.7 \| *V. destructor* mite \| NA \| \| HR-HrvatskaKostajnica50_2017_V54 \| 3.6 \| 0.0 \| *V. destructor* mite \| NA \| \| HR-HrvatskaKostajnica50_2017_V55 \| 6.0 \| 6.2 \| *V. destructor* mite \| NA \| \| IT-Palermo4_2017_V6 \| 7.6 \| 0.0 \| *V. destructor* mite \| NA \| \| IT-Palermo4_2017_V7 \| 6.4 \| 0.0 \| *V. destructor* mite \| NA \| \| IT-Palermo4_2017_V8 \| 9.5 \| 0.0 \| *V. destructor* mite \| NA \| \| IT-Palermo4_2017_V9 \| 7.2 \| 7.5 \| *V. destructor* mite \| NA \| \| IT-Palermo4_2017_V10 \| 7.1 \| 8.0 \| *V. destructor* mite \| NA \| \| MK-MalinoSvetiNikole15_2017_V16 \| 7.8 \| 10.2 \| *V. destructor* mite \| NA \| \| MK-MalinoSvetiNikole15_2017_V17 \| 7.2 \| 11.0 \| *V. destructor* mite \| NA \| \| MK-MalinoSvetiNikole15_2017_V18 \| 6.8 \| 9.0 \| *V. destructor* mite \| NA \| \| MK-MalinoSvetiNikole15_2017_V19 \| 4.4 \| 10.0 \| *V. destructor* mite \| NA \| \| MK-MalinoSvetiNikole15_2017_V20 \| 3.3 \| 8.8 \| *V. destructor* mite \| NA \| \| RO-TarguMures31_2017_V41 \| 9.1 \| 0.0 \| *V. destructor* mite \| NA \| \| RO-TarguMures31_2017_V42 \| 7.0 \| 0.0 \| *V. destructor* mite \| NA \| \| RO-TarguMures31_2017_V43 \| 7.4 \| 7.7 \| *V. destructor* mite \| NA \| \| RO-TarguMures31_2017_V44 \| 7.0 \| 0.0 \| *V. destructor* mite \| NA \| \| RO-TarguMures31_2017_V45 \| 8.0 \| 7.1 \| *V. destructor* mite \| NA \| \| RS-Belgrade29_2017_V36 \| 3.6 \| 0.0 \| *V. destructor* mite \| NA \| \| RS-Belgrade29_2017_V37 \| 6.8 \| 0.0 \| *V. destructor* mite \| NA \| \| RS-Belgrade29_2017_V38 \| 0.0 \| 0.0 \| *V. destructor* mite \| NA \| \| RS-Belgrade29_2017_V39 \| 0.0 \| 0.0 \| *V. destructor* mite \| NA \| \| RS-Belgrade29_2017_V40 \| 0.0 \| 0.0 \| *V. destructor* mite \| NA \| \| UK-SherburnInElmet35_2017_V46 \| 0.0 \| 9.7 \| *V. destructor* mite \| NA \| \| UK-SherburnInElmet35_2017_V47 \| 3.9 \| 8.9 \| *V. destructor* mite \| NA \| \| UK-SherburnInElmet35_2017_V48 \| 4.1 \| 6.1 \| *V. destructor* mite \| NA \| \| UK-SherburnInElmet35_2017_V49 \| 3.2 \| 9.7 \| *V. destructor* mite \| NA \| \| UK-SherburnInElmet35_2017_V50 \| 0.0 \| 9.0 \| *V. destructor* mite \| NA \| | |  |
| --- | --- | --- | --- | --- | --- | --- | --- | --- | --- | --- | --- | --- | --- | --- | --- | --- | --- | --- | --- | --- | --- | --- | --- | --- | --- | --- | --- | --- | --- | --- | --- | --- | --- | --- | --- | --- | --- | --- | --- | --- | --- | --- | --- | --- | --- | --- | --- | --- | --- | --- | --- | --- | --- | --- | --- | --- | --- | --- | --- | --- | --- | --- | --- | --- | --- | --- | --- | --- | --- | --- | --- | --- | --- | --- | --- | --- | --- | --- | --- | --- | --- | --- | --- | --- | --- | --- | --- | --- | --- | --- | --- | --- | --- | --- | --- | --- | --- | --- | --- | --- | --- | --- | --- | --- | --- | --- | --- | --- | --- | --- | --- | --- | --- | --- | --- | --- | --- | --- | --- | --- | --- | --- | --- | --- | --- | --- | --- | --- | --- | --- | --- | --- | --- | --- | --- | --- | --- | --- | --- | --- | --- | --- | --- | --- | --- | --- | --- | --- | --- | --- | --- | --- | --- | --- | --- | --- | --- | --- | --- | --- | --- | --- | --- | --- | --- | --- | --- | --- | --- | --- | --- | --- | --- | --- | --- | --- | --- | --- | --- | --- | --- | --- | --- | --- | --- | --- | --- | --- | --- | --- | --- | --- | --- | --- | --- | --- | --- | --- | --- | --- | --- | --- | --- | --- | --- | --- | --- | --- | --- | --- | --- | --- | --- | --- | --- | --- | --- | --- | --- | --- | --- | --- | --- | --- | --- | --- | --- | --- | --- | --- | --- | --- | --- | --- | --- | --- | --- | --- | --- | --- | --- | --- | --- | --- | --- | --- | --- | --- | --- | --- | --- | --- | --- | --- | --- | --- | --- | --- | --- | --- | --- | --- | --- | --- | --- | --- | --- | --- | --- | --- | --- | --- | --- | --- | --- | --- | --- | --- | --- | --- | --- | --- | --- | --- | --- | --- | --- | --- | --- | --- | --- | --- | --- | --- | --- | --- | --- | --- | --- | --- | --- | --- | --- | --- | --- | --- | --- | --- | --- | --- | --- | --- | --- | --- | --- | --- | --- | --- | --- | --- | --- | --- | --- | --- | --- | --- | --- | --- | --- | --- | --- | --- | --- | --- | --- | --- | --- | --- | --- | --- | --- | --- | --- | --- | --- | --- | --- | --- | --- | --- | --- | --- | --- | --- | --- | --- | --- | --- | --- | --- | --- | --- | --- | --- | --- | --- | --- | --- | --- | --- | --- | --- | --- | --- | --- | --- | --- | --- | --- | --- | --- | --- | --- | --- | --- | --- | --- | --- | --- | --- | --- | --- | --- | --- | --- | --- | --- | --- | --- | --- | --- | --- | --- | --- | --- | --- | --- | --- | --- | --- | --- | --- | --- | --- | --- | --- | --- | --- | --- | --- | --- | --- | --- | --- | --- | --- | --- | --- | --- | --- | --- | --- | --- | --- | --- | --- | --- | --- | --- | --- | --- | --- | --- | --- | --- | --- | --- | --- | --- | --- | --- | --- | --- | --- | --- | --- | --- | --- | --- | --- | --- | --- | --- | --- | --- | --- | --- | --- | --- | --- | --- | --- | --- | --- | --- | --- | --- | --- | --- | --- | --- | --- | --- | --- | --- | --- | --- | --- | --- | --- | --- | --- | --- | --- | --- | --- | --- | --- | --- | --- | --- | --- | --- | --- | --- | --- | --- | --- | --- | --- | --- | --- | --- | --- | --- | --- | --- | --- | --- | --- | --- | --- | --- | --- | --- | --- | --- | --- | --- | --- | --- | --- | --- | --- | --- | --- | --- | --- | --- | --- | --- | --- | --- | --- | --- | --- | --- | --- | --- | --- | --- | --- | --- | --- | --- | --- | --- | --- | --- | --- | --- | --- | --- | --- | --- | --- | --- | --- | --- | --- | --- | --- | --- | --- | --- | --- | --- | --- | --- | --- | --- | --- | --- | --- | --- | --- | --- | --- | --- | --- | --- | --- | --- | --- | --- | --- | --- | --- | --- | --- | --- | --- | --- | --- | --- | --- | --- | --- | --- | --- | --- | --- | --- | --- | --- | --- | --- | --- | --- | --- | --- | --- | --- | --- | --- | --- | --- | --- | --- | --- | --- | --- | --- | --- | --- | --- | --- | --- | --- | --- | --- | --- | --- | --- | --- | --- | --- | --- | --- | --- | --- | --- | --- | --- | --- | --- | --- | --- | --- | --- | --- | --- | --- | --- | --- | --- | --- | --- | --- | --- | --- | --- | --- | --- | --- | --- | --- | --- | --- | --- | --- | --- | --- | --- | --- | --- | --- | --- | --- | --- | --- | --- | --- | --- | --- | --- | --- | --- | --- | --- | --- | --- | --- | --- | --- | --- | --- | --- | --- | --- | --- | --- | --- | --- | --- | --- | --- | --- | --- | --- | --- | --- | --- | --- | --- | --- | --- | --- | --- | --- | --- | --- | --- | --- | --- | --- | --- | --- | --- | --- | --- | --- | --- | --- | --- | --- | --- | --- | --- | --- | --- | --- | --- | --- | --- | --- | --- | --- | --- | --- | --- | --- | --- | --- | --- | --- | --- | --- | --- | --- | --- | --- | --- | --- | --- | --- | --- | --- | --- | --- | --- | --- | --- | --- | --- | --- | --- | --- | --- | --- | --- | --- | --- | --- | --- | --- | --- | --- | --- | --- | --- | --- | --- | --- | --- | --- | --- | --- | --- | --- | --- | --- | --- | --- | --- | --- | --- | --- | --- | --- | --- | --- | --- | --- | --- | --- | --- | --- | --- | --- | --- | --- | --- | --- | --- | --- | --- | --- | --- | --- | --- | --- | --- | --- | --- | --- | --- | --- | --- | --- | --- | --- | --- | --- | --- | --- | --- | --- | --- | --- | --- | --- | --- | --- | --- | --- | --- | --- | --- | --- | --- | --- | --- | --- | --- | --- | --- | --- | --- | --- | --- | --- | --- | --- | --- | --- | --- | --- | --- | --- | --- | --- | --- | --- | --- | --- | --- | --- | --- | --- | --- | --- | --- | --- | --- | --- | --- | --- | --- | --- | --- | --- | --- | --- | --- | --- | --- | --- | --- | --- | --- | --- | --- | --- | --- | --- | --- | --- | --- | --- | --- | --- | --- | --- | --- | --- | --- | --- | --- | --- | --- | --- | --- | --- | --- | --- | --- | --- | --- | --- | --- | --- | --- | --- | --- | --- | --- | --- | --- | --- | --- | --- | --- | --- | --- | --- | --- | --- | --- | --- | --- | --- | --- | --- | --- | --- | --- | --- | --- | --- | --- | --- | --- | --- | --- | --- | --- | --- | --- | --- | --- | --- | --- | --- | --- | --- | --- | --- | --- | --- | --- | --- | --- | --- | --- | --- | --- | --- | --- | --- | --- | --- | --- | --- | --- | --- | --- | --- | --- | --- | --- | --- | --- | --- | --- | --- | --- | --- | --- | --- | --- | --- | --- | --- | --- | --- | --- | --- | --- | --- | --- | --- | --- | --- | --- | --- | --- | --- | --- | --- | --- | --- | --- | --- | --- | --- | --- | --- | --- | --- | --- | --- | --- | --- | --- | --- | --- | --- | --- | --- | --- | --- | --- | --- | --- | --- | --- | --- | --- | --- | --- | --- | --- | --- | --- | --- | --- | --- | --- | --- | --- | --- | --- | --- | --- | --- | --- | --- | --- | --- | --- | --- | --- | --- | --- | --- | --- | --- | --- | --- | --- | --- | --- | --- | --- | --- | --- | --- | --- | --- | --- | --- | --- | --- | --- | --- | --- | --- | --- | --- | --- | --- | --- | --- | --- | --- | --- | --- | --- | --- | --- | --- | --- | --- | --- | --- | --- | --- | --- | --- | --- | --- | --- | --- | --- | --- | --- | --- | --- | --- | --- | --- | --- | --- | --- | --- | --- | --- | --- | --- | --- | --- | --- | --- | --- | --- | --- | --- | --- | --- | --- | --- | --- | --- | --- | --- | --- | --- | --- | --- | --- | --- | --- | --- | --- | --- | --- | --- | --- | --- | --- | --- | --- | --- | --- | --- | --- | --- | --- | --- | --- | --- | --- | --- | --- | --- | --- | --- | --- | --- | --- | --- | --- | --- | --- | --- | --- | --- | --- | --- | --- | --- | --- | --- | --- | --- | --- | --- | --- | --- | --- | --- | --- | --- | --- | --- | --- | --- | --- | --- | --- | --- | --- | --- | --- | --- | --- | --- | --- | --- | --- | --- | --- | --- | --- | --- | --- | --- | --- | --- | --- | --- | --- | --- | --- | --- | --- | --- | --- | --- | --- | --- | --- | --- | --- | --- | --- | --- | --- | --- | --- | --- | --- | --- | --- | --- | --- | --- | --- | --- | --- | --- | --- | --- | --- | --- | --- | --- | --- | --- | --- | --- | --- | --- | --- | --- | --- | --- | --- | --- | --- | --- | --- | --- | --- | --- | --- | --- | --- | --- | --- | --- | --- | --- | --- | --- | --- | --- | --- | --- | --- | --- | --- | --- | --- | --- | --- | --- | --- | --- | --- | --- | --- | --- | --- | --- | --- | --- | --- | --- | --- | --- | --- | --- | --- | --- | --- | --- | --- | --- | --- | --- | --- | --- | --- | --- | --- | --- | --- | --- | --- | --- | --- | --- | --- | --- | --- | --- | --- | --- | --- | --- | --- | --- | --- | --- | --- | --- | --- | --- | --- | --- | --- | --- | --- | --- | --- | --- | --- | --- | --- | --- | --- | --- | --- | --- | --- | --- | --- | --- | --- | --- | --- | --- | --- | --- | --- | --- | --- | --- | --- | --- | --- | --- | --- | --- | --- | --- | --- | --- | --- | --- | --- | --- | --- | --- | --- | --- | --- | --- | --- | --- | --- | --- | --- | --- | --- | --- | --- | --- | --- | --- | --- | --- | --- | --- | --- | --- | --- | --- | --- | --- | --- | --- | --- | --- | --- | --- | --- | --- | --- | --- | --- | --- | --- | --- | --- | --- | --- | --- | --- | --- | --- | --- | --- | --- | --- | --- | --- | --- | --- | --- | --- | --- | --- | --- | --- | --- | --- | --- | --- | --- | --- | --- | --- | --- | --- | --- | --- | --- | --- | --- | --- | --- | --- | --- | --- | --- | --- | --- | --- | --- | --- | --- | --- | --- | --- | --- | --- | --- | --- | --- | --- | --- | --- | --- | --- | --- | --- | --- | --- | --- | --- | --- | --- | --- | --- | --- | --- | --- | --- | --- | --- | --- | --- | --- | --- | --- | --- | --- | --- | --- | --- | --- | --- | --- | --- | --- | --- | --- | --- | --- | --- | --- | --- | --- | --- | --- | --- | --- | --- | --- | --- | --- | --- | --- | --- | --- | --- | --- | --- | --- | --- | --- | --- | --- | --- | --- | --- | --- | --- | --- | --- | --- | --- | --- | --- | --- | --- | --- | --- | --- | --- | --- | --- | --- | --- | --- | --- | --- | --- | --- | --- | --- | --- | --- | --- | --- | --- | --- | --- | --- | --- | --- | --- | --- | --- | --- | --- | --- | --- | --- | --- | --- | --- | --- | --- | --- | --- | --- | --- | --- | --- | --- | --- | --- | --- | --- | --- | --- | --- | --- | --- | --- | --- | --- | --- | --- | --- | --- | --- | --- | --- | --- | --- | --- | --- | --- | --- | --- | --- | --- | --- | --- | --- | --- | --- | --- | --- | --- | --- | --- | --- | --- | --- | --- | --- | --- | --- | --- | --- | --- | --- | --- | --- | --- | --- | --- | --- | --- | --- | --- | --- | --- | --- | --- | --- | --- | --- | --- | --- | --- | --- | --- | --- | --- | --- | --- | --- | --- | --- | --- | --- | --- | --- | --- | --- | --- | --- | --- | --- | --- | --- | --- | --- | --- | --- | --- | --- | --- | --- | --- | --- | --- | --- | --- | --- | --- | --- | --- | --- | --- |
| **1.** Blue boxes indicate samples from varroa-free regions. | | |
| **2.** Red boxes indicate DWV-A loads above or equal to 6.0 log_10_ copies per individual bee, bee head, or mite. | | |
| **3.** Red boxes indicate DWV-B loads above or equal to 7.3 log_10_ copies per individual bee, bee head, or mite. | | |
| **4.** NA: not applicable. |  |  |


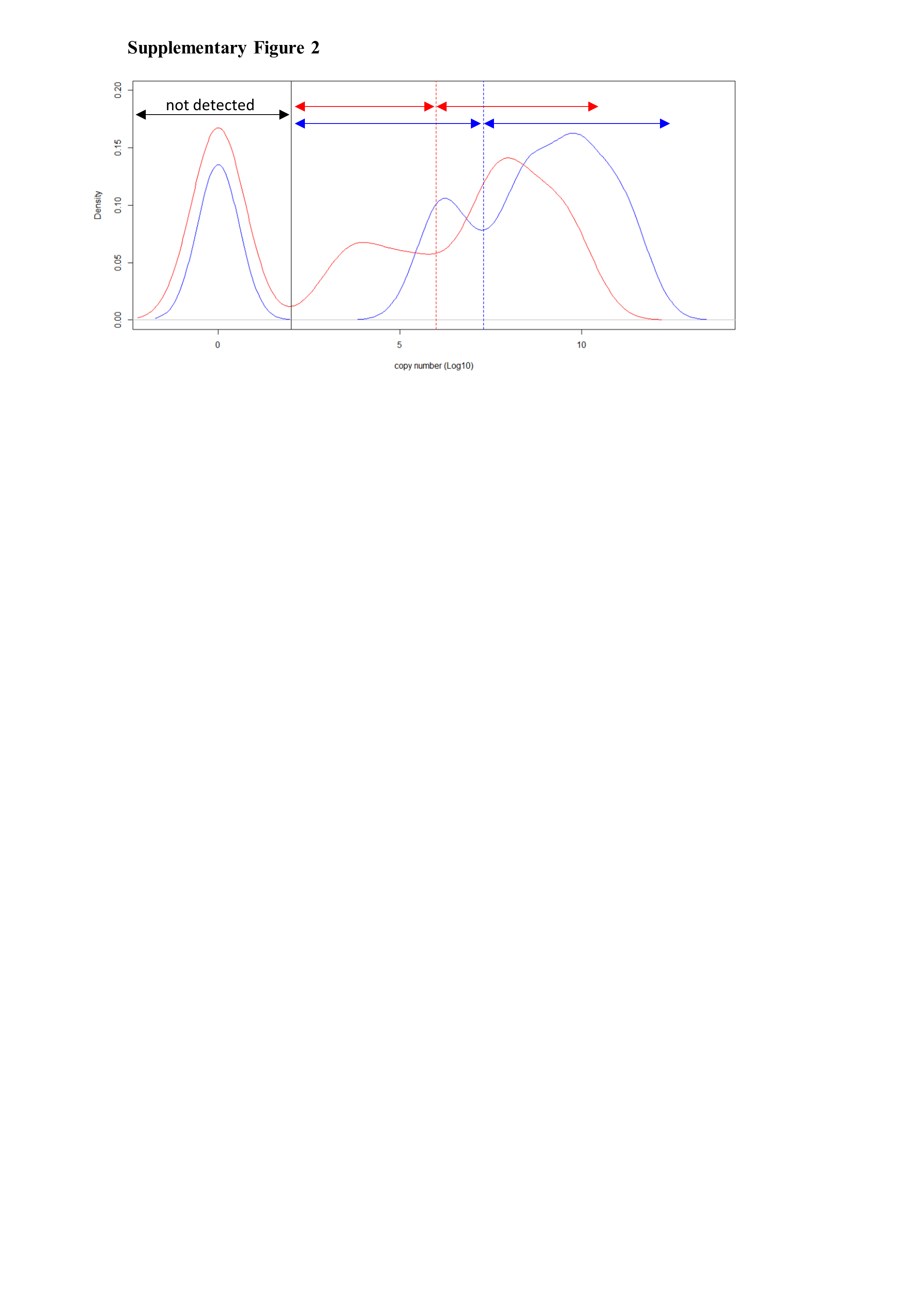


**Supplementary Figure S2.** Density estimations of DWV-A (red) and DWV-B (blue) as number of copies (log_10_ copies/bee) in honey bee pools. The black line corresponds to the threshold between samples in which DWV was detected (set at 2.0 log_10_ copies/bee). Thresholds were positioned between samples with low viral loads and those with high viral loads for DWV-A (set at 6.0 log_10_ copies/bee, red dashed line) and DWV-B (set at 7.3 log_10_ copies/bee, blue dashed line).

**
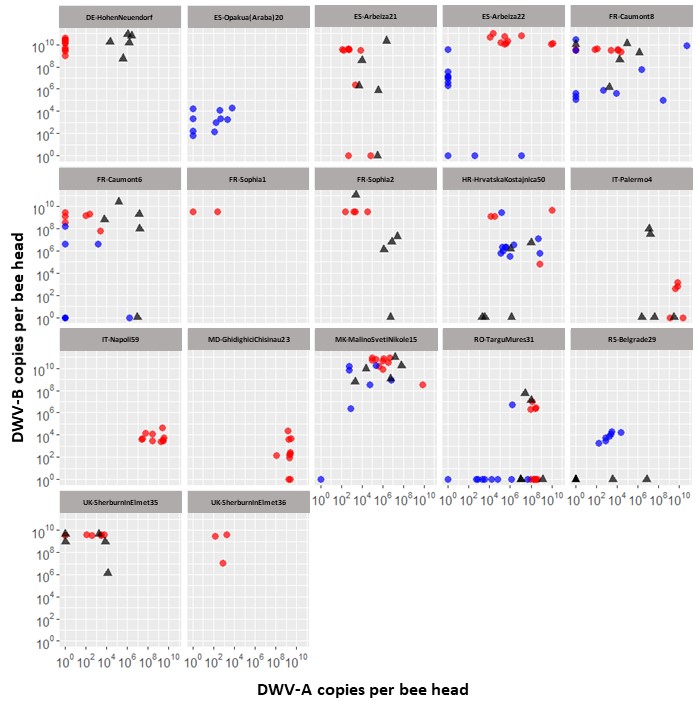
**

**Supplementary Figure S3.** DWV-A and DWV-B loads quantified in single honey bee heads and *V. destructor*. Distribution of viral loads measured in single heads from honey bees with deformed (red dots) and normal (blue dots) wings and *V. destructor* (triangles) according to the apiary origin. Country code: DE = Germany, ES = Spain, FR = France, HR = Croatia, IT = Italy, MD = Moldova, MK = North Macedonia, RO = Romania, RS = Republic of Serbia, UK = United Kingdom.

|  |
| --- |


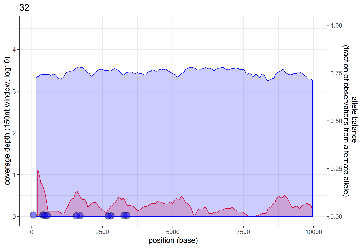

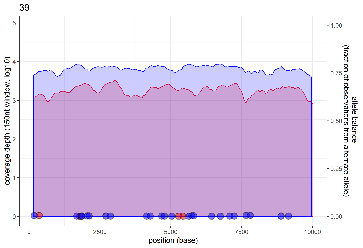


Croatia


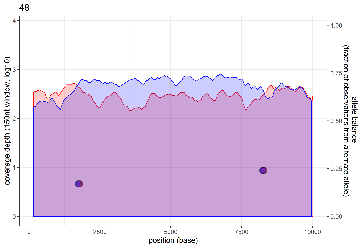

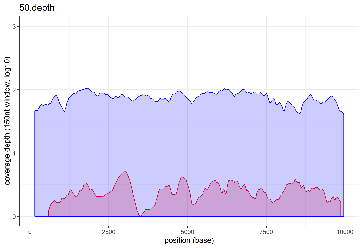

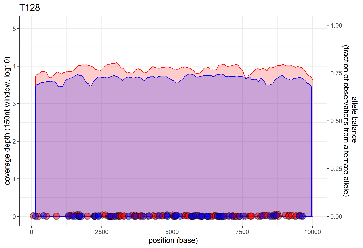

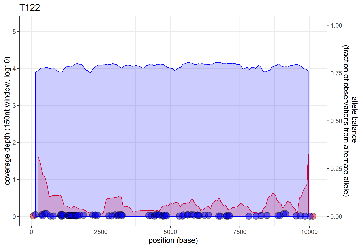

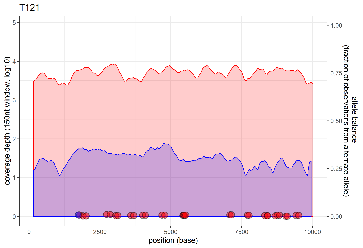

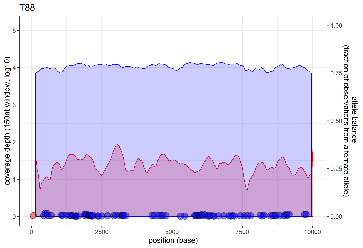

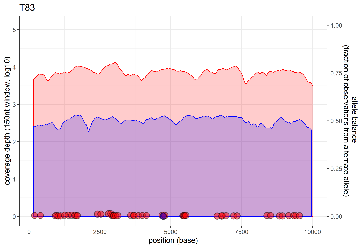


HR-HrvatskaKostajnica32_2017_P

HR-Sisak39_2017_P

HR-Sisak48_2017_P

HR-HrvatskaKostajnica50_2017_P

HR-HrvatskaKostajnica50_2017_H83

HR-HrvatskaKostajnica50_2017_H88

HR-HrvatskaKostajnica50_2017_H121

HR-HrvatskaKostajnica50_2017_H122

HR-HrvatskaKostajnica50_2017_H128

not-deformed

not-deformed

deformed

deformed

deformed


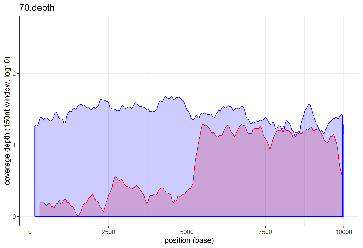


Finland

*V. destructor*-free

FI-Åland70_2016_P


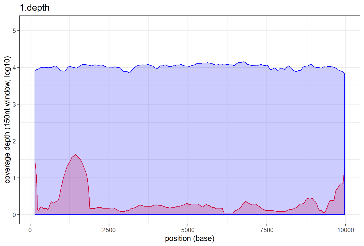

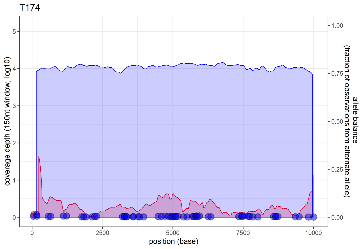

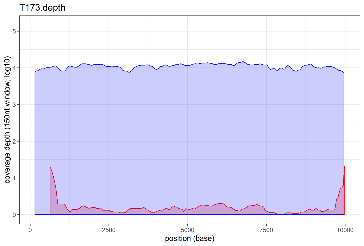

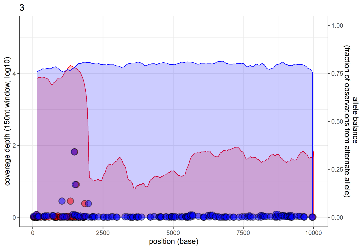


FR_Sophia1_2017_P

FR-Sophia2_2017_H173

FR-Sophia2_2017_H174

FR_Sophia3_2017_P


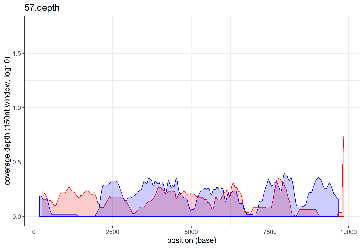

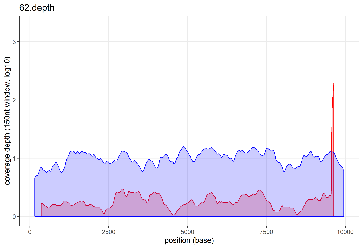

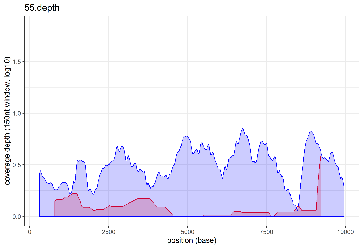

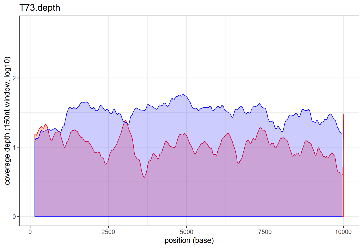

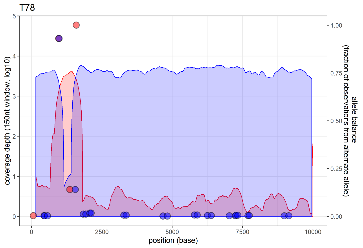

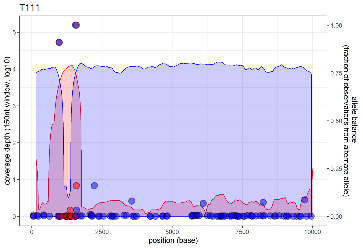

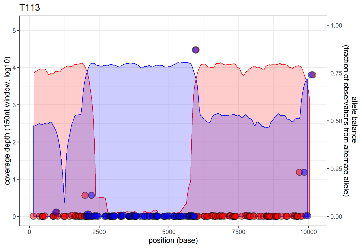

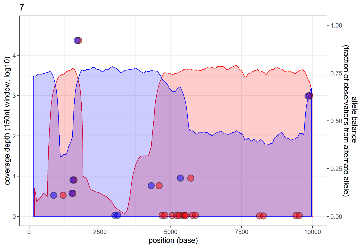


FR-Caumont6_2017_H73

FR-Caumont6_2017_H78

FR-Caumont6_2017_H111

FR-Caumont6_2017_H113


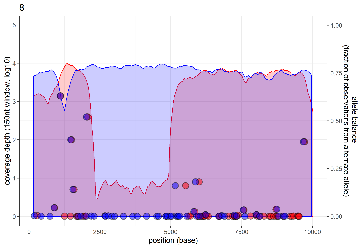

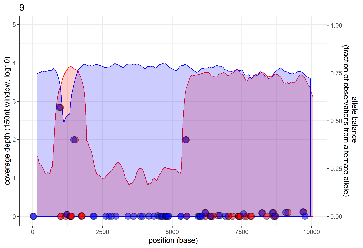

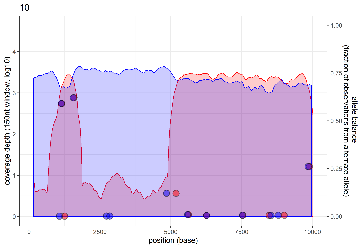


FR-Caumont7_2017_P

FR-Caumont8_2017_P

FR-Caumont9_2017_P

FR-Caumont10_2017_P

FR-Ouessant55_2011_P

FR-Ouessant57_2011_P

FR-Ouessant62_2011_P

*V. destructor*-free

Not-deformed

Not-deformed

deformed

deformed

*V. destructor*-free

*V. destructor*-free

deformed

deformed

France


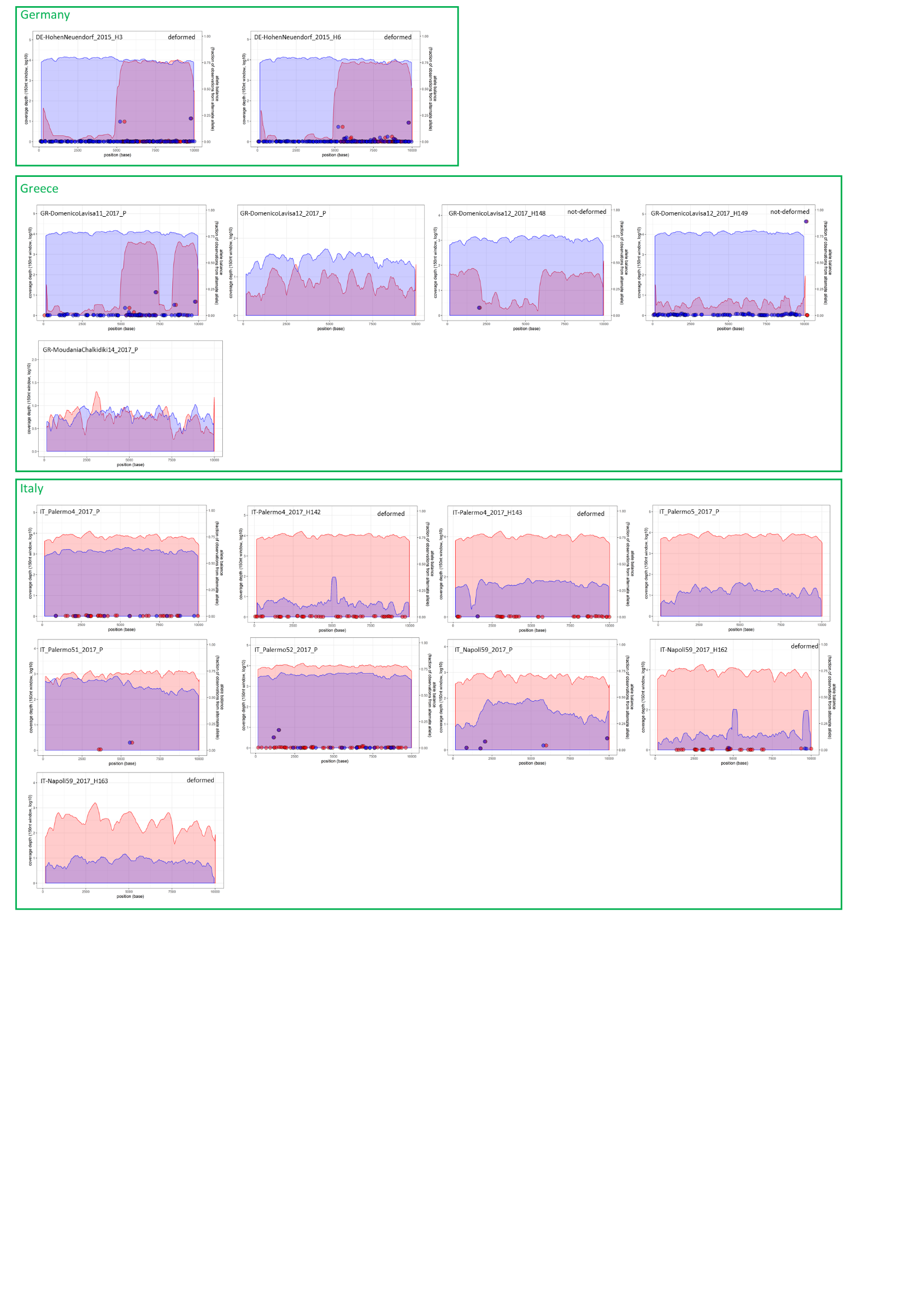

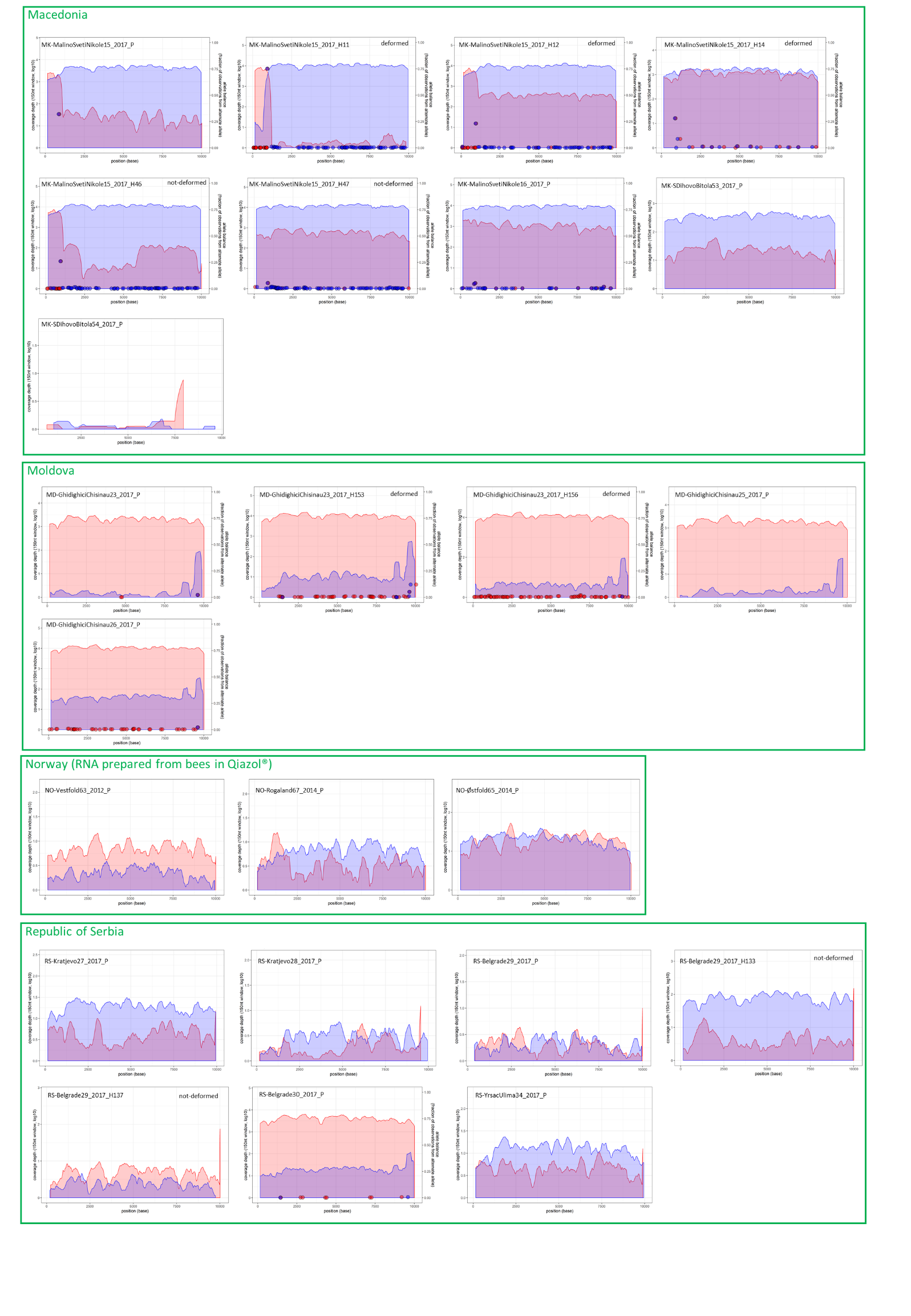

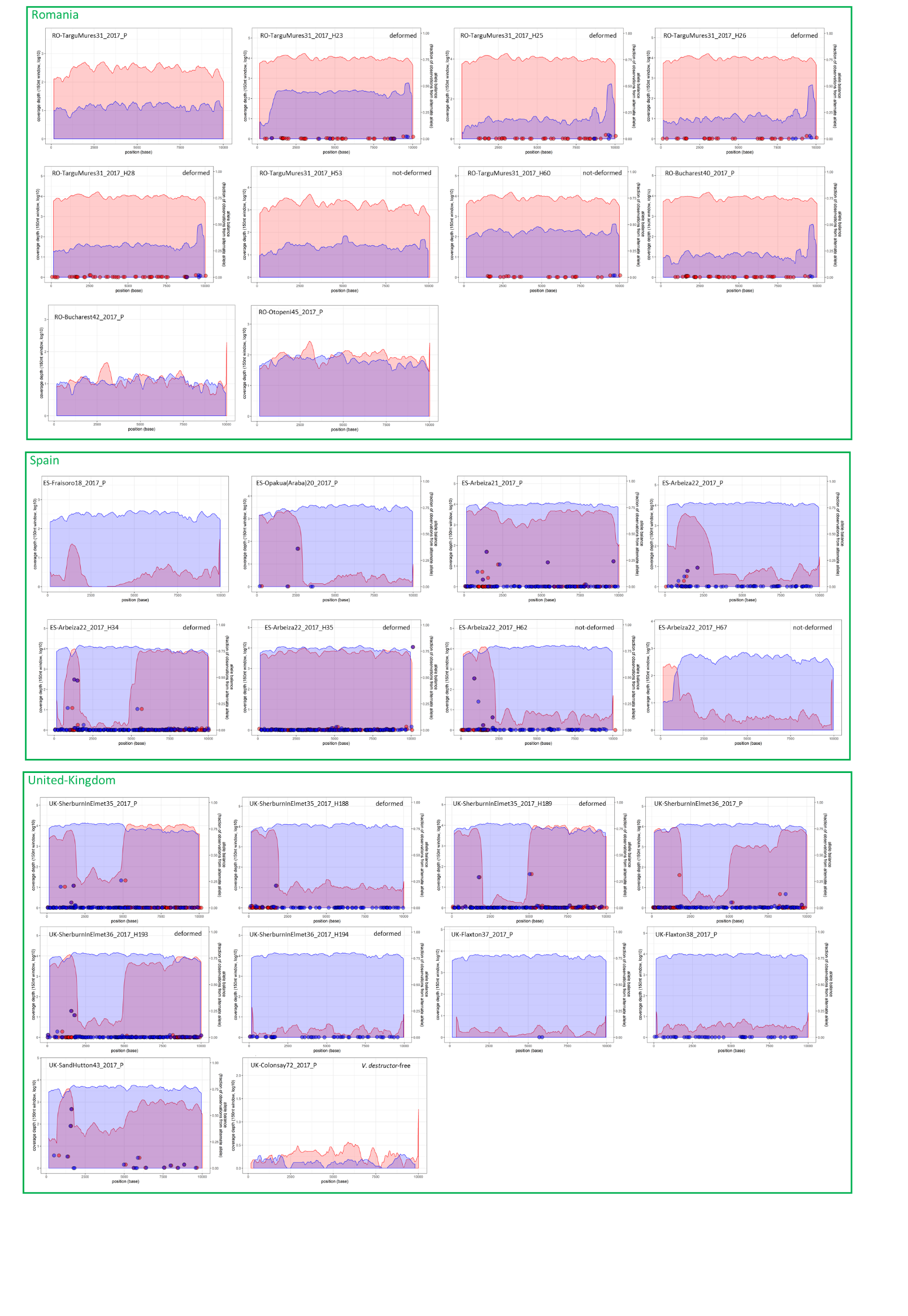


**Supplementary Figure S4.** Genome profiles of 93 sequenced samples. DWV-A and DWV-B coverage depth in log_10_ scale calculated in 150 nt windows along the genome are represented by red and blue graphs respectively. Dots represent the allele balance of the break end identified by LUMPY and SVtyper for DWV-A (red) and DWV-B (blue) when available. Samples are clustered according to the country of origin in green squares. Each sample name is written as follows: COUNTRYCODE_ApiaryColonyID_Year_Type. The type could be either P for pool of honey bees or Hxxx for single honey bee head. For example, HR-HrvatskaKostajnica32_2017_P corresponds to a pool of honey bees sampled in 2017 from colony 32 in the HrvatskaKostajnica apiary in Crotia (country code HR). For single bee heads, wing morphology is specified in the top right corner of the graph. Samples from *V. destructor*-free areas are specified in the top right corner of the graph.

| **Supplementary Table S2.** Characterization of read-based potential DWV-recombinant junctions using LUMPY and ViReMa tests.   \| **Sample** \| **DWV-A  genomic position  (LUMPY)** \| **DWV-B  genomic position (LUMPY)** \| **Number of pieces of evidence supporting the variant (PE+SR)** \| **Number of paired-end reads supporting the variant (PE)** \| **Number of split reads supporting the variant (SR)** \| **Read depth (DP)** \| **Sum of quality of alternate observations (QA)** \| **Allele balance, fraction of observations from alternate allele (QA/DP)** \| **DWV-A genomic position (ViReMa)** \| **DWV-B genomic position (ViReMa)** \| **Count ViReMa** \| **Offset between LUMPY and ViReMa positions on DWV-A** \| **Offset between LUMPY and ViReMa positions on DWV-B** \| **LUMPY/ViReMa junctions guided reconstruction of recombinant genome** \| **Recombinant junction within a single contig ^1^** \| **Corresponding genome name** \| \| --- \| --- \| --- \| --- \| --- \| --- \| --- \| --- \| --- \| --- \| --- \| --- \| --- \| --- \| --- \| --- \| --- \| \| DE-HohenNeuendorf_2015_H3 \| 5509 \| 5229 \| 221 \| 221 \| 0 \| 12020 \| 2336 \| 0.19 \| 5316 \| 5293 \| 427 \| 193 \| 64 \| Yes \| No \| G2 \| \| DE-HohenNeuendorf_2015_H3 \| 9801 \| 9772 \| 1568 \| 1568 \| 0 \| 7607 \| 1674 \| 0.22 \| 9776 \| 9755 \| 46 \| 25 \| 17 \| Yes \| Yes \| G2 \| \| DE-HohenNeuendorf_2015_H6 \| 5509 \| 5226 \| 197 \| 197 \| 0 \| 10159 \| 1465 \| 0.14 \| 5316 \| 5293 \| 629 \| 193 \| 67 \| Yes \| No \| G2 \| \| DE-HohenNeuendorf_2015_H6 \| 9799 \| 9766 \| 1194 \| 1194 \| 0 \| 6505 \| 1185 \| 0.18 \| 9776 \| 9755 \| 94 \| 23 \| 11 \| Yes \| Yes \| G2 \| \| ES-Arbeiza21_2017_P \| 1179 \| 872 \| 29 \| 29 \| 0 \| 9573 \| 1334 \| 0.14 \| No data ^2^ \| No data \| No data \| N/A ^3^ \| N/A \| No \| N/A \| N/A \| \| ES-Arbeiza21_2017_P \| 1474 \| 1445 \| 4582 \| 2545 \| 2037 \| 19067 \| 6252 \| 0.33 \| No data \| No data \| No data \| N/A \| N/A \| No \| N/A \| N/A \| \| ES-Arbeiza21_2017_P \| 1552 \| 1520 \| 119 \| 0 \| 119 \| 17983 \| 1491 \| 0.083 \| No data \| No data \| No data \| N/A \| N/A \| No \| N/A \| N/A \| \| ES-Arbeiza21_2017_P \| 2201 \| 2300 \| 370 \| 370 \| 0 \| 8718 \| 1799 \| 0.21 \| No data \| No data \| No data \| N/A \| N/A \| No \| N/A \| N/A \| \| ES-Arbeiza21_2017_P \| 5419 \| 5412 \| 47 \| 46 \| 1 \| 9165 \| 2069 \| 0.23 \| No data \| No data \| No data \| N/A \| N/A \| No \| N/A \| N/A \| \| ES-Arbeiza21_2017_P \| 9671 \| 9655 \| 1058 \| 1058 \| 0 \| 2999 \| 720 \| 0.24 \| No data \| No data \| No data \| N/A \| N/A \| No \| N/A \| N/A \| \| ES-Arbeiza22_2017_H34 \| 1176 \| 878 \| 254 \| 254 \| 0 \| 10821 \| 2226 \| 0.21 \| 962 \| 953 \| 344 \| 214 \| 75 \| No \| No \| N/A \| \| ES-Arbeiza22_2017_H34 \| 1298 \| 1298 \| 3836 \| 3836 \| 0 \| 13016 \| 6283 \| 0.48 \| Not found ^4^ \| Not found \| Not found \| N/A \| N/A \| No \| Yes \| N/A \| \| ES-Arbeiza22_2017_H34 \| 1527 \| 1495 \| 1663 \| 59 \| 1604 \| 15152 \| 7052 \| 0.47 \| 1509 \| 1488 \| 1613 \| 18 \| 7 \| Yes \| Yes \| G2 \| \| ES-Arbeiza22_2017_H34 \| 5678 \| 5394 \| 235 \| 235 \| 0 \| 13317 \| 2660 \| 0.2 \| 5412 \| 5389 \| 798 \| 266 \| 5 \| Yes \| No \| G2 \| \| ES-Arbeiza22_2017_H62 \| 887 \| 867 \| 2314 \| 2314 \| 0 \| 10723 \| 5232 \| 0.49 \| 868 \| 859 \| 16 \| 19 \| 8 \| Yes \| No \| G2 \| \| ES-Arbeiza22_2017_H62 \| 1397 \| 1145 \| 119 \| 119 \| 0 \| 13785 \| 3661 \| 0.27 \| 1145 \| 1122 \| 1 \| 252 \| 23 \| Yes \| No \| G3 \| \| ES-Arbeiza22_2017_H62 \| 2074 \| 2082 \| 54 \| 53 \| 1 \| 8722 \| 1019 \| 0.12 \| 2093 \| 2072 \| 154 \| 19 \| 10 \| No \| N/A \| N/A \| \| ES-Arbeiza22_2017_P \| 1227 \| 875 \| 40 \| 40 \| 0 \| 9247 \| 512 \| 0.055 \| 838 \| 829 \| 31 \| 389 \| 46 \| Yes \| No \| G2 \| \| ES-Arbeiza22_2017_P \| 1472 \| 1445 \| 750 \| 0 \| 750 \| 15960 \| 2323 \| 0.15 \| 1474 \| 1453 \| 2 \| 2 \| 8 \| No \| N/A \| N/A \| \| ES-Arbeiza22_2017_P \| 2111 \| 2099 \| 2034 \| 2034 \| 0 \| 9276 \| 1624 \| 0.18 \| 2093 \| 2072 \| 125 \| 18 \| 27 \| Yes \| No \| G2 \| \| ES-Opakua(Araba)20_2017_P \| 2645 \| 2598 \| 528 \| 528 \| 0 \| 1603 \| 574 \| 0.36 \| 2606 \| 2585 \| 180 \| 39 \| 13 \| Yes \| Yes \| G2 \| \| FR_Caumont10_2017_P \| 1152 \| 1143 \| 109 \| 108 \| 1 \| 2365 \| 1393 \| 0.59 \| 1135 \| 1112 \| 47 \| 17 \| 31 \| Yes \| Yes \| G1 \| \| FR_Caumont10_2017_P \| 1589 \| 1559 \| 2127 \| 1614 \| 513 \| 4595 \| 2833 \| 0.62 \| 1580 \| 1559 \| 264 \| 9 \| 0 \| Yes \| Yes \| G1 \| \| FR_Caumont10_2017_P \| 5187 \| 4854 \| 36 \| 36 \| 0 \| 3940 \| 455 \| 0.12 \| 5076 \| 5053 \| 14 \| 111 \| 199 \| Yes \| No \| G1 \| \| FR_Caumont10_2017_P \| 9886 \| 9845 \| 355 \| 355 \| 0 \| 1587 \| 418 \| 0.26 \| 9776 \| 9755 \| 204 \| 110 \| 90 \| Yes \| Yes \| G1 \| \| FR_Caumont7_2017_P \| 1205 \| 874 \| 79 \| 79 \| 0 \| 4125 \| 444 \| 0.11 \| 909 \| 900 \| 177 \| 296 \| 26 \| Yes \| Yes \| G1 \| \| FR_Caumont7_2017_P \| 1755 \| 1700 \| 2599 \| 2593 \| 6 \| 4344 \| 3998 \| 0.92 \| 1797 \| 1774 \| 12 \| 42 \| 74 \| Yes \| Yes \| G1 \| \| FR_Caumont7_2017_P \| 4590 \| 4308 \| 110 \| 110 \| 0 \| 2925 \| 481 \| 0.16 \| 4590 \| 4567 \| 324 \| 0 \| 259 \| Yes \| No \| G1 \| \| FR_Caumont7_2017_P \| 5705 \| 5342 \| 24 \| 24 \| 0 \| 5388 \| 1104 \| 0.2 \| 5478 \| 5455 \| 127 \| 227 \| 113 \| No \| N/A \| N/A \| \| FR_Caumont7_2017_P \| 9891 \| 9845 \| 1624 \| 1624 \| 0 \| 2351 \| 1472 \| 0.63 \| 9776 \| 9755 \| 133 \| 115 \| 90 \| Yes \| Yes \| G1 \| \| FR_Caumont8_2017_P \| 1111 \| 1105 \| 123 \| 122 \| 1 \| 5998 \| 3764 \| 0.63 \| 1135 \| 1112 \| 145 \| 24 \| 7 \| Yes \| Yes \| G1 \| \| FR_Caumont8_2017_P \| 1498 \| 1475 \| 4060 \| 3861 \| 199 \| 11224 \| 4489 \| 0.4 \| 1496 \| 1475 \| 274 \| 2 \| 0 \| Yes \| Yes \| G1 \| \| FR_Caumont8_2017_P \| 1589 \| 1553 \| 29 \| 0 \| 29 \| 12486 \| 1754 \| 0.14 \| Not found \| Not found \| Not found \| N/A \| N/A \| No \| N/A \| N/A \| \| FR_Caumont8_2017_P \| 2077 \| 2029 \| 3094 \| 3094 \| 0 \| 7905 \| 4107 \| 0.52 \| 2015 \| 1994 \| 235 \| 62 \| 35 \| No \| N/A \| N/A \| \| FR_Caumont8_2017_P \| 5528 \| 5157 \| 45 \| 45 \| 0 \| 7426 \| 1163 \| 0.16 \| 5514 \| 5491 \| 115 \| 14 \| 334 \| No \| N/A \| N/A \| \| FR_Caumont8_2017_P \| 6003 \| 5875 \| 23 \| 23 \| 0 \| 8445 \| 1505 \| 0.18 \| 5945 \| 5922 \| 119 \| 58 \| 47 \| No \| N/A \| N/A \| \| FR_Caumont8_2017_P \| 9720 \| 9680 \| 3251 \| 3251 \| 0 \| 7429 \| 2893 \| 0.39 \| 9695 \| 9674 \| 208 \| 25 \| 6 \| No \| N/A \| N/A \| \| FR_Caumont9_2017_P \| 990 \| 949 \| 2409 \| 2409 \| 0 \| 3466 \| 1989 \| 0.57 \| 972 \| 935 \| 498 \| 18 \| 14 \| Yes \| Yes \| G1 \| \| FR_Caumont9_2017_P \| 1526 \| 1475 \| 23 \| 13 \| 10 \| 4969 \| 1995 \| 0.4 \| Not found \| Not found \| Not found \| N/A \| N/A \| Yes \| Yes \| G1 \| \| FR_Caumont9_2017_P \| 5505 \| 5492 \| 2938 \| 2938 \| 0 \| 6943 \| 2752 \| 0.4 \| 5506 \| 5455 \| 595 \| 1 \| 37 \| No \| N/A \| N/A \| \| FR_Sophia3_2017_P \| 1340 \| 1039 \| 141 \| 141 \| 0 \| 15041 \| 1256 \| 0.084 \| 1135 \| 1112 \| 112 \| 205 \| 73 \| No \| N/A \| N/A \| \| FR_Sophia3_2017_P \| 1497 \| 1477 \| 5895 \| 4793 \| 1102 \| 30716 \| 10572 \| 0.34 \| Not found \| Not found \| Not found \| N/A \| N/A \| No \| N/A \| N/A \| \| FR_Sophia3_2017_P \| 1552 \| 1515 \| 13 \| 0 \| 13 \| 38620 \| 6372 \| 0.17 \| 1536 \| 1515 \| 11 \| 16 \| 0 \| No \| N/A \| N/A \| \| FR_Sophia3_2017_P \| 1848 \| 1982 \| 496 \| 493 \| 3 \| 11852 \| 837 \| 0.071 \| 1805 \| 1784 \| 486 \| 43 \| 198 \| Yes \| No \| G2 \| \| FR-Caumont6_2017_H111 \| 972 \| 979 \| 368 \| 365 \| 3 \| 8495 \| 7763 \| 0.91 \| 980 \| 971 \| 29 \| 8 \| 8 \| Yes \| Yes \| G1 \| \| FR-Caumont6_2017_H111 \| 1588 \| 2231 \| 14 \| 14 \| 0 \| 12441 \| 1963 \| 0.16 \| Not found \| Not found \| Not found \| N/A \| N/A \| No \| N/A \| N/A \| \| FR-Caumont6_2017_H111 \| 1589 \| 1559 \| 14430 \| 10506 \| 3924 \| 18465 \| 18447 \| 1 \| 1580 \| 1559 \| 2199 \| 9 \| 0 \| Yes \| Yes \| G1 \| \| FR-Caumont6_2017_H111 \| 1589 \| 3563 \| 19 \| 19 \| 0 \| 13944 \| 1110 \| 0.08 \| Not found \| Not found \| Not found \| N/A \| N/A \| No \| N/A \| N/A \| \| FR-Caumont6_2017_H111 \| 1589 \| 6113 \| 19 \| 19 \| 0 \| 16310 \| 1113 \| 0.068 \| Not found \| Not found \| Not found \| N/A \| N/A \| No \| N/A \| N/A \| \| FR-Caumont6_2017_H113 \| 1988 \| 2222 \| 131 \| 129 \| 2 \| 13745 \| 1447 \| 0.11 \| 2076 \| 2027 \| 38 \| 88 \| 195 \| Yes \| Yes \| G1 \| \| FR-Caumont6_2017_H113 \| 5973 \| 5949 \| 7991 \| 7991 \| 0 \| 9250 \| 8021 \| 0.87 \| 5973 \| 5922 \| 994 \| 0 \| 27 \| Yes \| Yes \| G1 \| \| FR-Caumont6_2017_H113 \| 9677 \| 9847 \| 31 \| 31 \| 0 \| 8087 \| 1884 \| 0.23 \| 9913 \| 9864 \| 3 \| 236 \| 17 \| Yes \| Yes \| G1 \| \| FR-Caumont6_2017_H78 \| 965 \| 977 \| 130 \| 127 \| 3 \| 3034 \| 2819 \| 0.93 \| 1093 \| 964 \| 7 \| 128 \| 13 \| Yes \| Yes \| G1 \| \| FR-Caumont6_2017_H78 \| 1589 \| 1559 \| 3814 \| 2522 \| 1292 \| 5999 \| 5988 \| 1 \| 1580 \| 1559 \| 535 \| 9 \| 0 \| Yes \| Yes \| G1 \| \| GR-DomenicoLavisa11_2017_P \| 5541 \| 5256 \| 35 \| 35 \| 0 \| 6531 \| 463 \| 0.071 \| 5301 \| 5278 \| 248 \| 240 \| 22 \| Yes \| Yes \| G2 \| \| GR-DomenicoLavisa11_2017_P \| 7282 \| 7227 \| 1902 \| 1902 \| 0 \| 10020 \| 2167 \| 0.22 \| 7250 \| 7229 \| 285 \| 32 \| 2 \| Yes \| Yes \| G2 \| \| GR-DomenicoLavisa11_2017_P \| 8547 \| 8435 \| 59 \| 58 \| 1 \| 8578 \| 884 \| 0.1 \| 8538 \| 8515 \| 963 \| 9 \| 80 \| Yes \| Yes \| G2 \| \| GR-DomenicoLavisa11_2017_P \| 9817 \| 9765 \| 962 \| 962 \| 0 \| 7295 \| 974 \| 0.13 \| 9776 \| 9755 \| 230 \| 41 \| 10 \| Yes \| Yes \| G2 \| \| IT-Palermo51_2017_P \| 5709 \| 5567 \| 11 \| 11 \| 0 \| 1012 \| 73 \| 0.072 \| 5577 \| 5554 \| 4 \| 132 \| 13 \| Yes \| No \| G3 \| \| IT-Palermo52_2017_P \| 1178 \| 1145 \| 875 \| 875 \| 0 \| 7986 \| 826 \| 0.1 \| 1136 \| 1112 \| 26 \| 42 \| 33 \| No \| N/A \| N/A \| \| IT-Palermo52_2017_P \| 1498 \| 1477 \| 198 \| 7 \| 191 \| 8788 \| 1491 \| 0.17 \| Not found \| Not found \| Not found \| N/A \| N/A \| No \| N/A \| N/A \| \| MD-GhidighiciChisinau23_2017_H153 \| 9628 \| 9609 \| 185 \| 185 \| 0 \| 6876 \| 351 \| 0.051 \| No data \| No data \| No data \| N/A \| N/A \| No \| N/A \| N/A \| \| MK-MalinoSvetiNikole15_2017_H11 \| 960 \| 937 \| 182 \| 173 \| 9 \| 5564 \| 4170 \| 0.75 \| 944 \| 936 \| 4 \| 16 \| 1 \| Yes \| Yes \| G1 \| \| MK-MalinoSvetiNikole15_2017_H12 \| 963 \| 937 \| 1728 \| 1725 \| 3 \| 8308 \| 1897 \| 0.23 \| 949 \| 937 \| 1075 \| 14 \| 0 \| Yes \| Yes \| G1 \| \| MK-MalinoSvetiNikole15_2017_H14 \| 907 \| 863 \| 324 \| 324 \| 0 \| 1568 \| 432 \| 0.28 \| No data \| No data \| No data \| N/A \| N/A \| No \| N/A \| N/A \| \| MK-MalinoSvetiNikole15_2017_H46 \| 964 \| 937 \| 3024 \| 3022 \| 2 \| 12212 \| 3188 \| 0.26 \| 949 \| 937 \| 1795 \| 15 \| 0 \| Yes \| Yes \| G1 \| \| MK-MalinoSvetiNikole15_2017_H47 \| 904 \| 865 \| 278 \| 278 \| 0 \| 7305 \| 384 \| 0.053 \| No data \| No data \| No data \| N/A \| N/A \| No \| N/A \| N/A \| \| MK-MalinoSvetiNikole15_2017_P \| 907 \| 863 \| 902 \| 902 \| 0 \| 3334 \| 1074 \| 0.32 \| 949 \| 937 \| 265 \| 42 \| 74 \| Yes \| Yes \| G1 \| \| MK-MalinoSvetiNikole16_2017_P \| 959 \| 937 \| 13 \| 8 \| 5 \| 8973 \| 468 \| 0.052 \| No data \| No data \| No data \| N/A \| N/A \| No \| N/A \| N/A \| \| UK-SandHutton43_2017_P \| 820 \| 501 \| 44 \| 44 \| 0 \| 1970 \| 227 \| 0.12 \| 821 \| 807 \| 346 \| 1 \| 306 \| Yes \| Yes \| G1 \| \| UK-SandHutton43_2017_P \| 1405 \| 1356 \| 132 \| 132 \| 0 \| 4200 \| 469 \| 0.11 \| 1399 \| 1373 \| 72 \| 6 \| 17 \| No \| N/A \| N/A \| \| UK-SandHutton43_2017_P \| 1594 \| 1559 \| 46 \| 0 \| 46 \| 7211 \| 2858 \| 0.4 \| 1622 \| 1596 \| 101 \| 28 \| 37 \| No \| N/A \| N/A \| \| UK-SandHutton43_2017_P \| 1642 \| 1608 \| 832 \| 92 \| 740 \| 8540 \| 4742 \| 0.56 \| 1634 \| 1608 \| 334 \| 8 \| 0 \| Yes \| Yes \| G1 \| \| UK-SandHutton43_2017_P \| 5979 \| 5878 \| 21 \| 21 \| 0 \| 3563 \| 349 \| 0.098 \| 5975 \| 5947 \| 35 \| 4 \| 69 \| No \| Yes \| N/A \| \| UK-SherburnInElmet35_2017_H188 \| 1795 \| 1742 \| 2836 \| 2817 \| 19 \| 17023 \| 3625 \| 0.21 \| 1774 \| 1748 \| 3 \| 21 \| 6 \| Yes \| No \| G2 \| \| UK-SherburnInElmet35_2017_H189 \| 1795 \| 1742 \| 3954 \| 3940 \| 14 \| 16120 \| 4705 \| 0.29 \| 1768 \| 1742 \| 552 \| 27 \| 0 \| Yes \| No \| G2 \| \| UK-SherburnInElmet35_2017_H189 \| 5172 \| 5045 \| 255 \| 254 \| 1 \| 11399 \| 3668 \| 0.32 \| 5628 \| 5600 \| 119 \| 456 \| 555 \| Yes \| No \| G2 \| \| UK-SherburnInElmet35_2017_P \| 1184 \| 908 \| 134 \| 134 \| 0 \| 9033 \| 1819 \| 0.2 \| 855 \| 848 \| 176 \| 329 \| 60 \| No \| N/A \| N/A \| \| UK-SherburnInElmet35_2017_P \| 1777 \| 1742 \| 40 \| 14 \| 26 \| 18981 \| 3893 \| 0.21 \| 1763 \| 1742 \| 155 \| 14 \| 0 \| No \| N/A \| N/A \| \| UK-SherburnInElmet35_2017_P \| 5164 \| 4880 \| 299 \| 299 \| 0 \| 14137 \| 3629 \| 0.26 \| 5130 \| 5107 \| 187 \| 34 \| 227 \| yes \| No \| G1 \| \| UK-SherburnInElmet36_2017_H193 \| 974 \| 653 \| 159 \| 159 \| 0 \| 8746 \| 473 \| 0.054 \| 873 \| 859 \| 720 \| 101 \| 206 \| Yes \| No \| G2 \| \| UK-SherburnInElmet36_2017_H193 \| 1574 \| 1546 \| 7155 \| 6877 \| 278 \| 26398 \| 6689 \| 0.25 \| 1572 \| 1546 \| 244 \| 2 \| 0 \| Yes \| No \| G2 \| \| UK-SherburnInElmet36_2017_H193 \| 1794 \| 1751 \| 51 \| 48 \| 3 \| 22921 \| 4837 \| 0.21 \| 1768 \| 1742 \| 245 \| 26 \| 9 \| No \| N/A \| N/A \| \| UK-SherburnInElmet36_2017_P \| 1784 \| 1742 \| 5721 \| 5629 \| 92 \| 20217 \| 6354 \| 0.31 \| 1769 \| 1748 \| 833 \| 15 \| 6 \| Yes \| No \| G1 \| \| UK-SherburnInElmet36_2017_P \| 5210 \| 5124 \| 14 \| 14 \| 0 \| 6317 \| 323 \| 0.051 \| 5130 \| 5107 \| 49 \| 80 \| 17 \| No \| N/A \| N/A \| |
| --- | --- | --- | --- | --- | --- | --- | --- | --- | --- | --- | --- | --- | --- | --- | --- | --- | --- | --- | --- | --- | --- | --- | --- | --- | --- | --- | --- | --- | --- | --- | --- | --- | --- | --- | --- | --- | --- | --- | --- | --- | --- | --- | --- | --- | --- | --- | --- | --- | --- | --- | --- | --- | --- | --- | --- | --- | --- | --- | --- | --- | --- | --- | --- | --- | --- | --- | --- | --- | --- | --- | --- | --- | --- | --- | --- | --- | --- | --- | --- | --- | --- | --- | --- | --- | --- | --- | --- | --- | --- | --- | --- | --- | --- | --- | --- | --- | --- | --- | --- | --- | --- | --- | --- | --- | --- | --- | --- | --- | --- | --- | --- | --- | --- | --- | --- | --- | --- | --- | --- | --- | --- | --- | --- | --- | --- | --- | --- | --- | --- | --- | --- | --- | --- | --- | --- | --- | --- | --- | --- | --- | --- | --- | --- | --- | --- | --- | --- | --- | --- | --- | --- | --- | --- | --- | --- | --- | --- | --- | --- | --- | --- | --- | --- | --- | --- | --- | --- | --- | --- | --- | --- | --- | --- | --- | --- | --- | --- | --- | --- | --- | --- | --- | --- | --- | --- | --- | --- | --- | --- | --- | --- | --- | --- | --- | --- | --- | --- | --- | --- | --- | --- | --- | --- | --- | --- | --- | --- | --- | --- | --- | --- | --- | --- | --- | --- | --- | --- | --- | --- | --- | --- | --- | --- | --- | --- | --- | --- | --- | --- | --- | --- | --- | --- | --- | --- | --- | --- | --- | --- | --- | --- | --- | --- | --- | --- | --- | --- | --- | --- | --- | --- | --- | --- | --- | --- | --- | --- | --- | --- | --- | --- | --- | --- | --- | --- | --- | --- | --- | --- | --- | --- | --- | --- | --- | --- | --- | --- | --- | --- | --- | --- | --- | --- | --- | --- | --- | --- | --- | --- | --- | --- | --- | --- | --- | --- | --- | --- | --- | --- | --- | --- | --- | --- | --- | --- | --- | --- | --- | --- | --- | --- | --- | --- | --- | --- | --- | --- | --- | --- | --- | --- | --- | --- | --- | --- | --- | --- | --- | --- | --- | --- | --- | --- | --- | --- | --- | --- | --- | --- | --- | --- | --- | --- | --- | --- | --- | --- | --- | --- | --- | --- | --- | --- | --- | --- | --- | --- | --- | --- | --- | --- | --- | --- | --- | --- | --- | --- | --- | --- | --- | --- | --- | --- | --- | --- | --- | --- | --- | --- | --- | --- | --- | --- | --- | --- | --- | --- | --- | --- | --- | --- | --- | --- | --- | --- | --- | --- | --- | --- | --- | --- | --- | --- | --- | --- | --- | --- | --- | --- | --- | --- | --- | --- | --- | --- | --- | --- | --- | --- | --- | --- | --- | --- | --- | --- | --- | --- | --- | --- | --- | --- | --- | --- | --- | --- | --- | --- | --- | --- | --- | --- | --- | --- | --- | --- | --- | --- | --- | --- | --- | --- | --- | --- | --- | --- | --- | --- | --- | --- | --- | --- | --- | --- | --- | --- | --- | --- | --- | --- | --- | --- | --- | --- | --- | --- | --- | --- | --- | --- | --- | --- | --- | --- | --- | --- | --- | --- | --- | --- | --- | --- | --- | --- | --- | --- | --- | --- | --- | --- | --- | --- | --- | --- | --- | --- | --- | --- | --- | --- | --- | --- | --- | --- | --- | --- | --- | --- | --- | --- | --- | --- | --- | --- | --- | --- | --- | --- | --- | --- | --- | --- | --- | --- | --- | --- | --- | --- | --- | --- | --- | --- | --- | --- | --- | --- | --- | --- | --- | --- | --- | --- | --- | --- | --- | --- | --- | --- | --- | --- | --- | --- | --- | --- | --- | --- | --- | --- | --- | --- | --- | --- | --- | --- | --- | --- | --- | --- | --- | --- | --- | --- | --- | --- | --- | --- | --- | --- | --- | --- | --- | --- | --- | --- | --- | --- | --- | --- | --- | --- | --- | --- | --- | --- | --- | --- | --- | --- | --- | --- | --- | --- | --- | --- | --- | --- | --- | --- | --- | --- | --- | --- | --- | --- | --- | --- | --- | --- | --- | --- | --- | --- | --- | --- | --- | --- | --- | --- | --- | --- | --- | --- | --- | --- | --- | --- | --- | --- | --- | --- | --- | --- | --- | --- | --- | --- | --- | --- | --- | --- | --- | --- | --- | --- | --- | --- | --- | --- | --- | --- | --- | --- | --- | --- | --- | --- | --- | --- | --- | --- | --- | --- | --- | --- | --- | --- | --- | --- | --- | --- | --- | --- | --- | --- | --- | --- | --- | --- | --- | --- | --- | --- | --- | --- | --- | --- | --- | --- | --- | --- | --- | --- | --- | --- | --- | --- | --- | --- | --- | --- | --- | --- | --- | --- | --- | --- | --- | --- | --- | --- | --- | --- | --- | --- | --- | --- | --- | --- | --- | --- | --- | --- | --- | --- | --- | --- | --- | --- | --- | --- | --- | --- | --- | --- | --- | --- | --- | --- | --- | --- | --- | --- | --- | --- | --- | --- | --- | --- | --- | --- | --- | --- | --- | --- | --- | --- | --- | --- | --- | --- | --- | --- | --- | --- | --- | --- | --- | --- | --- | --- | --- | --- | --- | --- | --- | --- | --- | --- | --- | --- | --- | --- | --- | --- | --- | --- | --- | --- | --- | --- | --- | --- | --- | --- | --- | --- | --- | --- | --- | --- | --- | --- | --- | --- | --- | --- | --- | --- | --- | --- | --- | --- | --- | --- | --- | --- | --- | --- | --- | --- | --- | --- | --- | --- | --- | --- | --- | --- | --- | --- | --- | --- | --- | --- | --- | --- | --- | --- | --- | --- | --- | --- | --- | --- | --- | --- | --- | --- | --- | --- | --- | --- | --- | --- | --- | --- | --- | --- | --- | --- | --- | --- | --- | --- | --- | --- | --- | --- | --- | --- | --- | --- | --- | --- | --- | --- | --- | --- | --- | --- | --- | --- | --- | --- | --- | --- | --- | --- | --- | --- | --- | --- | --- | --- | --- | --- | --- | --- | --- | --- | --- | --- | --- | --- | --- | --- | --- | --- | --- | --- | --- | --- | --- | --- | --- | --- | --- | --- | --- | --- | --- | --- | --- | --- | --- | --- | --- | --- | --- | --- | --- | --- | --- | --- | --- | --- | --- | --- | --- | --- | --- | --- | --- | --- | --- | --- | --- | --- | --- | --- | --- | --- | --- | --- | --- | --- | --- | --- | --- | --- | --- | --- | --- | --- | --- | --- | --- | --- | --- | --- | --- | --- | --- | --- | --- | --- | --- | --- | --- | --- | --- | --- | --- | --- | --- | --- | --- | --- | --- | --- | --- | --- | --- | --- | --- | --- | --- | --- | --- | --- | --- | --- | --- | --- | --- | --- | --- | --- | --- | --- | --- | --- | --- | --- | --- | --- | --- | --- | --- | --- | --- | --- | --- | --- | --- | --- | --- | --- | --- | --- | --- | --- | --- | --- | --- | --- | --- | --- | --- | --- | --- | --- | --- | --- | --- | --- | --- | --- | --- | --- | --- | --- | --- | --- | --- | --- | --- | --- | --- | --- | --- | --- | --- | --- | --- | --- | --- | --- | --- | --- | --- | --- | --- | --- | --- | --- | --- | --- | --- | --- | --- | --- | --- | --- | --- | --- | --- | --- | --- | --- | --- | --- | --- | --- | --- | --- | --- | --- | --- | --- | --- | --- | --- | --- | --- | --- | --- | --- | --- | --- | --- | --- | --- | --- | --- | --- | --- | --- | --- | --- | --- | --- | --- | --- | --- | --- | --- | --- | --- | --- | --- | --- | --- | --- | --- | --- | --- | --- | --- | --- | --- | --- | --- | --- | --- | --- | --- | --- | --- | --- | --- | --- | --- | --- | --- | --- | --- | --- | --- | --- | --- | --- | --- | --- | --- | --- | --- | --- | --- | --- | --- | --- | --- | --- | --- | --- | --- | --- | --- | --- | --- | --- | --- | --- | --- | --- | --- | --- | --- | --- | --- | --- | --- | --- | --- | --- | --- | --- | --- | --- | --- | --- | --- | --- | --- | --- | --- | --- | --- | --- | --- | --- | --- | --- | --- | --- | --- | --- | --- | --- | --- | --- | --- | --- | --- | --- | --- | --- | --- | --- | --- | --- | --- | --- | --- | --- | --- | --- | --- | --- | --- | --- | --- | --- | --- | --- | --- | --- | --- | --- | --- | --- | --- | --- | --- | --- | --- | --- | --- | --- | --- | --- | --- | --- | --- | --- | --- | --- | --- | --- | --- | --- | --- | --- | --- | --- | --- | --- | --- | --- | --- | --- | --- | --- | --- | --- | --- | --- | --- | --- | --- | --- | --- | --- | --- | --- | --- | --- | --- | --- | --- | --- | --- | --- | --- | --- | --- | --- | --- | --- | --- | --- | --- | --- | --- | --- | --- | --- | --- | --- | --- | --- | --- | --- | --- | --- | --- | --- | --- | --- | --- | --- | --- | --- | --- | --- | --- | --- | --- | --- | --- | --- | --- | --- | --- | --- | --- | --- | --- | --- | --- | --- | --- | --- | --- | --- | --- | --- | --- | --- | --- | --- | --- | --- | --- | --- | --- | --- | --- | --- | --- | --- | --- | --- | --- | --- | --- | --- | --- | --- | --- | --- | --- | --- | --- | --- | --- | --- | --- | --- | --- | --- | --- | --- | --- | --- | --- | --- | --- | --- | --- | --- | --- | --- | --- | --- | --- | --- | --- | --- | --- | --- | --- | --- | --- | --- | --- | --- | --- | --- | --- | --- | --- | --- | --- | --- | --- | --- | --- | --- | --- | --- | --- | --- | --- | --- | --- | --- | --- | --- | --- | --- | --- |

1: Contig with a portion of its sequence aligning to one DWV genotype and the other portion aligning to another genotype
2 (No data): ViReMa did not generate any result for the sample
3 (N/A): Not available
4 (Not found): The junction was not detected by ViReMa

| **Supplementary Table S3.** DWV-A, DWV-B  or DWV-recombinant genomes assembled from honey bee polled or single bee head of honey bees. | | | | | | | | | | | | | |
| --- | --- | --- | --- | --- | --- | --- | --- | --- | --- | --- | --- | --- | --- |
| **Sample indentification** | **DWV-A** | | | **DWV-B** | | | | **DWV-recombinant** | | | | | |
|  | **Detection ^1^** | **Genome code ^2^** | | **Detection ^1^** | | | **Genome code ^2^** | **Detection ^1^** | | | | | **Genome code ^2^** |
| DE-HohenNeuendorf_2015_H3 | - | ND | | ++ | | | G1 | ++ | | | | | G2 |
| DE-HohenNeuendorf_2015_H6 | - | ND | | ++ | | | G1 | ++ | | | | | G2 |
| ES-Arbeiza21_2017_P | +/- | ND | | ++ | | | G1 | ++ | | | | | ND |
| ES-Arbeiza22_2017_H34 | - | ND | | ++ | | | G1 | ++ | | | | | G2 |
| ES-Arbeiza22_2017_H35 | ++ | G2 | | ++ | | | G1 | - | | | | | ND |
| ES-Arbeiza22_2017_H62 | - | ND | | ++ | | | G1 | ++ | | | | | G2 and G3 |
| ES-Arbeiza22_2017_H67 | - | ND | | +/- | | | ND | + | | | | | G1 |
| ES-Arbeiza22_2017_P | - | ND | | +++ | | | G1 | ++ | | | | | G2 |
| ES-Fraisoro18_2017_P | - | ND | | + | | | G1 | +/- | | | | | ND |
| ES-Opakua(Araba)20_2017_P | - | ND | | ++ | | | G1 | ++ | | | | | G2 |
| FR-Caumont10_2017_P | - | ND | | ++ | | | G2 | ++ | | | | | G1 |
| FR-Caumont7_2017_P | - | ND | | +/- | | | ND | ++ | | | | | G1 |
| FR-Caumont8_2017_P | - | ND | | - | | | ND | ++ | | | | | G1 |
| FR-Caumont9_2017_P | +/- | ND | | +/- | | | ND | ++ | | | | | G1 |
| FR-Sophia1_2017_P | - | ND | | +++ | | | G1 | +/- | | | | | ND |
| FR-Sophia3_2017_P | +/- | ND | | +++ | | | G1 | +++ | | | | | G2 |
| FR-Caumont6_2017_H111 | - | ND | | +/- | | | ND | +++ | | | | | G1 |
| FR-Caumont6_2017_H113 | - | ND | | - | | | ND | +++ | | | | | G1 |
| FR-Caumont6_2017_H78 | - | ND | | - | | | ND | ++ | | | | | G1 |
| FR-Sophia2_2017_H173 | - | ND | | ++ | | | G1 | - | | | | | ND |
| FR-Sophia2_2017_H174 | - | ND | | ++ | | | G1 | - | | | | | ND |
| GR-DomenicoLavisa11_2017_P | - | ND | | +++ | | | G1 | ++ | | | | | G2 |
| GR-DomenicoLavisa12_2017_H148 | - | ND | | + | | | G1 | +/- | | | | | ND |
| GR-DomenicoLavisa12_2017_H149 | - | ND | | +++ | | | G1 | - | | | | | ND |
| HR-HrvatskaKostajnica32_2017_P | - | ND | | ++ | | | G1 | - | | | | | ND |
| HR-HrvatskaKostajnica50_2017_H121 | ++ | G1 | | +/- | | | ND | - | | | | | ND |
| HR-HrvatskaKostajnica50_2017_H122 | - | ND | | +++ | | | G1 | - | | | | | ND |
| HR-HrvatskaKostajnica50_2017_H128 | ++ | G1 | | ++ | | | G2 | - | | | | | ND |
| HR-HrvatskaKostajnica50_2017_H83 | ++ | G1 | | + | | | G2 | - | | | | | ND |
| HR-HrvatskaKostajnica50_2017_H88 | +/- | ND | | ++ | | | G1 | - | | | | | ND |
| HR-HrvatskaKostajnica50_2017_P | - | ND | | +/- | | | G1 | - | | | | | ND |
| HR-Sisak39_2017_P | ++ | G2 | | ++ | | | G1 | - | | | | | ND |
| HR-Sisak48_2017_P | + | G2 | | + | | | G1 | - | | | | | ND |
| IT_Palermo4_2017_P | ++ | G1 | | ++ | | | G2 | - | | | | | ND |
| IT_Palermo5_2017_P | ++ | G1 | | +/- | | | ND | - | | | | | ND |
| IT-Napoli59_2017_H162 | ++ | G1 | | - | | | ND | - | | | | | ND |
| IT-Napoli59_2017_H163 | + | G1 | | - | | | ND | - | | | | | ND |
| IT-Napoli59_2017_P | + | G1 | | +/- | | | ND | - | | | | | ND |
| IT-Palermo4_2017_H142 | ++ | G1 | | - | | | ND | - | | | | | ND |
| IT-Palermo4_2017_H143 | +++ | G1 | | - | | | ND | - | | | | | ND |
| IT-Palermo51_2017_P | + | G1 | | + | | | G2 | + | | | | | G3 |
| IT-Palermo52_2017_P | ++ | G1 | | ++ | | | G3 | ++ | | | | | ND |
| MD-GhidighiciChisinau23_2017_H153 | ++ | G1 | | - | | | ND | - | | | | | ND |
| MD-GhidighiciChisinau23_2017_H156 | ++ | G1 | | - | | | ND | - | | | | | ND |
| MD-GhidighiciChisinau23_2017_P | ++ | G1 | | - | | | ND | - | | | | | ND |
| MD-GhidighiciChisinau25_2017_P | ++ | G1 | | - | | | ND | - | | | | | ND |
| MD-GhidighiciChisinau26_2017_P | +++ | G1 | | +/- | | | ND | - | | | | | ND |
| MK-MalinoSvetiNikole15_2017_H11 | - | ND | | +/- | | | ND | ++ | | | | | G1 |
| MK-MalinoSvetiNikole15_2017_H12 | + | G3 | | ++ | | | G2 | ++ | | | | | G1 |
| MK-MalinoSvetiNikole15_2017_H14 | + | G2 | | ++ | | | G1 | + | | | | | ND |
| MK-MalinoSvetiNikole15_2017_H46 | - | ND | | ++ | | | G2 | ++ | | | | | G1 |
| MK-MalinoSvetiNikole15_2017_H47 | + | G2 | | +++ | | | G1 | - | | | | | ND |
| MK-MalinoSvetiNikole15_2017_P | +/- | ND | | +++ | | | G2 | +++ | | | | | G1 |
| MK-MalinoSvetiNikole16_2017_P | + | G2 | | ++ | | | G1 | - | | | | | ND |
| MK-SDihovoBitola53_2017_P | +/- | ND | | + | | | G1 | - | | | | | ND |
| RO-Bucharest40_2017_P | ++ | G1 | | +/- | | | ND | - | | | | | ND |
| RO-TarguMures31_2017_H23 | ++ | G1 | | - | | | ND | - | | | | | ND |
| RO-TarguMures31_2017_H25 | ++ | G1 | | - | | | ND | - | | | | | ND |
| RO-TarguMures31_2017_H26 | ++ | G1 | | - | | | ND | - | | | | | ND |
| RO-TarguMures31_2017_H28 | ++ | G1 | | +/- | | | ND | - | | | | | ND |
| RO-TarguMures31_2017_H53 | ++ | G1 | | +/- | | | ND | - | | | | | ND |
| RO-TarguMures31_2017_H60 | ++ | G1 | | + | | | G2 | - | | | | | ND |
| RO-TarguMures31_2017_P | + | G1 | | +/- | | | ND | - | | | | | ND |
| RS-Belgrade29_2017_H133 | - | ND | | +/- | | | G1 | - | | | | | ND |
| RS-Belgrade30_2017_P | ++ | G1 | | +/- | | | ND | - | | | | | ND |
| UK-Flaxton37_2017_P | - | ND | | ++ | | | G1 | - | | | | | ND |
| UK-Flaxton38_2017_P | - | ND | | ++ | | | G1 | - | | | | | ND |
| UK-SandHutton43_2017_P | +/- | ND | | ++ | | | G2 | ++ | | | | | G1 |
| UK-SherburnInElmet35_2017_H188 | - | ND | | ++ | | | G1 | ++ | | | | | G2 |
| UK-SherburnInElmet35_2017_H189 | - | ND | | ++ | | | G1 | ++ | | | | | G2 |
| UK-SherburnInElmet35_2017_P | +/- | ND | | ++ | | | G2 | +++ | | | | | G1 |
| UK-SherburnInElmet36_2017_H193 | - | ND | | ++ | | | G1 | ++ | | | | | G2 |
| UK-SherburnInElmet36_2017_H194 | - | ND | | +++ | | | G1 | - | | | | | ND |
| UK-SherburnInElmet36_2017_P | - | ND | | ++ | | | G2 | ++ | | | | | G1 |
|  |  | |  | | |  | | |  | |  |  |  |
| **1:** Symbol | Coverage depth | | | | Definition | | | | |  | |  |  |
| - | 0-1 | | | | not significant | | | | |  | |  |  |
| +/- | 1-2 | | | | suspected | | | | |  | |  |  |
| + | 2-3 | | | | detected | | | | |  | |  |  |
| ++ | 3-4 | | | | detected | | | | |  | |  |  |
| +++ | >4 | | | | detected | | | | |  | |  |  |
| **2.** ND: no data |  |  |  |  |  |  |  |  |  |  |  |  |  |

| **Supplementary Table S4.** GenBank accession number of the DWV-A, DWV-B or DWV-recombinant genomes. | | | |
| --- | --- | --- | --- |
| **Sample indentification** | **BioSample accession number** | **Genome code** | **GenBank accession number** |
|  |  |  |  |
| DE-HohenNeuendorf_2015_H3 | SAMN38976193 | G1 | PP437882 |
|  |  | G2 | PP437883 |
| DE-HohenNeuendorf_2015_H6 | SAMN38976199 | G1 | PP437884 |
|  |  | G2 | PP437885 |
| ES-Arbeiza21_2017_P | SAMN38976129 | G1 | PP437886 |
| ES-Arbeiza22_2017_H34 | SAMN38976194 | G1 | PP437887 |
|  |  | G2 | PP437888 |
| ES-Arbeiza22_2017_H35 | SAMN38976195 | G1 | PP437889 |
|  |  | G2 | PP437890 |
| ES-Arbeiza22_2017_H62 | SAMN38976201 | G1 | PP437891 |
|  |  | G2 | PP437892 |
|  |  | G3 | PP437893 |
| ES-Arbeiza22_2017_H67 | SAMN38976202 | G1 | PP437894 |
| ES-Arbeiza22_2017_P | SAMN38976130 | G1 | PP437895 |
|  |  | G2 | PP437896 |
| ES-Fraisoro18_2017_P | SAMN38976127 | G1 | PP437897 |
| ES-Opakua(Araba)20_2017_P | SAMN38976128 | G1 | PP437898 |
|  |  | G2 | PP437899 |
| FR_Caumont10_2017_P | SAMN38976121 | G1 | PP437900 |
|  |  | G2 | PP437901 |
| FR_Caumont7_2017_P | SAMN38976118 | G1 | PP437902 |
| FR_Caumont8_2017_P | SAMN38976119 | G1 | PP437903 |
| FR_Caumont9_2017_P | SAMN38976120 | G1 | PP437904 |
| FR_Sophia1_2017_P | SAMN38976114 | G1 | PP437905 |
| FR_Sophia3_2017_P | SAMN38976115 | G1 | PP437906 |
|  |  | G2 | PP437907 |
| FR-Caumont6_2017_H111 | SAMN38976166 | G1 | PP437908 |
| FR-Caumont6_2017_H113 | SAMN38976167 | G1 | PP437909 |
| FR-Caumont6_2017_H78 | SAMN38976204 | G1 | PP437910 |
| FR-Sophia2_2017_H173 | SAMN38976183 | G1 | PP437911 |
| FR-Sophia2_2017_H174 | SAMN38976184 | G1 | PP437912 |
| GR-DomenicoLavisa11_2017_P | SAMN38976122 | G1 | PP437913 |
|  |  | G2 | PP437914 |
| GR-DomenicoLavisa12_2017_H148 | SAMN38976177 | G1 | PP437915 |
| GR-DomenicoLavisa12_2017_H149 | SAMN38976178 | G1 | PP437916 |
| HR-HrvatskaKostajnica32_2017_P | SAMN38976139 | G1 | PP437917 |
| HR-HrvatskaKostajnica50_2017_H121 | SAMN38976169 | G1 | PP437918 |
| HR-HrvatskaKostajnica50_2017_H122 | SAMN38976170 | G1 | PP437919 |
| HR-HrvatskaKostajnica50_2017_H128 | SAMN38976171 | G1 | PP437920 |
|  |  | G2 | PP437921 |
| HR-HrvatskaKostajnica50_2017_H83 | SAMN38976205 | G1 | PP437922 |
|  |  | G2 | PP437923 |
| HR-HrvatskaKostajnica50_2017_H88 | SAMN38976206 | G1 | PP437924 |
| HR-HrvatskaKostajnica50_2017_P | SAMN38976151 | G1 | PP437925 |
| HR-Sisak39_2017_P | SAMN38976145 | G1 | PP437926 |
|  |  | G2 | PP437927 |
| HR-Sisak48_2017_P | SAMN38976150 | G1 | PP437928 |
|  |  | G2 | PP437929 |
| IT_Palermo4_2017_P | SAMN38976116 | G1 | PP437930 |
|  |  | G2 | PP437931 |
| IT_Palermo5_2017_P | SAMN38976117 | G1 | PP437932 |
| IT-Napoli59_2017_H162 | SAMN38976181 | G1 | PP437933 |
| IT-Napoli59_2017_H163 | SAMN38976182 | G1 | PP437934 |
| IT-Napoli59_2017_P | SAMN38976158 | G1 | PP437935 |
| IT-Palermo4_2017_H142 | SAMN38976175 | G1 | PP437936 |
| IT-Palermo4_2017_H143 | SAMN38976176 | G1 | PP437937 |
| IT-Palermo51_2017_P | SAMN38976152 | G1 | PP437938 |
|  |  | G2 | PP437939 |
|  |  | G3 | PP437940 |
| IT-Palermo52_2017_P | SAMN38976153 | G1 | PP437941 |
|  |  | G3 | PP437943 |
| MD-GhidighiciChisinau23_2017_H153 | SAMN38976179 | G1 | PP437944 |
| MD-GhidighiciChisinau23_2017_H156 | SAMN38976180 | G1 | PP437945 |
| MD-GhidighiciChisinau23_2017_P | SAMN38976131 | G1 | PP437946 |
| MD-GhidighiciChisinau25_2017_P | SAMN38976132 | G1 | PP437947 |
| MD-GhidighiciChisinau26_2017_P | SAMN38976133 | G1 | PP437948 |
| MK-MalinoSvetiNikole15_2017_H11 | SAMN38976165 | G1 | PP437949 |
| MK-MalinoSvetiNikole15_2017_H12 | SAMN38976168 | G1 | PP437950 |
|  |  | G2 | PP437951 |
|  |  | G3 | PP437952 |
| MK-MalinoSvetiNikole15_2017_H14 | SAMN38976174 | G1 | PP437953 |
|  |  | G2 | PP437954 |
| MK-MalinoSvetiNikole15_2017_H46 | SAMN38976196 | G1 | PP437955 |
|  |  | G2 | PP437956 |
| MK-MalinoSvetiNikole15_2017_H47 | SAMN38976197 | G1 | PP437957 |
|  |  | G2 | PP437958 |
| MK-MalinoSvetiNikole15_2017_P | SAMN38976125 | G1 | PP437959 |
|  |  | G2 | PP437960 |
| MK-MalinoSvetiNikole16_2017_P | SAMN38976126 | G1 | PP437961 |
|  |  | G2 | PP437962 |
| MK-SDihovoBitola53_2017_P | SAMN38976154 | G1 | PP437963 |
| RO-Bucharest40_2017_P | SAMN38976146 | G1 | PP437964 |
| RO-TarguMures31_2017_H23 | SAMN38976189 | G1 | PP437965 |
| RO-TarguMures31_2017_H25 | SAMN38976190 | G1 | PP437966 |
| RO-TarguMures31_2017_H26 | SAMN38976191 | G1 | PP437967 |
| RO-TarguMures31_2017_H28 | SAMN38976192 | G1 | PP437968 |
| RO-TarguMures31_2017_H53 | SAMN38976198 | G1 | PP437969 |
| RO-TarguMures31_2017_H60 | SAMN38976200 | G1 | PP437970 |
|  |  | G2 | PP437971 |
| RO-TarguMures31_2017_P | SAMN38976138 | G1 | PP437972 |
| RS-Belgrade29_2017_H133 | SAMN38976172 | G1 | PP437973 |
| RS-Belgrade30_2017_P | SAMN38976137 | G1 | PP437974 |
| UK-Flaxton37_2017_P | SAMN38976143 | G1 | PP437975 |
| UK-Flaxton38_2017_P | SAMN38976144 | G1 | PP437976 |
| UK-SandHutton43_2017_P | SAMN38976148 | G1 | PP437977 |
|  |  | G2 | PP437978 |
| UK-SherburnInElmet35_2017_H188 | SAMN38976185 | G1 | PP437979 |
|  |  | G2 | PP437980 |
| UK-SherburnInElmet35_2017_H189 | SAMN38976186 | G1 | PP437981 |
|  |  | G2 | PP437982 |
| UK-SherburnInElmet35_2017_P | SAMN38976141 | G1 | PP437983 |
|  |  | G2 | PP437984 |
| UK-SherburnInElmet36_2017_H193 | SAMN38976187 | G1 | PP437985 |
|  |  | G2 | PP437986 |
| UK-SherburnInElmet36_2017_H194 | SAMN38976188 | G1 | PP437987 |
| UK-SherburnInElmet36_2017_P | SAMN38976142 | G1 | PP437988 |
|  |  | G2 | PP437989 |

**A**


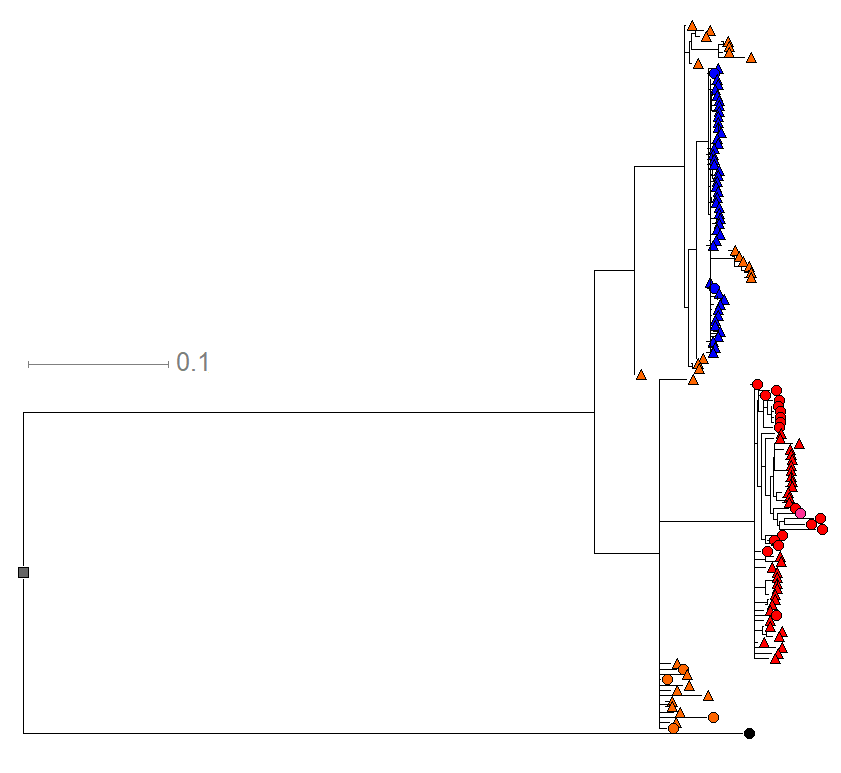


**B**


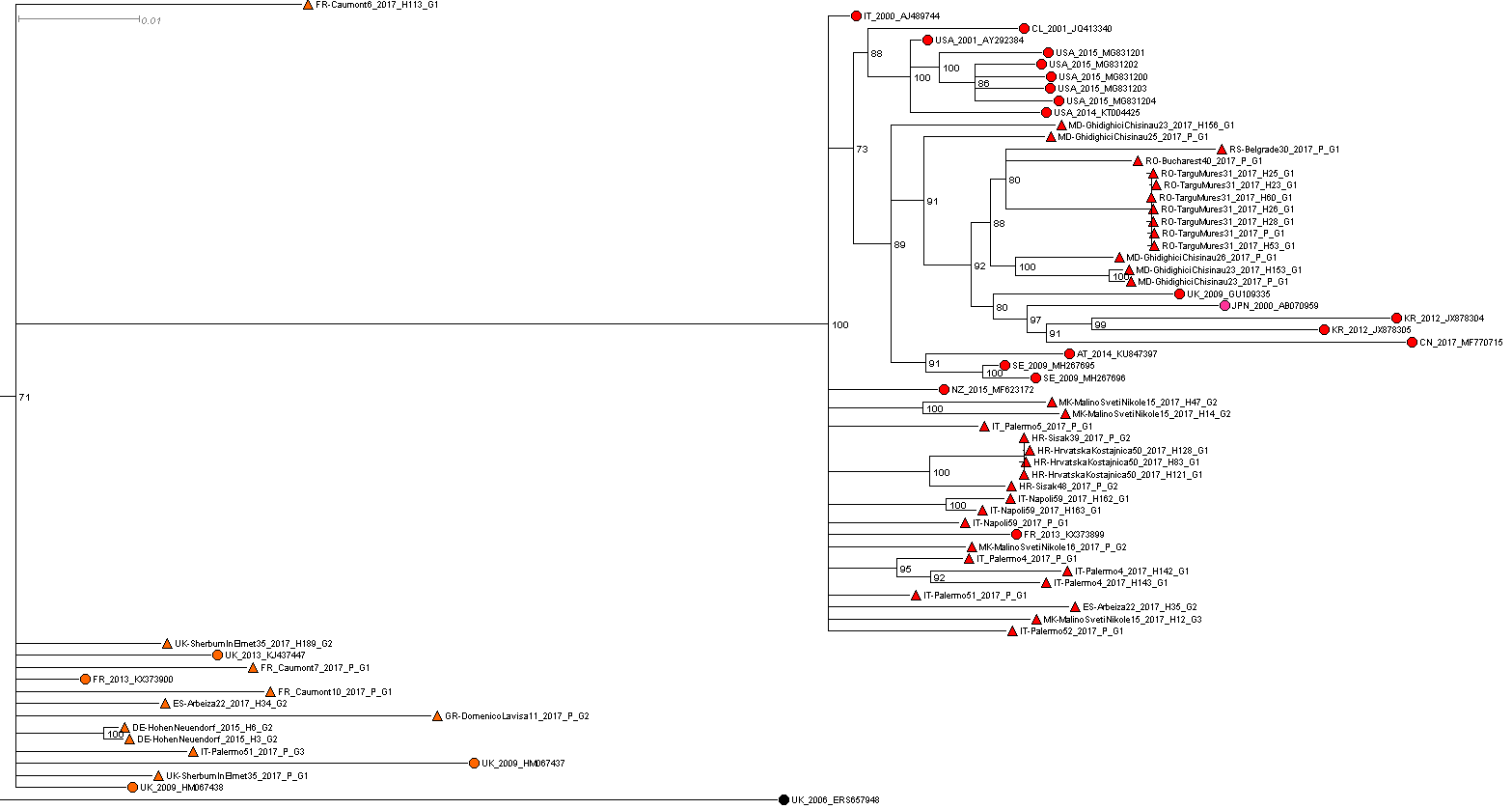


**C**

**
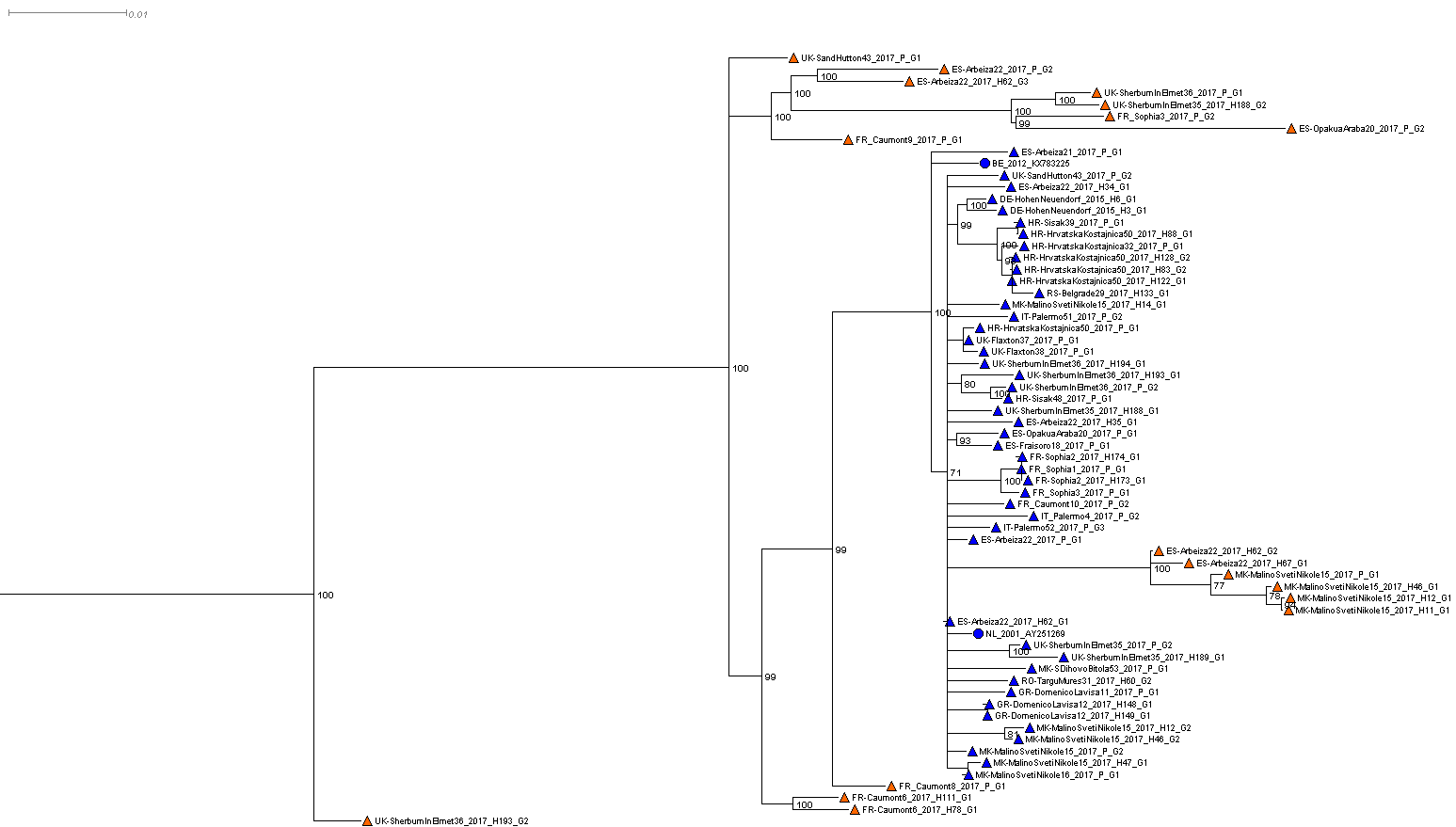
**

**Supplementary Figure S5.** Maximum likelihood phylogenetic tree of DWV genomes. **A**: The tree was rooted at midpoint. Maximum likelihood trees were constructed using RaxML with the general time-reversible (GTR+G+I) substitution model. The scale bar represents the unit for the expected number of substitutions per site. Red, blue, orange, pink and black symbols correspond to DWV-A, DWV-B, DWV-recombinant, Kakugo and DWV-C virus genomes, respectively. Ovals and triangles represent reference and reconstructed genomes, respectively. **B** and **C** correspond to a zoom on the upper and lower part of the tree. Numbers at the nodes indicate supporting bootstrap values (in %) for 600 resampled datasets, and only values greater than 70% are shown. Sample names are written in the following format: COUNTRYCODE_ApiaryColonyID_Year_Type_GX. The type could be either P for pool of honeybees or H for single honeybee head. GX corresponds to the number of the reconstructed genome in the given sample, knowing that several genomes were characterised in some samples. Names of reference genomes are written as follows: COUNTRYCODE_Year_AccessionNumber.

**Supplementary Figure S6.** Phylogenetic trees of DWV partial genome sequences. **A.** Analysis of VP1-VP3 gene sequences. **B.** analysis of *3C-protease*-*RNA-dependent RNA-polymerase* gene sequences. The scale bars represent the unit for the expected number of substitutions per site. Red, blue or orange triangles correspond to the reconstructed genomes of DWV-A, DWV-B or DWV-recombinant, respectively. Branch support values are indicated in red color.

| **Supplementary Table S5.** Honey bee colony and sample information ^1,2^. | | | | | | | | | | | | | | | | | | | | | | |
| --- | --- | --- | --- | --- | --- | --- | --- | --- | --- | --- | --- | --- | --- | --- | --- | --- | --- | --- | --- | --- | --- | --- |
| **Colony ID** | **Country** | **City/Region** | **Latitude** | **Longitude** | **Varroa status** | **Material provided** | **Year  of sampling** | **Month  of sampling** | **Honey bee subspecies** | **Varroa treatment  (during year of sampling)** | **Varroa infestion level (per 100 bees)** | **Pool sample ID** | **Number of foragers**  **used in pool-analysis** | **Single head sample ID** | **Number of bees used in individual analysis with or without wing deformities** | | **Single varroa sample ID** | **Number of individual varroa mites analyzed** | **Pool selected for sequencing** | **Single bee head selected for sequencing** | **provider name** | **Institution** |
| AT-LunzKIR56 | Austria | Lunz | 47.86 N | 15.03 E | infested | RNA (10 bee heads from upper super) | 2010 | 9 | *carnica* | none | 11 | KIR56 | 10 (bee heads) | ND | ND | | ND | ND | ND | ND | Marina Meixner | Landesbetrieb Landwirtschaft Hessen (LLH), Germany |
| AT-LunzKIR57 | Austria | Lunz | 47.86 N | 15.03 E | infested | RNA (10 bee heads from upper super) | 2010 | 9 | *carnica* | none | 15.7 | KIR57 | 10 (bee heads) | ND | ND | | ND | ND | ND | ND | Marina Meixner | Landesbetrieb Landwirtschaft Hessen (LLH), Germany |
| AT-LunzKIR58 | Austria | Lunz | 47.86 N | 15.03 E | infested | RNA (10 bee heads from upper super) | 2010 | 9 | *carnica* | none | 16.3 | KIR58 | 10 (bee heads) | ND | ND | | ND | ND | ND | ND | Marina Meixner | Landesbetrieb Landwirtschaft Hessen (LLH), Germany |
| AT-LunzKIR59 | Austria | Lunz | 47.86 N | 15.03 E | infested | RNA (10 bee heads from upper super) | 2010 | 9 | *macedonica* | none | 1.8 | KIR59 | 10 (bee heads) | ND | ND | | ND | ND | ND | ND | Marina Meixner | Landesbetrieb Landwirtschaft Hessen (LLH), Germany |
| AT-LunzKIR60 | Austria | Lunz | 47.86 N | 15.03 E | infested | RNA (10 bee heads from upper super) | 2010 | 9 | *carnica* | none | 14.9 | KIR60 | 10 (bee heads) | ND | ND | | ND | ND | ND | ND | Marina Meixner | Landesbetrieb Landwirtschaft Hessen (LLH), Germany |
| BG-DimovciKIR27 | Bulgaria | Dimovci | 42.01 N | 25.01 E | infested | RNA (10 bee heads from upper super) | 2011 | 3 | *macedonica* | none | 31 | KIR27 | 10 (bee heads) | ND | ND | | ND | ND | ND | ND | Marina Meixner | Landesbetrieb Landwirtschaft Hessen (LLH), Germany |
| BG-DimovciKIR28 | Bulgaria | Dimovci | 42.01 N | 25.01 E | infested | RNA (10 bee heads from upper super) | 2011 | 3 | *macedonica* | none | 51 | KIR28 | 10 (bee heads) | ND | ND | | ND | ND | ND | ND | Marina Meixner | Landesbetrieb Landwirtschaft Hessen (LLH), Germany |
| BG-PlovdivKIR29 | Bulgaria | Plovdiv | 42.14 N | 24.75 E | infested | RNA (10 bee heads from upper super) | 2011 | 3 | *macedonica* | none | 19 | KIR29 | 10 (bee heads) | ND | ND | | ND | ND | ND | ND | Marina Meixner | Landesbetrieb Landwirtschaft Hessen (LLH), Germany |
| BG-PlovdivKIR30 | Bulgaria | Plovdiv | 42.14 N | 24.75 E | infested | RNA (10 bee heads from upper super) | 2011 | 3 | *macedonica* | none | 6 | KIR30 | 10 (bee heads) | ND | ND | | ND | ND | ND | ND | Marina Meixner | Landesbetrieb Landwirtschaft Hessen (LLH), Germany |
| BG-VinicaKIR26 | Bulgaria | Vinica | 42.01 N | 25.01 E | infested | RNA (10 bee heads from upper super) | 2011 | 3 | *macedonica* | none | 33 | KIR26 | 10 (bee heads) | ND | ND | | ND | ND | ND | ND | Marina Meixner | Landesbetrieb Landwirtschaft Hessen (LLH), Germany |
| DE-HohenNeuendorf | Germany | Hohen Neuendorf | 52.67 N | 13.28 E | infested | deformed wing bees, varroa | 2015 | 9 | *carnica* | none | no data | ND | ND | H1-H10 | 10 deformed | | V55-V60 | 5 | ND | H3, H6 | Elke Genersch/  Sebastian Gisder | Länderinstitut für Bienenkunde e.V. (LIB), Germany |
| DE-KIR03 | Germany | no data | 50.46 N | 9.09 E | infested | RNA (10 bee heads from upper super) | 2010 | 10 | *carnica* | oxalic acid and formic acid | 2.69 | KIR3 | 10 (bee heads) | ND | ND | | ND | ND | ND | ND | Marina Meixner | Landesbetrieb Landwirtschaft Hessen (LLH), Germany |
| DE-KIR04 | Germany | no data | 49.72 N | 9.04 E | infested | RNA (10 bee heads from upper super) | 2010 | 10 | *carnica* | oxalic acid and thymol | 0 | KIR4 | 10 (bee heads) | ND | ND | | ND | ND | ND | ND | Marina Meixner | Landesbetrieb Landwirtschaft Hessen (LLH), Germany |
| DE-KIR05 | Germany | no data | 50.15 N | 9.14 E | infested | RNA (10 bee heads from upper super) | 2010 | 11 | *carnica* | oxalic acid and formic acid | 0.62 | KIR5 | 10 (bee heads) | ND | ND | | ND | ND | ND | ND | Marina Meixner | Landesbetrieb Landwirtschaft Hessen (LLH), Germany |
| DE-KIR06 | Germany | no data | 51.37 N | 7.13 E | infested | RNA (10 bee heads from upper super) | 2010 | 10 | *carnica* | oxalic acid and formic acid | 0.8 | KIR6 | 10 (bee heads) | ND | ND | | ND | ND | ND | ND | Marina Meixner | Landesbetrieb Landwirtschaft Hessen (LLH), Germany |
| DE-KIR08 | Germany | no data | 51.96 N | 7.41 E | infested | RNA (10 bee heads from upper super) | 2010 | 10 | hybrid buckfast | oxalic acid and formic acid | 0 | KIR8 | 10 (bee heads) | ND | ND | | ND | ND | ND | ND | Marina Meixner | Landesbetrieb Landwirtschaft Hessen (LLH), Germany |
| DE-KIR10 | Germany | no data | no data | no data | infested | RNA (10 bee heads from upper super) | 2010 | 10 | *carnica* | oxalic acid and formic acid | 1.9 | KIR10 | 10 (bee heads) | ND | ND | | ND | ND | ND | ND | Marina Meixner | Landesbetrieb Landwirtschaft Hessen (LLH), Germany |
| DE-KIR11 | Germany | no data | 51.41 N | 6.78 E | infested | RNA (10 bee heads from upper super) | 2010 | 10 | *carnica* | oxalic acid and formic acid | 0 | KIR11 | 10 (bee heads) | ND | ND | | ND | ND | ND | ND | Marina Meixner | Landesbetrieb Landwirtschaft Hessen (LLH), Germany |
| DE-KIR12 | Germany | no data | 50.51 N | 6.28 E | infested | RNA (10 bee heads from upper super) | 2010 | 10 | *carnica* | oxalic acid and formic acid | 1.9 | KIR12 | 10 (bee heads) | ND | ND | | ND | ND | ND | ND | Marina Meixner | Landesbetrieb Landwirtschaft Hessen (LLH), Germany |
| DE-KIR13 | Germany | no data | 50.51 N | 6.71 E | infested | RNA (10 bee heads from upper super) | 2010 | 10 | *carnica* | oxalic acid and formic acid | 1.6 | KIR13 | 10 (bee heads) | ND | ND | | ND | ND | ND | ND | Marina Meixner | Landesbetrieb Landwirtschaft Hessen (LLH), Germany |
| DE-KIR18 | Germany | no data | 48.58 N | 8.88 E | infested | RNA (10 bee heads from upper super) | 2010 | 10 | *carnica* | oxalic acid and removal drone brood | 0 | KIR18 | 10 (bee heads) | ND | ND | | ND | ND | ND | ND | Marina Meixner | Landesbetrieb Landwirtschaft Hessen (LLH), Germany |
| DE-KIR19 | Germany | no data | 48.59 N | 10.16 E | infested | RNA (10 bee heads from upper super) | 2010 | 10 | *carnica* | oxalic acid and removal drone brood, formic acid | 0 | KIR19 | 10 (bee heads) | ND | ND | | ND | ND | ND | ND | Marina Meixner | Landesbetrieb Landwirtschaft Hessen (LLH), Germany |
| DE-KIR23 | Germany | no data | 53.06 N | 8.13 E | infested | RNA (10 bee heads from upper super) | 2010 | 10 | *carnica* | oxalic acid and formic acid | 0.3 | KIR23 | 10 (bee heads) | ND | ND | | ND | ND | ND | ND | Marina Meixner | Landesbetrieb Landwirtschaft Hessen (LLH), Germany |
| DE-KIR24 | Germany | no data | 52.25 N | 10.39 E | infested | RNA (10 bee heads from upper super) | 2010 | 10 | *carnica* | oxalic acid and removal drone brood, lactic acid | 1.8 | KIR24 | 10 (bee heads) | ND | ND | | ND | ND | ND | ND | Marina Meixner | Landesbetrieb Landwirtschaft Hessen (LLH), Germany |
| DE-KIR25 | Germany | no data | 53.02 N | 8.56 E | infested | RNA (10 bee heads from upper super) | 2010 | 10 | *carnica* | oxalic acid and formic acid | 0 | KIR25 | 10 (bee heads) | ND | ND | | ND | ND | ND | ND | Marina Meixner | Landesbetrieb Landwirtschaft Hessen (LLH), Germany |
| DE-MaulbachKIR41 | Germany | Maulbach | 50.73 N | 9.05 E | infested | RNA (10 bee heads from upper super) | 2010 | 10 | *carnica* | none | 28.5 | KIR41 | 10 (bee heads) | ND | ND | | ND | ND | ND | ND | Marina Meixner | Landesbetrieb Landwirtschaft Hessen (LLH), Germany |
| DE-MaulbachKIR42 | Germany | Maulbach | 50.73 N | 9.05 E | infested | RNA (10 bee heads from upper super) | 2010 | 10 | *carnica* | none | 28.9 | KIR42 | 10 (bee heads) | ND | ND | | ND | ND | ND | ND | Marina Meixner | Landesbetrieb Landwirtschaft Hessen (LLH), Germany |
| DE-MaulbachKIR43 | Germany | Maulbach | 50.73 N | 9.05 E | infested | RNA (10 bee heads from upper super) | 2010 | 10 | *carnica* | none | 6.8 | KIR43 | 10 (bee heads) | ND | ND | | ND | ND | ND | ND | Marina Meixner | Landesbetrieb Landwirtschaft Hessen (LLH), Germany |
| DE-MaulbachKIR44 | Germany | Maulbach | 50.73 N | 9.05 E | infested | RNA (10 bee heads from upper super) | 2010 | 10 | *carnica* | none | 12.4 | KIR44 | 10 (bee heads) | ND | ND | | ND | ND | ND | ND | Marina Meixner | Landesbetrieb Landwirtschaft Hessen (LLH), Germany |
| DE-MaulbachKIR45 | Germany | Maulbach | 50.73 N | 9.05 E | infested | RNA (10 bee heads from upper super) | 2010 | 10 | *carnica* | none | 10.4 | KIR45 | 10 (bee heads) | ND | ND | | ND | ND | ND | ND | Marina Meixner | Landesbetrieb Landwirtschaft Hessen (LLH), Germany |
| DE-SchenkenturmKIR51 | Germany | Schenkenturm | 49.82 N | 9.90 E | infested | RNA (10 bee heads from upper super) | 2010 | 10 | *carnica* | none | 23.6 | KIR51 | 10 (bee heads) | ND | ND | | ND | ND | ND | ND | Marina Meixner | Landesbetrieb Landwirtschaft Hessen (LLH), Germany |
| DE-SchenkenturmKIR52 | Germany | Schenkenturm | 49.82 N | 9.90 E | infested | RNA (10 bee heads from upper super) | 2010 | 10 | *carnica* | none | 38.3 | KIR52 | 10 (bee heads) | ND | ND | | ND | ND | ND | ND | Marina Meixner | Landesbetrieb Landwirtschaft Hessen (LLH), Germany |
| DE-SchenkenturmKIR53 | Germany | Schenkenturm | 49.82 N | 9.90 E | infested | RNA (10 bee heads from upper super) | 2010 | 10 | *carnica* | none | 21.7 | KIR53 | 10 (bee heads) | ND | ND | | ND | ND | ND | ND | Marina Meixner | Landesbetrieb Landwirtschaft Hessen (LLH), Germany |
| DE-SchenkenturmKIR54 | Germany | Schenkenturm | 49.82 N | 9.90 E | infested | RNA (10 bee heads from upper super) | 2010 | 10 | *carnica* | none | 27.1 | KIR54 | 10 (bee heads) | ND | ND | | ND | ND | ND | ND | Marina Meixner | Landesbetrieb Landwirtschaft Hessen (LLH), Germany |
| DE-SchenkenturmKIR55 | Germany | Schenkenturm | 49.82 N | 9.90 E | infested | RNA (10 bee heads from upper super) | 2010 | 10 | *carnica* | none | 29.5 | KIR55 | 10 (bee heads) | ND | ND | | ND | ND | ND | ND | Marina Meixner | Landesbetrieb Landwirtschaft Hessen (LLH), Germany |
| ES-Arbeiza21 | Spain | Arbeiza | 42.67 N | 2.07 W | infested | foragers, deformed wing bees, varroa | 2017 | 8 | *iberiensis* | none | 3.9 | 21 | 20 | H177-H185 | 9 deformed | | V31-V35 | 5 | yes | ND | Andone Estonba/  Egoitz Galarza | University of the Basque Country (UPV/EHU), Spain |
| ES-Arbeiza22 | Spain | Arbeiza | 42.67 N | 2.07 W | infested | foragers, deformed wing bees, varroa | 2017 | 8 | *iberiensis* | none | 2.1 | 22 | 20 | H31-H40, H61-H70 | 10 deformed and 10 not deformed | | ND | ND | yes | H34, H35, H62, H67 | Andone Estonba/  Egoitz Galarza | University of the Basque Country (UPV/EHU), Spain |
| ES-Fraisoro17 | Spain | Fraisoro | 43.19 N | 2.06 W | infested | foragers, varroa | 2017 | 8 | *iberiensis* | none | no data | 17 | 11 | ND | ND | | ND | ND | ND | ND | Andone Estonba/  Egoitz Galarza | University of the Basque Country (UPV/EHU), Spain |
| ES-Fraisoro18 | Spain | Fraisoro | 43.19 N | 2.06 W | infested | foragers | 2017 | 8 | *iberiensis* | none | no data | 18 | 8 | ND | ND | | ND | ND | yes | ND | Andone Estonba/  Egoitz Galarza | University of the Basque Country (UPV/EHU), Spain |
| ES-Opakua(Araba)19 | Spain | Opakua | 42.83 N | 2.36 W | infested | foragers, varroa | 2017 | 8 | *iberiensis* | none | no data | 19 | 20 | ND | ND | | ND | ND | ND | ND | Andone Estonba/  Egoitz Galarza | University of the Basque Country (UPV/EHU), Spain |
| ES-Opakua(Araba)20 | Spain | Opakua | 42.83 N | 2.36 W | infested | foragers, deformed wing bees | 2017 | 8 | *iberiensis* | none | no data | 20 | 20 | H101-H110 | 10 not deformed | | ND | ND | yes | ND | Andone Estonba/  Egoitz Galarza | University of the Basque Country (UPV/EHU), Spain |
| FI-Åland69 | Finland | Åland | 60.18 N | 19.92 E | free | foragers | 2016 | NA | no data | none | 0 | 69 | 315 | ND | ND | | ND | ND | ND | ND | Eva Forsgreen | Swedish University of Agricultural Sciences (SLU), Sweden |
| FI-Åland70 | Finland | Åland | 60.18 N | 19.92 E | free | foragers | 2016 | NA | no data | none | 0 | 70 | 206 | ND | ND | | ND | ND | yes | ND | Eva Forsgreen | Swedish University of Agricultural Sciences (SLU), Sweden |
| FI-Åland71 | Finland | Åland | 60.18 N | 19.92 E | free | foragers | 2016 | NA | no data | none | 0 | 71 | 420 | ND | ND | | ND | ND | ND | ND | Eva Forsgreen | Swedish University of Agricultural Sciences (SLU), Sweden |
| FR-Caumont10 | France | Caumont-sur-Durance | 43.89 N | 4.94 E | infested | foragers, deformed wing bees, varroa | 2017 | 9 | no data | none | 2.2 | 10 | 20 | ND | ND | | ND | ND | yes | ND | Benjamin Basso | Institut technique et scientifique de l’apiculture et de la pollinisation (ITSAP), France |
| FR-Caumont6 | France | Caumont-sur-Durance | 43.89 N | 4.94 E | infested | foragers, deformed wing bees, varroa | 2017 | 9 | no data | none | 1.5 | 6 | 20 | H71-H80, H111-H117 | 7 deformed and 10 not deformed | | V11-V15 | 5 | ND | H73, H78, H111, H113 | Benjamin Basso | Institut technique et scientifique de l’apiculture et de la pollinisation (ITSAP), France |
| FR-Caumont7 | France | Caumont-sur-Durance | 43.89 N | 4.94 E | infested | foragers, deformed wing bees, varroa | 2017 | 9 | *carnica* | none | 4.6 | 7 | 20 | ND | ND | | ND | ND | yes | ND | Benjamin Basso | Institut technique et scientifique de l’apiculture et de la pollinisation (ITSAP), France |
| FR-Caumont8 | France | Caumont-sur-Durance | 43.89 N | 4.94 E | infested | foragers, deformed wing bees, varroa | 2017 | 9 | *carnica* | none | 6.6 | 8 | 20 | H196-H205, H206-H215 | 10 deformed and 10 not deformed | | V16-V20 | 5 | yes | ND | Benjamin Basso | Institut technique et scientifique de l’apiculture et de la pollinisation (ITSAP), France |
| FR-Caumont9 | France | Caumont-sur-Durance | 43.89 N | 4.94 E | infested | foragers, deformed wing bees, varroa | 2017 | 9 | no data | none | 8.1 | 9 | 20 | ND | ND | | ND | ND | yes | ND | Benjamin Basso | Institut technique et scientifique de l’apiculture et de la pollinisation (ITSAP), France |
| FR-Ouessant55 | France | Ouessant | 48.46 N | 5.09 W | free | foragers | 2011 | 4 | *mellifera* | none | 0 | 55 | 150 | ND | ND | | ND | ND | yes | ND | Olivier Lambert/  Monique l'Hostis | Ecole Nationale Vétérinaire, Agroalimentaire et de l'Alimentation Nantes-Atlantique (ONIRIS), France |
| FR-Ouessant56 | France | Ouessant | 48.46 N | 5.09 W | free | foragers | 2011 | 4 | *mellifera* | none | 0 | 56 | 150 | ND | ND | | ND | ND | ND | ND | Olivier Lambert/  Monique l'Hostis | Ecole Nationale Vétérinaire, Agroalimentaire et de l'Alimentation Nantes-Atlantique (ONIRIS), France |
| FR-Ouessant57 | France | Ouessant | 48.46 N | 5.09 W | free | foragers | 2011 | 4 | *mellifera* | none | 0 | 57 | 150 | ND | ND | | ND | ND | yes | ND | Olivier Lambert/  Monique l'Hostis | Ecole Nationale Vétérinaire, Agroalimentaire et de l'Alimentation Nantes-Atlantique (ONIRIS), France |
| FR-Ouessant58 | France | Ouessant | 48.46 N | 5.09 W | free | foragers | 2011 | 4 | *mellifera* | none | 0 | 58 | 150 | ND | ND | | ND | ND | ND | ND | Olivier Lambert/  Monique l'Hostis | Ecole Nationale Vétérinaire, Agroalimentaire et de l'Alimentation Nantes-Atlantique (ONIRIS), France |
| FR-Ouessant61 | France | Ouessant | 48.46 N | 5.09 W | free | foragers | 2011 | 4 | *mellifera* | none | 0 | 61 | 150 | ND | ND | | ND | ND | ND | ND | Olivier Lambert/  Monique l'Hostis | Ecole Nationale Vétérinaire, Agroalimentaire et de l'Alimentation Nantes-Atlantique (ONIRIS), France |
| FR-Ouessant62 | France | Ouessant | 48.46 N | 5.09 W | free | foragers | 2011 | 4 | *mellifera* | none | 0 | 62 | 150 | ND | ND | | ND | ND | yes | ND | Olivier Lambert/Monique l'Hostis | Ecole Nationale Vétérinaire, Agroalimentaire et de l'Alimentation Nantes-Atlantique (ONIRIS), France |
| FR-Sophia1 | France | Valbonne | 43.63 N | 7.04 E | infested | foragers, deformed wing bees, varroa | 2017 | 8 | *mellifera* | none | 3.2 | 1 | 20 | H171-H172 | 2 deformed | | ND | ND | yes | ND | Nicolas Cougoules | French agency for Food, Environmental and Occupational Health Safety (ANSES), France |
| FR-Sophia2 | France | Valbonne | 43.63 N | 7.04 E | infested | foragers, deformed wing bees, varroa | 2017 | 8 | hybrid | none | 3.4 | 2 | 11 | H173-H176 | 4 deformed | | V1-V5 | 5 | ND | H173, H174 | Nicolas Cougoules | French agency for Food, Environmental and Occupational Health Safety (ANSES), France |
| FR-Sophia3 | France | Valbonne | 43.63 N | 7.04 E | infested | foragers, deformed wing bees, varroa | 2017 | 8 | hybrid | none | 4.8 | 3 | 14 | ND | ND | | ND | ND | yes | ND | Nicolas Cougoules | French agency for Food, Environmental and Occupational Health Safety (ANSES), France |
| GR-DomenicoLavisa11 | Greece | Lavisa | 39.07 N | 21.82 E | infested | foragers, deformed wing bees, varroa | 2017 | 9 | *cecropia* | none | no data | 11 | 7 | ND | ND | | ND | ND | yes | ND | Fani Hatjina | Agricultural University of Athens (AUA), Greece |
| GR-DomenicoLavisa12 | Greece | Lavisa | 39.07 N | 21.82 E | infested | foragers, deformed wing bees, varroa | 2017 | 9 | *cecropia* | none | no data | 12 | 7 | H147-H150 | 4 not deformed | | V21-V25 | 5 | yes | H148, H149 | Fani Hatjina | Agricultural University of Athens (AUA), Greece |
| GR-MoudaniaChalkidiki13 | Greece | Chalkidiki | 40.37 N | 23.29 E | infested | foragers, varroa | 2017 | 9 | *cecropia* | none | 8 | 13 | 20 | ND | ND | | ND | ND | ND | ND | Fani Hatjina | Agricultural University of Athens (AUA), Greece |
| GR-MoudaniaChalkidiki14 | Greece | Chalkidiki | 40.37 N | 23.29 E | infested | foragers, varroa | 2017 | 9 | *cecropia* | none | 7 | 14 | 20 | ND | ND | | ND | ND | yes | ND | Fani Hatjina | Agricultural University of Athens (AUA), Greece |
| HR-HrvatskaKostajnica32 | Croatia | Hrvatska Kostajnica | 45.23 N | 16.54 E | infested | foragers, deformed wing bees, varroa | 2017 | 10 | *carnica* | amitraz | 11 | 32 | 20 | ND | ND | | ND | ND | yes | ND | Janja Filipi | University of Zadar, Croatia |
| HR-HrvatskaKostajnica50 | Croatia | Hrvatska Kostajnica | 45.23 N | 16.54 E | infested | foragers, deformed wing bees, varroa | 2017 | 10 | *carnica* | amitraz | 9 | 50 | 20 | H81-H90, H121-H128 | 4 deformed and 10 not deformed | | V51-V55 | 5 | yes | H83, H88, H121, H122, H128 | Janja Filipi | University of Zadar, Croatia |
| HR-Sisak39 | Croatia | Sisak | 45.49 N | 16.37 E | infested | foragers, varroa | 2017 | 9 | *carnica* | amitraz | 5 | 39 | 20 | ND | ND | | ND | ND | yes | ND | Janja Filipi | University of Zadar, Croatia |
| HR-Sisak47 | Croatia | Sisak | 45.49 N | 16.37 E | infested | foragers, varroa | 2017 | 9 | *carnica* | amitraz | 4 | 47 | 20 | ND | ND | | ND | ND | ND | ND | Janja Filipi | University of Zadar, Croatia |
| HR-Sisak48 | Croatia | Sisak | 45.49 N | 16.37 E | infested | foragers, varroa | 2017 | 9 | *carnica* | amitraz | 4 | 48 | 20 | ND | ND | | ND | ND | yes | ND | Janja Filipi | University of Zadar, Croatia |
| HR-Sisak49 | Croatia | Sisak | 45.49 N | 16.37 E | infested | foragers, varroa | 2017 | 9 | *carnica* | amitraz | 3 | 49 | 20 | ND | ND | | ND | ND | ND | ND | Janja Filipi | University of Zadar, Croatia |
| HR-UnijeKIR31 | Croatia | Unije | 44.63 N | 14.25 E | infested | RNA (10 bee heads from upper super) | 2011 | 5 | *macedonica* | none | 32.2 | KIR31 | 10 (bee heads) | ND | ND | | ND | ND | ND | ND | Marina Meixner | Landesbetrieb Landwirtschaft Hessen (LLH), Germany |
| HR-UnijeKIR32 | Croatia | Unije | 44.63 N | 14.25 E | infested | RNA (10 bee heads from upper super) | 2011 | 5 | *macedonica* | none | 59.8 | KIR32 | 10 (bee heads) | ND | ND | | ND | ND | ND | ND | Marina Meixner | Landesbetrieb Landwirtschaft Hessen (LLH), Germany |
| HR-UnijeKIR33 | Croatia | Unije | 44.63 N | 14.25 E | infested | RNA (10 bee heads from upper super) | 2011 | 5 | *mellifera* | none | 61.89 | KIR33 | 10 (bee heads) | ND | ND | | ND | ND | ND | ND | Marina Meixner | Landesbetrieb Landwirtschaft Hessen (LLH), Germany |
| HR-UnijeKIR34 | Croatia | Unije | 44.63 N | 14.25 E | infested | RNA (10 bee heads from upper super) | 2011 | 5 | *macedonica* | none | 43.78 | KIR34 | 10 (bee heads) | ND | ND | | ND | ND | ND | ND | Marina Meixner | Landesbetrieb Landwirtschaft Hessen (LLH), Germany |
| HR-UnijeKIR35 | Croatia | Unije | 44.63 N | 14.25 E | infested | RNA (10 bee heads from upper super) | 2011 | 5 | *mellifera* | none | no data | KIR35 | 10 (bee heads) | ND | ND | | ND | ND | ND | ND | Marina Meixner | Landesbetrieb Landwirtschaft Hessen (LLH), Germany |
| IT-Napoli59 | Italy | Napoli | 40.85 N | 14.27 E | infested | foragers, deformed wing bees, varroa | 2017 | 9 | *ligustica* | none | 9 | 59 | 20 | H161-H170 | 10 deformed | | ND | ND | yes | H162, H163 | Francesco Pennacchio/Gennaro Di Prisco | Università degli Studi di Napoli “Federico II” (UNINA), Italy |
| IT-Napoli60 | Italy | Napoli | 40.85 N | 14.27 E | infested | foragers, deformed wing bees, varroa | 2017 | 9 | *ligustica* | none | 10 | 60 | 20 | ND | ND | | ND | ND | ND | ND | Francesco Pennacchio/Gennaro Di Prisco | Università degli Studi di Napoli “Federico II” (UNINA), Italy |
| IT-Palermo4 | Italy | Palermo | 38.12 N | 13.36 E | infested | foragers, deformed wing bees, varroa | 2017 | 9 | *siciliana* | none | 7.1 | 4 | 20 | H141-H145 | 5 deformed | | V6-V10 | 5 | yes | H142, H143 | Raffaele Dall'Olio | Università degli Studi di Udine (UNIUD), Italy |
| IT-Palermo5 | Italy | Palermo | 38.12 N | 13.36 E | infested | foragers, deformed wing bees, varroa | 2017 | 9 | *siciliana* | none | 4.1 | 5 | 20 | ND | ND | | ND | ND | yes | ND | Raffaele Dall'Olio | Università degli Studi di Udine (UNIUD), Italy |
| IT-Palermo51 | Italy | Palermo | 38.12 N | 13.36 E | infested | foragers | 2017 | 9 | *siciliana* | amitraz | no data | 51 | 20 | ND | ND | | ND | ND | yes | ND | Raffaele Dall'Olio | Università degli Studi di Udine (UNIUD), Italy |
| IT-Palermo52 | Italy | Palermo | 38.12 N | 13.36 E | infested | foragers | 2017 | 9 | *siciliana* | amitraz | no data | 52 | 20 | ND | ND | | ND | ND | yes | ND | Raffaele Dall'Olio | Università degli Studi di Udine (UNIUD), Italy |
| MD-GhidighiciChisinau23 | Moldova | Ghidighici Chisinau | 47.15 N | 28.93 E | infested | foragers, deformed wing bees, varroa | 2017 | 10 | *carpatica* | none | no data | 23 | 20 | H151-H160 | 10 deformed | | ND | ND | yes | H153, H156 | Valentina Cebotari | Academy of Sciences of Moldova, Maldova |
| MD-GhidighiciChisinau24 | Moldova | Ghidighici Chisinau | 47.15 N | 28.93 E | infested | foragers, deformed wing bees, varroa | 2017 | 10 | *carpatica* | none | no data | 24 | 20 | ND | ND | | ND | ND | ND | ND | Valentina Cebotari | Academy of Sciences of Moldova, Maldova |
| MD-GhidighiciChisinau25 | Moldova | Ghidighici Chisinau | 47.15 N | 28.93 E | infested | foragers, deformed wing bees, varroa | 2017 | 10 | *carpatica* | none | no data | 25 | 20 | ND | ND | | ND | ND | yes | ND | Valentina Cebotari | Academy of Sciences of Moldova, Maldova |
| MD-GhidighiciChisinau26 | Moldova | Ghidighici Chisinau | 47.15 N | 28.93 E | infested | foragers, deformed wing bees, varroa | 2017 | 10 | *carpatica* | none | no data | 26 | 20 | ND | ND | | ND | ND | yes | ND | Valentina Cebotari | Academy of Sciences of Moldova, Maldova |
| MK-BitolaKIR36 | Macedonia | Bitola | 41.03 N | 21.33 E | infested | RNA (10 bee heads from upper super) | 2011 | 5 | *macedonica* | none | 31.1 | KIR36 | 10 (bee heads) | ND | ND | | ND | ND | ND | ND | Marina Meixner | Landesbetrieb Landwirtschaft Hessen (LLH), Germany |
| MK-BitolaKIR37 | Macedonia | Bitola | 41.03 N | 21.33 E | infested | RNA (10 bee heads from upper super) | 2010 | 10 | *macedonica* | none | 20.6 | KIR37 | 10 (bee heads) | ND | ND | | ND | ND | ND | ND | Marina Meixner | Landesbetrieb Landwirtschaft Hessen (LLH), Germany |
| MK-BitolaKIR38 | Macedonia | Bitola | 41.03 N | 21.33 E | infested | RNA (10 bee heads from upper super) | 2011 | 5 | *carnica* | none | 1.8 | KIR38 | 10 (bee heads) | ND | ND | | ND | ND | ND | ND | Marina Meixner | Landesbetrieb Landwirtschaft Hessen (LLH), Germany |
| MK-BitolaKIR39 | Macedonia | Bitola | 41.03 N | 21.33 E | infested | RNA (10 bee heads from upper super) | 2011 | 3 | *macedonica* | none | 16.4 | KIR39 | 10 (bee heads) | ND | ND | | ND | ND | ND | ND | Marina Meixner | Landesbetrieb Landwirtschaft Hessen (LLH), Germany |
| MK-BitolaKIR40 | Macedonia | Bitola | 41.03 N | 21.33 E | infested | RNA (10 bee heads from upper super) | 2011 | 3 | *macedonica* | none | 21.6 | KIR40 | 10 (bee heads) | ND | ND | | ND | ND | ND | ND | Marina Meixner | Landesbetrieb Landwirtschaft Hessen (LLH), Germany |
| MK-MalinoSvetiNikole15 | Macedonia | Malino Sveti Nikole | 41.95 N | 21.84 E | infested | foragers, deformed wing bees, varroa | 2017 | 9 | *macedonica* | none | 81 | 15 | 20 | H11-H20, H41-H47 | 10 deformed and 7 not deformed | | V26-V30 | 5 | yes | H11, H12, H14, H46, H47 | Borche Pavlov | MacBee, Macedonia |
| MK-MalinoSvetiNikole16 | Macedonia | Malino Sveti Nikole | 41.95 N | 21.84 E | infested | foragers, deformed wing bees, varroa | 2017 | 9 | *macedonica* | none | 67.8 | 16 | 20 | ND | ND | | ND | ND | yes | ND | Borche Pavlov | MacBee, Macedonia |
| MK-SDihovoBitola53 | Macedonia | Dihovo Bitola | 41.03 N | 21.26 E | infested | foragers, varroa | 2017 | 9 | *macedonica* | formic acid | 3.6 | 53 | 20 | ND | ND | | ND | ND | yes | ND | Borche Pavlov | MacBee, Macedonia |
| MK-SDihovoBitola54 | Macedonia | Dihovo Bitola | 41.03 N | 21.26 E | infested | foragers, varroa | 2017 | 9 | *macedonica* | formic acid | 2.5 | 54 | 20 | ND | ND | | ND | ND | yes | ND | Borche Pavlov | MacBee, Macedonia |
| NO-Buskerud64 | Norway | Buskerud | 60.48 N | 8.70 E | infested | foragers in quiazol | 2012 | 11 | *mellifera* | NA | no data | 64 | 8 | ND | ND | | ND | ND | ND | ND | Bjørn Dahle/Elisabeth Furuseth Hansen | Norwegian Beekeepers Association/Norwegian School of Veterinary Science, Norway |
| NO-Buskerud66 | Norway | Buskerud | 60.48 N | 8.70 E | infested | foragers in quiazol | 2014 | 11 | *mellifera* | NA | no data | 66 | 8 | ND | ND | | ND | ND | ND | ND | Bjørn Dahle/Elisabeth Furuseth Hansen | Norwegian Beekeepers Association/Norwegian School of Veterinary Science, Norway |
| NO-Østfold65 | Norway | Østfold | 59.26 N | 11.33 E | infested | foragers in quiazol | 2014 | 10 | *mellifera* | NA | no data | 65 | 8 | ND | ND | | ND | ND | yes | ND | Bjørn Dahle/Elisabeth Furuseth Hansen | Norwegian Beekeepers Association/Norwegian School of Veterinary Science, Norway |
| NO-Rogaland67 | Norway | Rogaland | 59.15 N | 6.01 E | infested | foragers in quiazol | 2014 | 11 | *mellifera* | NA | no data | 67 | 8 | ND | ND | | ND | ND | yes | ND | Bjørn Dahle/Elisabeth Furuseth Hansen | Norwegian Beekeepers Association/Norwegian School of Veterinary Science, Norway |
| NO-Vestfold63 | Norway | Vestfold | 59.17 N | 10.11 E | infested | foragers in quiazol | 2012 | 11 | *mellifera* | NA | no data | 63 | 8 | ND | ND | | ND | ND | yes | ND | Bjørn Dahle/Elisabeth Furuseth Hansen | Norwegian Beekeepers Association/Norwegian School of Veterinary Science, Norway |
| RO-Bucharest40 | Romania | Bucharest | 44.43 N | 26.10 E | infested | foragers, varroa | 2017 | 10 | *carpatica* | amitraz and fluvalinat | no data | 40 | 20 | ND | ND | | ND | ND | yes | ND | Eliza Cauia | Beekeeping Research and Development Institute (ICDA) , Romania |
| RO-Bucharest41 | Romania | Bucharest | 44.43 N | 26.10 E | infested | foragers, varroa | 2017 | 10 | *carpatica* | amitraz and fluvalinat | no data | 41 | 20 | ND | ND | | ND | ND | ND | ND | Eliza Cauia | Beekeeping Research and Development Institute (ICDA) , Romania |
| RO-Bucharest42 | Romania | Bucharest | 44.43 N | 26.10 E | infested | foragers, varroa | 2017 | 10 | *carpatica* | amitraz and fluvalinat | no data | 42 | 20 | ND | ND | | ND | ND | yes | ND | Eliza Cauia | Beekeeping Research and Development Institute (ICDA) , Romania |
| RO-Otopeni45 | Romania | Otopeni | 44.57 N | 26.08 E | infested | foragers, varroa | 2017 | 10 | *carpatica* | none | 3.9 | 45 | 20 | ND | ND | | ND | ND | yes | ND | Eliza Cauia | Beekeeping Research and Development Institute (ICDA) , Romania |
| RO-Otopeni46 | Romania | Otopeni | 44.57 N | 26.08 E | infested | foragers, varroa | 2017 | 10 | *carpatica* | none | 1.1 | 46 | 20 | ND | ND | | ND | ND | ND | ND | Eliza Cauia | Beekeeping Research and Development Institute (ICDA) , Romania |
| RO-TarguMures31 | Romania | TarguMures | 46.54 N | 24.55 E | infested | foragers, deformed wing bees, varroa | 2017 | 9 | *carpatica* | none | no data | 31 | 20 | H21-H30, H51-H60 | 10 deformed and 10 not deformed | | V41-V45 | 5 | yes | H23, H25, H26, H28, H53, H60 | Eliza Cauia | Beekeeping Research and Development Institute (ICDA) , Romania |
| RS-Belgrade29 | Serbia | Belgrade | 44.79 N | 20.45 E | infested | foragers, deformed wing bees, varroa | 2017 | 7 | *carnica* | none | 0.16 | 29 | 20 | H91-H97, H131-H140 | 7 not deformed | | V36-V40 | 5 | yes | H133, H137 | Nebosjsa Nedic/Kazimir Matovic | University of Belgrade, Serbia |
| RS-Belgrade30 | Serbia | Belgrade | 44.79 N | 20.45 E | infested | foragers, deformed wing bees, varroa | 2017 | 7 | *carnica* | none | 0.36 | 30 | 20 | ND | ND | | ND | ND | yes | ND | Nebosjsa Nedic/Kazimir Matovic | University of Belgrade, Serbia |
| RS-Kratjevo27 | Serbia | Kratjevo | 43.72 N | 20.69 E | infested | foragers, deformed wing bees, varroa | 2017 | 7 | *carnica* | none | 0.06 | 27 | 20 | ND | ND | | ND | ND | yes | ND | Nebosjsa Nedic/Kazimir Matovic | University of Belgrade, Serbia |
| RS-Kratjevo28 | Serbia | Kratjevo | 43.72 N | 20.69 E | infested | foragers, deformed wing bees, varroa | 2017 | 7 | *carnica* | none | 0.03 | 28 | 20 | ND | ND | | ND | ND | yes | ND | Nebosjsa Nedic/Kazimir Matovic | University of Belgrade, Serbia |
| RS-YrsacUlima33 | Serbia | Vrsac | 45.12 N | 21.29 E | infested | foragers, deformed wing bees, varroa | 2017 | 7 | *carnica* | essential repellent and vegetable oils | 0.18 | 33 | 20 | ND | ND | | ND | ND | ND | ND | Nebosjsa Nedic/Kazimir Matovic | University of Belgrade, Serbia |
| RS-YrsacUlima34 | Serbia | Vrsac | 45.12 N | 21.29 E | infested | foragers, deformed wing bees, varroa | 2017 | 7 | *carnica* | essential repellent and vegetable oils | 0.34 | 34 | 20 | ND | ND | | ND | ND | yes | ND | Nebosjsa Nedic/Kazimir Matovic | University of Belgrade, Serbia |
| UK-Colonsay72 | United Kingdom | Colonsay | 56.08 N | 6.21 W | free | foragers | 2017 | 5 | *mellifera* | none | 0 | 72 | 34 | ND | ND | | ND | ND | yes | ND | Ewan Campbell/Alan Bowman | University of Aberdeen (UABDN), United Kingdom |
| UK-Flaxton37 | United Kingdom | Flaxton | 53.96 N | 1.09 W | infested | foragers, deformed wing bees, varroa | 2017 | 10 | no data | oxalic acid | no data | 37 | 20 | ND | ND | | ND | ND | yes | ND | Maureen Wakefield / J Wilford | Food and Environment Research Agency (FERA), United Kingdom |
| UK-Flaxton38 | United Kingdom | Flaxton | 53.96 N | 1.09 W | infested | foragers, deformed wing bees, varroa | 2017 | 10 | hybrid buckfast | oxalic acid | no data | 38 | 20 | ND | ND | | ND | ND | yes | ND | Maureen Wakefield / J Wilford | Food and Environment Research Agency (FERA), United Kingdom |
| UK-SandHutton43 | United Kingdom | Sand Hutton | 54.02 N | 0.94 W | infested | foragers, deformed wing bees, varroa | 2017 | 10 | hybrid buckfast | amitraz and oxalic acid | no data | 43 | 19 | ND | ND | | ND | ND | yes | ND | Maureen Wakefield / J Wilford | Food and Environment Research Agency (FERA), United Kingdom |
| UK-SandHutton44 | United Kingdom | Sand Hutton | 54.02 N | 0.94 W | infested | foragers, deformed wing bees, varroa | 2017 | 10 | hybrid buckfast | amitraz and oxalic acid | no data | 44 | 20 | ND | ND | | ND | ND | ND | ND | Maureen Wakefield / J Wilford | Food and Environment Research Agency (FERA), United Kingdom |
| UK-SherburnInElmet35 | United Kingdom | Sherburn In Elmet | 53.79 N | 1.23 W | infested | foragers, deformed wing bees, varroa | 2017 | 10 | *mellifera* | formic acid | no data | 35 | 20 | H186-H191 | 6 deformed | | V46-V50 | 5 | yes | H188, H189 | Maureen Wakefield /Karyn Burke | Food and Environment Research Agency (FERA), United Kingdom |
| UK-SherburnInElmet36 | United Kingdom | Sherburn In Elmet | 53.79 N | 1.23 W | infested | foragers, deformed wing bees, varroa | 2017 | 10 | *mellifera* | formic acid | no data | 36 | 20 | H192-H194 | 3 deformed | | ND | ND | yes | H193, H194 | Maureen Wakefield /Karyn Burke | Food and Environment Research Agency (FERA), United Kingdom |
| **1.** Metadata are also available in the BioProject PRJNA1055031  **2.** ND: Not done | | | | | | | | | | | | | | | |  |  |  |  |  |  |  |

**Supplementary Table S6.** Primers, probes and recombinant plasmids used for DWV-A and DWV-B quantification by RT-qPCR according to Schurr et al., 2019 ^43^.

| **Virus** | **Forward primer (5’ -> 3’)** | **Reverse primer (5’ -> 3’)** | **Probe (5’ -> 3’)** | **Recombinant plasmid** |
| --- | --- | --- | --- | --- |
| DWV-A | GCGGCTAAGATTGTAAATTG | GTGACTAGCATAACCATGATTA | (6-Fam)CCTTGACCAGTAGA CACAGCATC(Tamra) | pC1 |
| DWV-B | GGTCTGAAGCGAAAATAG | CTAGCATATCCATGATTATAAAC | (6-Fam)CCTTGTCCAGTAGA TACAGCATCACA(Tamra) | pFab1 |

**A**


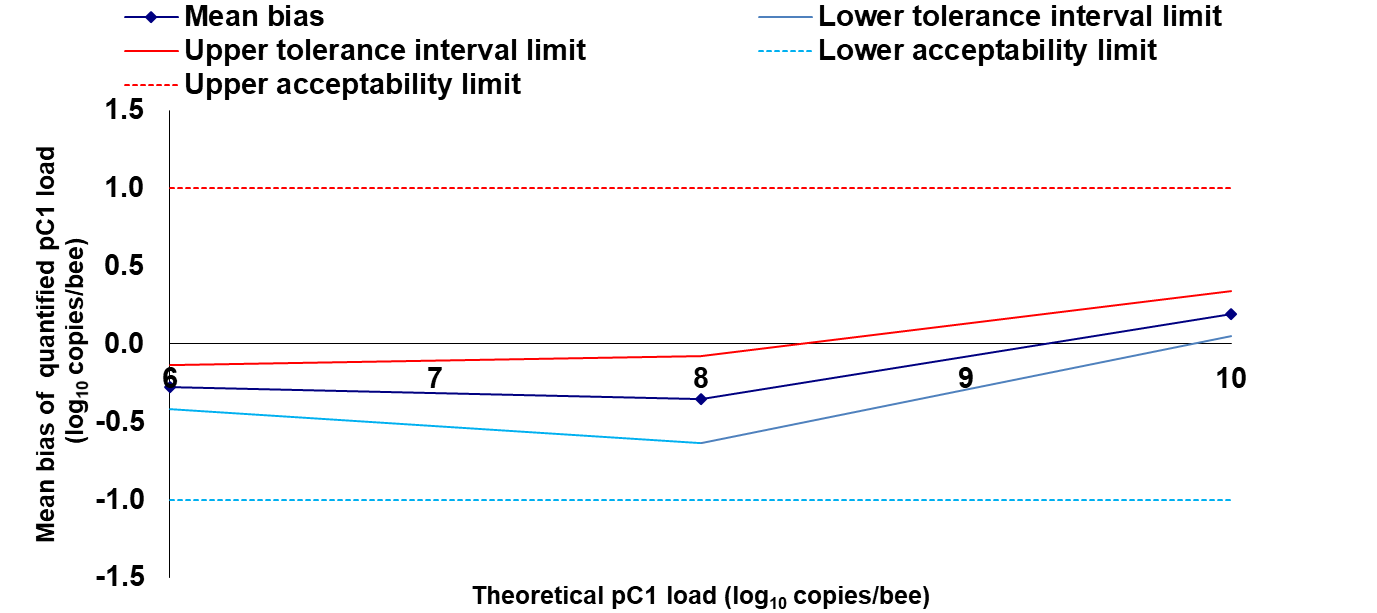


**B**

**
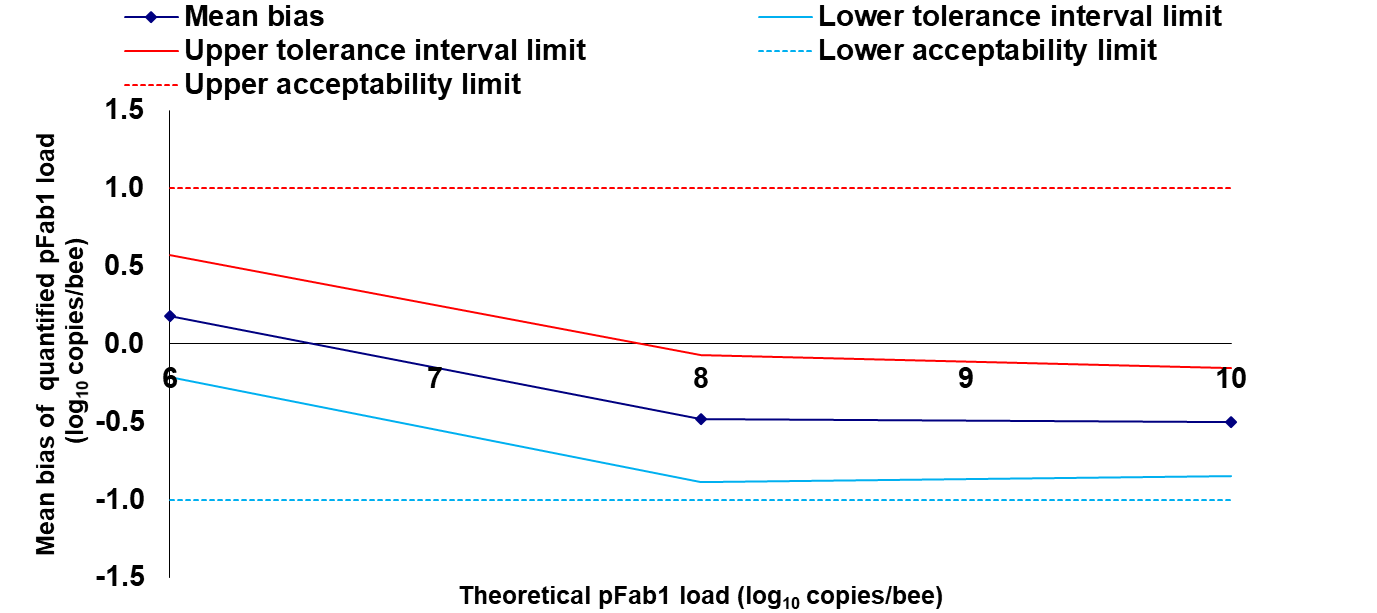
**

**Supplementary Figure S7.** Accuracy profiles of the DWV-A and DWV-B quantification methods. The assessment of the accuracy of the viral loads quantified in pooled samples of whole bees by DWV-A and DWV-B RT qPCR was based on the construction and interpretation of accuracy profiles. Crushed whole bee samples were spiked with pC1 and pFab1 recombinant plasmids including a DWV-A or DWV-B VP3 coding sequence, respectively, and processed for viral quantification. Three plasmid load levels (10^6^, 10^8^ and 10^10^ copies/bee or head) were used to determine the standard deviation of reproducibility (SD_R_), the tolerance interval (± 2xSD_R_), and the mean bias between the theoretical value and the mean of the values obtained from six results per load level. Both RT-qPCR methods quantified the recombinant plasmids within the limits of ± 1.0 log_10_ copies per bee. **A**, quantification accuracy of DWV-A RT-qPCR. **B**, quantification accuracy of DWV-B RT-qPCR.

| **Supplementary Table S7.** Reference genome of honey bee viruses. | | |
| --- | --- | --- |
| **Virus name** | **NCBI ID** | **Genome size (nt)** |
| LSV1 | HQ871931 | 5904 |
| LSV2 | HQ888865 | 5910 |
| VDV1 (DWV-B) | AY251269 | 10112 |
| DWV (DWV-A) | AY292384 | 10166 |
| ABPV | AF486072 | 9459 |
| BQCV | KY741959 | 8440 |
| SBV | MG545286 | 8861 |
| ARV-1 | KY354230 | 14598 |
| ARV-2 | KY354233 | 14028 |
| CBPV-RNA1 | NC_010711 | 3674 |
| CBPV-RNA2 | NC_010712 | 2305 |
| IAPV | NC_009025 | 9499 |
| KBV | NC_004807 | 9524 |
| SBPV | NC_014137 | 9505 |

| **Supplementary Table S8.** DWV-A and DWV-B genomes used to align the cleaned reads obtained from honey bee samples. | | | | | |
| --- | --- | --- | --- | --- | --- |
| **Sample code** | **DWV-A reference genome** | **DWV-B reference genome** | **Lumpy-smoove analysis** | **Min element thresholds used in LUMPY** |  |
| DE-HohenNeuendorf_2015_H3 | AY292384.1 | AY251269.2 | completed | 500 |  |
| DE-HohenNeuendorf_2015_H6 | AY292384.1 | AY251269.2 | completed | 500 |  |
| ES-Arbeiza21_2017_P | AY292384.1 | AY251269.2 | completed | 500 |  |
| ES-Arbeiza22_2017_H34 | AY292384.1 | AY251269.2 | completed | 500 |  |
| ES-Arbeiza22_2017_H35 | KU847397.1 | AY251269.2 | completed | 500 |  |
| ES-Arbeiza22_2017_H62 | AY292384.1 | AY251269.2 | completed | 500 |  |
| ES-Arbeiza22_2017_H67 | AY292384.1 | AY251269.2 | not completed | NA |  |
| ES-Arbeiza22_2017_P | AY292384.1 | AY251269.2 | completed | 500 |  |
| ES-Fraisoro18_2017_P | AY292384.1 | AY251269.2 | not completed | NA |  |
| ES-Opakua(Araba)20_2017_P | AY292384.1 | AY251269.2 | completed | 50 |  |
| FI-Åland70_2016_P | KU847397.1 | AY251269.2 | not completed | NA |  |
| FR-Caumont10_2017_P | AY292384.1 | AY251269.2 | completed | 300 |  |
| FR-Caumont6_2017_H111 | AY292384.1 | AY251269.2 | completed | 300 |  |
| FR-Caumont6_2017_H113 | KU847397.1 | AY251269.2 | completed | 500 |  |
| FR-Caumont6_2017_H73 | GU109335.1 | AY251269.2 | not completed | NA |  |
| FR-Caumont6_2017_H78 | AY292384.1 | AY251269.2 | completed | 300 |  |
| FR-Caumont7_2017_P | AY292384.1 | AY251269.2 | completed | 300 |  |
| FR-Caumont8_2017_P | AY292384.1 | AY251269.2 | completed | 300 |  |
| FR-Caumont9_2017_P | KU847397.1 | AY251269.2 | completed | 300 |  |
| FR-Ouessant55_2011_P | None | AY251269.2 | not completed | NA |  |
| FR-Ouessant57_2011_P | None | None | not completed | NA |  |
| FR-Ouessant62_2011_P | AY292384.1 | AY251269.2 | not completed | NA |  |
| FR-Sophia1_2017_P | None | AY251269.2 | not completed | NA |  |
| FR-Sophia2_2017_H173 | None | AY251269.2 | not completed | NA |  |
| FR-Sophia2_2017_H174 | GU109335.1 | AY251269.2 | completed | 500 |  |
| FR-Sophia3_2017_P | AY292384.1 | AY251269.2 | completed | 500 |  |
| GR-DomenicoLavisa11_2017_P | AY292384.1 | AY251269.2 | completed | 500 |  |
| GR-DomenicoLavisa12_2017_P | AY292384.1 | AY251269.2 | not completed | NA |  |
| GR-MoudaniaChalkidiki14_2017_P | None | AY251269.2 | not completed | NA |  |
| HR-HrvatskaKostajnica32_2017_P | AY292384.1 | AY251269.2 | completed | 300 |  |
| HR-HrvatskaKostajnica50_2017_H121 | AY292384.1 | AY251269.2 | completed | 300 |  |
| HR-HrvatskaKostajnica50_2017_H122 | AY292384.1 | AY251269.2 | completed | 500 |  |
| HR-HrvatskaKostajnica50_2017_H128 | AY292384.1 | AY251269.2 | completed | 500 |  |
| HR-HrvatskaKostajnica50_2017_H83 | AY292384.1 | AY251269.2 | completed | 500 |  |
| HR-HrvatskaKostajnica50_2017_H88 | AY292384.1 | AY251269.2 | completed | 300 |  |
| HR-HrvatskaKostajnica50_2017_P | KT004425.1 | AY251269.2 | not completed | NA |  |
| HR-Sisak39_2017_P | AY292384.1 | AY251269.2 | completed | 500 |  |
| HR-Sisak48_2017_P | AY292384.1 | AY251269.2 | completed | 50 |  |
| IT-Napoli59_2017_H162 | AY292384.1 | AY251269.2 | completed | 50 |  |
| IT-Napoli59_2017_H162 | AY292384.1 | AY251269.2 | completed | 500 |  |
| IT-Napoli59_2017_H163 | KU847397.1 | AY251269.2 | completed | 500 |  |
| IT-Napoli59_2017_H163 | AY292384.1 | AY251269.2 | not completed | NA |  |
| IT-Napoli59_2017_P | AY292384.1 | AY251269.2 | completed | 50 |  |
| IT-Palermo4_2017_H142 | KT004425.1 | AY251269.2 | completed | 500 |  |
| IT-Palermo4_2017_H143 | AY292384.1 | AY251269.2 | completed | 500 |  |
| IT-Palermo4_2017_P | AY292384.1 | AY251269.2 | completed | 500 |  |
| IT-Palermo5_2017_P | AY292384.1 | None | not completed | NA |  |
| IT-Palermo51_2017_P | AY292384.1 | AY251269.2 | completed | 300 |  |
| IT-Palermo52_2017_P | KT004425.1 | AY251269.2 | completed | 500 |  |
| MD-GhidighiciChisinau23_2017_H153 | AY292384.1 | AY251269.2 | completed | 500 |  |
| MD-GhidighiciChisinau23_2017_H156 | AY292384.1 | AY251269.2 | completed | 500 |  |
| MD-GhidighiciChisinau23_2017_P | AY292384.1 | AY251269.2 | completed | 50 |  |
| MD-GhidighiciChisinau25_2017_P | AY292384.1 | None | not completed | NA |  |
| MD-GhidighiciChisinau26_2017_P | AY292384.1 | AY251269.2 | completed | 500 |  |
| MK-MalinoSvetiNikole15_2017_H11 | KT004425.1 | AY251269.2 | completed | 500 |  |
| MK-MalinoSvetiNikole15_2017_H12 | GU109335.1 | AY251269.2 | completed | 500 |  |
| MK-MalinoSvetiNikole15_2017_H14 | AY292384.1 | AY251269.2 | completed | 50 |  |
| MK-MalinoSvetiNikole15_2017_H46 | GU109335.1 | AY251269.2 | completed | 500 |  |
| MK-MalinoSvetiNikole15_2017_H47 | GU109335.1 | AY251269.2 | completed | 500 |  |
| MK-MalinoSvetiNikole15_2017_P | GU109335.1 | AY251269.2 | completed | 50 |  |
| MK-MalinoSvetiNikole16_2017_P | AY292384.1 | AY251269.2 | completed | 300 |  |
| MK-SDihovoBitola53_2017_P | AY292384.1 | AY251269.2 | not completed | NA |  |
| MK-SDihovoBitola54_2017_P | None | None | not completed | NA |  |
| NO-Østfold65_2014_P | AY292384.1 | AY251269.2 | not completed | NA |  |
| NO-Rogaland67_2014_P | AY292384.1 | AY251269.2 | not completed | NA |  |
| NO-Vestfold63_2012_P | AY292384.1 | AY251269.2 | not completed | NA |  |
| RO-Bucharest40_2017_P | AY292384.1 | AY251269.2 | completed | 500 |  |
| RO-Bucharest42_2017_P | AY292384.1 | AY251269.2 | not completed | NA |  |
| RO-Otopeni45_2017_P | AY292384.1 | AY251269.2 | not completed | NA |  |
| RO-TarguMures31_2017_H23 | AY292384.1 | AY251269.2 | completed | 500 |  |
| RO-TarguMures31_2017_H25 | AY292384.1 | AY251269.2 | completed | 500 |  |
| RO-TarguMures31_2017_H26 | AY292384.1 | AY251269.2 | completed | 500 |  |
| RO-TarguMures31_2017_H28 | AY292384.1 | AY251269.2 | completed | 500 |  |
| RO-TarguMures31_2017_H53 | KU847397.1 | None | not completed | NA |  |
| RO-TarguMures31_2017_H60 | AY292384.1 | AY251269.2 | completed | 500 |  |
| RO-TarguMures31_2017_P | AY292384.1 | AY251269.2 | not completed | NA |  |
| RS-Belgrade29_2017_H133 | AY292384.1 | AY251269.2 | not completed | NA |  |
| RS-Belgrade29_2017_H137 | None | AY251269.2 | not completed | NA |  |
| RS-Belgrade29_2017_P | None | AY251269.2 | not completed | NA |  |
| RS-Belgrade30_2017_P | AY292384.1 | AY251269.2 | completed | 300 |  |
| RS-Kratjevo27_2017_P | AY292384.1 | AY251269.2 | not completed | NA |  |
| RS-Kratjevo28_2017_P | None | AY251269.2 | not completed | NA |  |
| RS-YrsacUlima34_2017_P | AY292384.1 | AY251269.2 | not completed | NA |  |
| UK-Colonsay72_2017_P | AY292384.1 | AY251269.2 | not completed | NA |  |
| UK-Flaxton37_2017_P | AY292384.1 | AY251269.2 | completed | 300 |  |
| UK-Flaxton38_2017_P | AY292384.1 | AY251269.2 | completed | 500 |  |
| UK-SandHutton43_2017_P | JQ413340.1 | AY251269.2 | completed | 50 |  |
| UK-SherburnInElmet35_2017_H188 | GU109335.1 | AY251269.2 | completed | 500 |  |
| UK-SherburnInElmet35_2017_H189 | GU109335.1 | AY251269.2 | completed | 500 |  |
| UK-SherburnInElmet35_2017_P | AY292384.1 | AY251269.2 | completed | 500 |  |
| UK-SherburnInElmet36_2017_H193 | GU109335.1 | AY251269.2 | completed | 500 |  |
| UK-SherburnInElmet36_2017_H194 | GU109335.1 | AY251269.2 | completed | 500 |  |
| UK-SherburnInElmet36_2017_P | AY292384.1 | AY251269.2 | completed | 500 |  |

**Supplementary Figure S8.** DWV genome reconstruction pipeline applied to a study sample (DE-HohenNeuendorf_2015_H3). SPAdes was used to create contigs by *de novo* assembly. Contigs were mapped to the most homologous DWV reference genotypes with BWA-MEM and visualized using the Integrative Genomics Viewer (IGV). Output recombination junctions from both LUMPY and ViReMa combined with read coverage profiles were used to guide genome reconstruction according to overlapping contig sequences.

| **Supplementary Table S9.** DWV reference genomes used for the phylogenetic analysis. | | | |
| --- | --- | --- | --- |
| **DWV type** | **Country** | **NCBI Id** | **Sample name** |
| DWV-A | South-Korea | JX878305 | KR_2012_JX878305 |
| DWV-A | South-Korea | JX878304 | KR_2012_JX878304 |
| DWV-A | France | KX373899 | FR_2013_KX373899 |
| DWV-A | Chili | JQ413340 | CL_2001_JQ413340 |
| DWV-A | United State of America | AY292384 | USA_2001_AY292384 |
| DWV-A | Italy | AJ489744 | IT_2000_AJ489744 |
| DWV-A | United State of America | KT004425 | USA_2014_KT004425 |
| DWV-A | Austria | KU847397 | AT_2014_KU847397 |
| DWV-A | United-Kingdom | GU109335 | UK_2009_GU109335 |
| DWV-A | China | MF770715 | CN_2017_MF770715 |
| DWV-A | New Zeland | MF623172 | NZ_2015_MF623172 |
| DWV-A | United State of America | MG831200 | USA_2015_MG831200 |
| DWV-A | United State of America | MG831201 | USA_2015_MG831201 |
| DWV-A | United State of America | MG831202 | USA_2015_MG831202 |
| DWV-A | United State of America | MG831203 | USA_2015_MG831203 |
| DWV-A | United State of America | MG831204 | USA_2015_MG831204 |
| DWV-A | Sweden | MH267695 | SE_2009_MH267695 |
| DWV-A | Sweden | MH267696 | SE_2009_MH267696 |
| DWV-B | Netherlands | AY251269 | NL_2001_AY251269 |
| DWV-B | Belgium | KX783225 | BE_2012_KX782225 |
| DWV-C | United-Kingdom | ERS657949 | UK_2006_ERS657948 |
| KV | Japan | AB070959 | JPN_2000_AB070959 |
| Recombinant | France | KX373900 | FR_2013_KX373900 |
| Recombinant | United-Kingdom | KJ437447 | UK_2013_KJ437447 |
| Recombinant | United-Kingdom | HM067438 | UK_2009_HM067438 |
| Recombinant | United-Kingdom | HM067437 | UK_2009_HM067437 |
| DWV-A | United States of America | NA | USA_2017_FL22G1 |
| DWV-A | United States of America | NA | USA_2015_MD7G1 |
| DWV-A | United States of America | NA | USA_2015_MD12G1 |
| DWV-A | United States of America | NA | USA_2015_MD2G1 |
| DWV-B | United States of America | NA | USA_2015_MD12G2 |
| DWV-B | United States of America | NA | USA_2015_MD2G2 |
| DWV-B | United States of America | NA | USA_2015_MD7G2 |
| DWV-B | United States of America | NA | USA_2017_FL22G2 |
